# A novel hybrid genetic algorithm and Nelder-Mead approach and it’s application for parameter estimation

**DOI:** 10.12688/f1000research.154598.1

**Published:** 2024-09-19

**Authors:** Neha Majhi, Rajashree Mishra

**Affiliations:** 1 Mathematics, Kalinga Institute of Industrial Technology, Bhubaneswar, Odisha, 751024, India

**Keywords:** Genetic Algorithm, Maximum Likelihood Estimation, Nelder-Mead algorithm, Power Density, Weibull Distribution, Wind speed analysis

## Abstract

**Background:**

Traditional optimization methods often struggle to balance global exploration and local refinement, particularly in complex real-world problems. To address this challenge, we introduce a novel hybrid optimization strategy that integrates the Nelder-Mead (NM) technique and the Genetic Algorithm (GA), named the Genetic and Nelder-Mead Algorithm (GANMA). This hybrid approach aims to enhance performance across various benchmark functions and parameter estimation tasks.

**Methods:**

GANMA combines the global search capabilities of GA with the local refinement strength of NM. It is first tested on 15 benchmark functions commonly used to evaluate optimization strategies. The effectiveness of GANMA is also demonstrated through its application to parameter estimation problems, showcasing its practical utility in real-world scenarios.

**Results:**

GANMA outperforms traditional optimization methods in terms of robustness, convergence speed, and solution quality. The hybrid algorithm excels across different function landscapes, including those with high dimensionality and multimodality, which are often encountered in real-world optimization issues. Additionally, GANMA improves model accuracy and interpretability in parameter estimation tasks, enhancing both model fitting and prediction.

**Conclusions:**

GANMA proves to be a flexible and powerful optimization method suitable for both benchmark optimization and real-world parameter estimation challenges. Its capability to efficiently explore parameter spaces and refine solutions makes it a promising tool for scientific, engineering, and economic applications. GANMA offers a valuable solution for improving model performance and effectively handling complex optimization problems.

## 1. Introduction

In the continuous pursuit of optimization, where achieving the finest possible outcomes with utmost efficiency and accuracy is crucial, the fusion of diverse methodologies frequently yields superior solutions. Optimization algorithms are always looking for ways to improve efficiency and robustness, encouraging professionals and scholars to investigate novel ideas that happen to be mostly inspired by nature or mathematical concepts. Hybridization in optimization algorithms has garnered significant attention in recent years, offering a potent means to enhance efficacy and efficiency. Among these methodologies, Genetic Algorithms (GA) and the Nelder-Mead Simplex Algorithm (NM) emerge as prominent contenders, each boasting distinct advantages and applications. However, the fusion of these theories has recently proven to be an enticing strategy for enhancing optimization capabilities across various domains.

Inspired by evolution and natural selection, genetic algorithms operate by repeatedly developing a population of potential solutions over a series of generations. The concepts of genetic recombination and survival of the fittest are collectively mirrored by the selection, crossover, and mutation operators involved in this evolutionary process. GAs are a popular choice in various industries, including engineering, finance, and biology, because of their impressive effectiveness in solving complicated, high-dimensional optimization problems with non-linear and multimodal objective functions.

The Nelder-Mead Simplex Algorithm, on the other hand, provides a geometric method for repeatedly refining a simplex—a multi-dimensional geometric shape in the direction of the ideal solution. Its foundation is mathematical optimization. Nelder- Mead algorithms are especially well-suited for problems with few variables or smooth objective functions because, in contrast to GAs, which rely on a population-based approach, they operate on a single point or simplex at each iteration. Due to its ease of use, simplicity, and speedy convergence to local optima, it now has a deserving place within the optimization toolbox.

Individually, both GA and NM have strengths and limits that make them appropriate for specific optimization scenarios. GA excels in global exploration, utilizing population variety to explore large solution spaces and avoid local optima. On the other hand, NM excels at local refinement, expertly traversing convex and smooth terrain to locate specific optima. By fusing two different but complementary methods, the hybridization of GA and NM aims to get the best of both worlds and encourage synergy in optimization techniques.

Many GA combinations have been proposed in the literature before our study; we briefly mention a few of them below. A hybrid algorithm for the collocation approach of boundary value problem-solving was presented by Nikos E. Mastorakis. ^1^ A combined NM and GA was proposed by Durand and Alliot, ^2^ and evaluated on two benchmark functions where the employment of either technique alone is useless. Their research demonstrates the speed boost that NM provides. The trade-off in global optimization between computing time, precision, and dependability was initially noted by Renders and Flasse. ^3^ By fusing a genetic algorithm, particle swarm optimization, and the Nelder-Mead simplex search methodology, Fanetal. ^4^ have created a revolutionary strategy. Their research was focused on determining the global best solution for non-linear continuous variable functions. They have shown how their technique performs comparably on 10 test issues in their study article. Hwang and He ^5^ have presented an inventive adaptive real-parameter simulated annealing genetic method in a similar spirit. The benefits of simulated annealing and genetic algorithms are both effectively maintained by this approach. A comparative examination of the algorithm’s performance on two engineering design issues and sixteen benchmark problems has been provided by the authors.

Many industries are interested in using the Nelder-Mead Simplex Algorithm (NM) working together with Genetic Algorithms (GA), including bioinformatics, finance, and engineering. Combining these methods provides a potent method of resolving challenging optimization issues in engineering, where designs are complicated and rules are demanding. Combining GA with NM helps improve portfolio management and risk assessment in the financial industry, where on-time and correct choices are essential. Similarly, in bioinformatics, where understanding biology relies on smart computer methods, hybrid algorithms speed up tasks like genomic analysis and drug discovery. This article explores how combining NM and GA enhances both, highlighting how they work together to solve real-world issues. In this paper, the GANMA algorithm has been tested on fifteen benchmark problems on three different dimensions (10, 20, and 30). According to the results from the experiment, the suggested GANMA algorithm is a promising one that can quickly find the best or almost the best solution for the majority of the functions that were examined.

The remaining portion of the research study is structured as follows: Section 2 covers the fundamentals of the Genetic Algorithm and Nelder-Mead simplex search; comprehensive information about the suggested techniques is provided in Section 3; and the performance and result analysis of the proposed algorithm is offered in Section 4. Information about parameter estimation and the Weibull distribution is shown in Section 5, information regarding estimation techniques is presented in Section 6, and a simulation test and result regarding an estimation approach is shown in Section 7. Two real-world Wind speed data sets are provided as examples in Section 8 to show how effective the suggested technique is. Section 9 finally has some concluding observations.

## 2. Overview of GA, and NM

A brief overview of GA, and NM have been described below.

### 2.1 Real-Coded Genetic Algorithm (GA)

GA is an approach to heuristic search. The ideas of the biological evolution of species serve as its inspiration. In contrast to traditional optimization methods, GA ^6^^,^^7^ starts with a collection of starting solutions known as chromosomes.

Genetic algorithms (GAs) work by continually improving solutions based on their fitness, which measures how well they solve a problem. Unlike some traditional methods, GAs don’t assume anything about the problem, like whether it’s smooth or has just one best solution. Instead, they explore different possibilities to find good solutions, even in complex situations where there might be many equally good answers. GAs have been used successfully in many difficult optimization problems. They often work better than traditional methods, especially when there are multiple equally good solutions. This flexibility and ability to handle complex situations make GAs a valuable tool for solving optimization problems in various fields.

Following is a summary of the GA stages in this study:

I. Initialization:
  • First, create a vector of real values for each variable between predefined ranges. This vector will represent the initial population of individuals.
II. Evaluation:
  • Evaluate the fitness of each individual using an objective function.
III. Selection:
  • Select individuals from the population to create a mating pool based on their fitness values.
IV. Crossover (Recombination):
  • Pair selected individuals and perform crossover to create offspring by blending or combining their real values.
V. Mutation:
  • Introduce random changes to the real values of offspring to promote exploration of the search space.
VI. Combining Populations:
  • Combine the offspring generated from crossover and mutation with the initial population.
VII. Sorting:
  • The combined population is sorted based on their fitness levels, with the most fit people having the lowest fitness values.
VIII. Elitism:
  • Keep only the top half of the sorted population, discarding the bottom half. This ensures that the best-performing individuals from the previous generation are preserved for next generation.
IX. Termination:
  • Steps 2 through 8 should be repeated for the designated number of generations or until a termination criterion—such as achieving a maximum number of iterations or reaching a certain fitness level is satisfied.

This approach with elitism helps maintain diversity in the population while ensuring that the best individuals are preserved across generations, ultimately leading to the discovery of better solutions in the optimization process. Real-coded genetic algorithms are suitable for optimization problems with continuous decision variables and offer advantages such as direct representation of real-valued solutions, robustness, and ability to handle high-dimensional search spaces.

### 2.2 Nelder–Mead Simplex Search Method (NM)

The simplex search technique addresses basic unconstrained minimization cases like Olsson & Nelson, ^8^ nonlinear least squares, nonlinear simultaneous equations, and function minimization. Spendley, Hext, and Himsworth ^9^ initially suggested the simplex search technique, which was later improved upon by Nelder and Mead. ^10^

The steps of the Nelder-Mead ^10^^,^^11^ algorithm are summarized in as follows:

I. Initialization:
  • A simplex is a collection of *n* + 1 vertices in a *n* dimensional space. These vertices can be deliberately selected or created at random.
  • At every simplex vertex, evaluate the objective of the function.
II. Ordering:
  • Order the vertices based on their corresponding function values.
  • Let *x*_1_ *, x*_2_ *, …, x*_*n*+1_ denote the vertices such that *f* ( *x*_1_) ≤ *f* ( *x*_2_) ≤ *…* ≤ *f* ( *x*_*n*+1_).
III. Centroid:
  • Calculate the centroid of each vertex, except the worst (highest) one:
$$x_{\text{centroid}}=\frac{1}{n}\sum_{i=1}^{n}x_{i}$$
IV. Reflection:
  • Reflect the worst vertex (highest) through the centroid to obtain a trial point
$$x_{r}=x_{\text{centroid}}+\alpha \left(x_{\text{centroid}}-x_{n+1}\right)$$
    where *α* is a reflection coefficient, typically set to 1
  • Evaluate the objective function at $x_{r}$ .
V. Expansion:
  • Expanding further should be considered if the reflected point $x_{e}$ is superior to the second-worst vertex:
$$x_{e}=x_{\text{centroid}}+\gamma \left(x_{r}-x_{\text{centroid}}\right)$$
    where γ is an expansion coefficient, usually γ>1

  • Evaluate the objective function at $x_{e}$ .
VI. Contraction:
  • Contraction should be done if the reflected point $x_{r}$ is worse than the worst vertex:
    – Outside contraction: $x_{c}=x_{\text{centroid}}+\rho \left(x_{r}-x_{\text{centroid}}\right)$

    – Inside contraction: $\;x_{c}=x_{\text{centroid}}+\rho \left(x_{r}-x_{\text{centroid}}\right)$

      where *ρ* is a contraction coefficient, typically 0 *< ρ <*1 (typically 0.5).
  • Evaluate the objective function at $x_{c}$ .
VII. Update simplex:
  • Replace the worst vertex with the new trial point if it improves the function value.
VIII. Termination:
  • Till a termination criterion such as a maximum number of iterations, a small modification in step size, or a slight modification in the function value is satisfied, repeat the steps described above.

The algorithm converges when the simplex becomes sufficiently small or when the function values at the vertices are close to each other. The choice of parameters *α*, *γ*, and *ρ* can significantly affect the performance of the algorithm and may need to be tuned based on the problem characteristics.

## 3. Methods

### 3.1 Motivation

The combination of Genetic Algorithms (GA) with the Nelder-Mead simplex algorithm (NM) is driven by their supportive characteristics in both global exploration and local exploitation. GA is a population-based technique that effectively explores diverse sections of the search space, although fine-tuning solutions at local optima may provide issues. In contrast, it requires greater capacity for worldwide investigation. Combining both methods intends to take advantage of the characteristics of both algorithms, resulting in a more balanced and efficient optimization process. This hybridization method has the potential to improve convergence rates, solution quality, and robustness, making it a compelling choice for handling complicated optimization problems across several domains.

### 3.2 Genetic algorithm with Nelder-Mead Simplex Search (GANMA)

The suggested algorithm’s (GANMA) stages are summed up as follows:

I. Initialization:
  • Generate an initial population of solutions for the GA.
II. Evaluation:
  • Analyze each solution’s objective function within the population.
III. Genetic Algorithm (GA) Cycle:
  • Selection: Select a parent from the current population. Selection techniques that are often used include rank-based, roulette wheel, and tournament selection.
  • Crossover: Perform crossover to create offspring solutions. Since this is a real coded GA, a common method is the arithmetic crossover or simulated binary crossover.
  • Mutation: Apply mutation operators to the offspring solutions. Here is where the Nelder-Mead simplex algorithm comes into play. After mutation, the simplex is formed around the mutated solutions.
  • Elitism: Combine the initial population and offspring after mutation, then calculate the mean combination. Sort the combined population according to their fitness and keep the first half population while rejecting the other half.
  • Replacement: Replace the initial population with the best half from the previous step.
IV. Nelder-Mead Simplex Algorithm:
  • Define the simplex for the NM algorithm. This can be done by selecting a set of initial points around the best solution found by the GA so far.
  • Reflection: Take the centroid of the remaining points and reflect the worst point of the simplex.
  • Expansion: Attempt to extend the simplex in that direction if the reflected point is superior to the second-worst but not superior to the greatest.
    • Contraction: If neither reflection nor expansion produces a better point, contract the simplex towards the best point.
    • Update the simplex based on the chosen operation (reflection, expansion, contraction, or shrinkage).
    • Repeat the above steps until convergence criteria are met.
V. Termination:
  • Repeat the GA cycle and NM algorithm until a termination criterion is satisfied. This could be a maximum number of iterations, reaching a specific fitness threshold, or convergence of the simplex.
VI. Output:
  • The best solution found after the termination criterion is met.
  • Apply the Nelder-Mead simplex algorithm to the best solution.
VII. Optimal:
  • The best solution found after the NM algorithm.

By combining GA with NM in this way, you leverage the global exploration capability of the GA with the local refinement ability of the NM algorithm, potentially leading to improved convergence and robustness in optimization tasks.

The pseudo-code for the hybridization of the GA and Nelder-Mead simplex algorithm is presented in Algorithm. ^12^

Algorithm **Combination of GA and Nelder-Mead.**

1: Initialize GA parameters (size of population, rate of mutation, rate of crossover, number of generations)

2: Initial population

3: **while** termination condition is not met **do**

4:   Evaluate each individual’s current level of fitness

5:   Select parents (using tournament selection) for crossover

6: **for** each pair of parents **do**

7:   Apply one-point crossover

8:   Apply uniform mutation

9: **end for**

10:   Combine initial population with offspring

11:   Evaluate the fitness of the combined population

12:   Sort the combined population by fitness

13:   Keep the top half of the sorted population

14:   Create a simplex from the best individuals (e.g., top 2)

15:   Perform Nelder-Mead steps on the simplex:

16:   - Reflection

17:   - Expansion

18:   - Contraction

19:   - Shrink

20:   Update the simplex

21:   Replace the worst individuals with the simplex’s best individuals

22:   Evaluate the fitness of the updated population

23: **end while**

24: From the final population, choose the best solution

25: Perform Nelder-Mead steps on the best solution

26: Find the optimal solution

## 4. The performance of GANMA

This study analyzes 15 benchmark test functions for simulation tests to fully investigate the feasibility as well as the effectiveness of GANMA. Windows is used as the experimental environment, while Python 3.11 is used as the programming environment. The 15 benchmark test functions (denoted as *f*_1_ to *f*_15_), cover different types. The unimodal functions *f*_1_ through *f*_4_ are included in the first kind. Multimodal functions *f*_5_ through *f*_9_ are included in the second category. Shifted unimodal and multimodal functions, *f*_10_ - *f*_15_, are included in the third category. Table 1 displays the expressions, ranges, and global minimum values of the 15 test functions. The function’s dimensions (n) are 10, 20, and 30, in that order. The GANMA method was compared to two well-known algorithms, the GA and NM algorithms. To ensure fair and reliable test results, the population size is uniformly set to 100, the number of iterations is set to 300, 400, and 600 for n = 10, 20, and 30, respectively, and the three algorithms are run 30 times independently for each of the three dimensions. The probability of crossover and mutation are ( *P _c_* = 0.8) and ( *P _m_* = 0.05) respectively, *γ* = 1.5, *β* = 0.5, and *η* = 0.5 have been chosen as the hybrid algorithm’s parameter values. The NM algorithm has a step size of 1.0 and a termination threshold of 1E-5.

**Table 1.** Benchmark test functions.

No	Function name	Formulation	Range	$f_{\min}$
$f_{1}$	Sphere	$\sum_{i=1}^{n}{x_{i}}^{2}$	[-100,100]	0
$f_{2}$	Rosenbrock	$\sum_{i=1}^{n-1}\left(100{\left(x_{i+1}-{x_{i}}^{2}\right)}^{2}+{\left(1-x_{i}\right)}^{2}\right)$	[-2,2]	0
$f_{3}$	Rotated high conditioned elliptic	$\sum_{i=1}^{n}{\left(10^{6}\right)}^{\frac{i-1}{n-1}}{x_{i}}^{2}$	[-100,100]	0
$f_{4}$	Ellipsoid	$\sum_{i=1}^{n}{\left(i.x_{i}\right)}^{2}$	[-100,100]	0
$f_{5}$	Ackley	$-20\exp\left(-0.2\sqrt{\frac{1}{n}\sum_{i=1}^{n}{x_{i}}^{2}}\right)-\exp\left(\;\frac{1}{n}\sum_{i=1}^{n}\cos\left(2\pi x_{i}\right)\right)+20+e$	[-30,30]	0
$f_{6}$	Griewank	$\sum_{i=1}^{n}{x_{i}}^{2}/4000-\prod_{i=1}^{n}\cos\left(\frac{x_{i}}{\sqrt{i}}\right)+1$	[-600,600]	0
$f_{7}$	Rastrigin	$10n+\sum_{i=1}^{n}\left({x_{i}}^{2}-10\cos\left(2\pi x_{i}\right)\right)$	[-5,5]	0
$f_{8}$	Schwefel	$418.9829n-\sum_{i=1}^{n}x_{i}\;\sin\left(\sqrt{\left|x_{i}\right|}\right)$	[-500,500]	0
$f_{9}$	Schwefel1.2	$\sum_{i=1}^{n}|x_{i}|$	[-5,5]	0
$f_{10}$	Shifted Sphere	$\sum_{i=1}^{n}{\left(x_{i}-{Ο}_{i}\right)}^{2}$	[-100,100]	0
$f_{11}$	Shifted Elliptic	$\sum_{i=1}^{n}{\left(10^{6}\right)}^{\frac{i-1}{n-1}}\;{\left(x_{i}-{Ο}_{i}\right)}^{2}$	[-100,100]	0
$f_{12}$	Shifted Rosenbrock	$\;\sum_{i=1}^{n-1}\left(100{\left(x_{i+1}-{x_{i}}^{2}\right)}^{2}+{\left(x_{i}-{Ο}_{i}\right)}^{2}\right)$	[-30,30]	0
$f_{13}$	Shifted Rastrigin	$\;\sum_{i=1}^{n}\left[{\left(x_{i}-{Ο}_{i}\right)}^{2}-10\cos\left(2\pi \left(x_{i}-{Ο}_{i}\right)\right)\right]$	[-5,5]	0
$f_{14}$	Shifted Griewank	$\frac{1}{4000}\sum_{i=1}^{n}{\left(x_{i}-{Ο}_{i}\right)}^{2}-\prod_{i=1}^{n}\cos\left(\frac{x_{i}-{Ο}_{i}}{\sqrt{i}}\right)+1$	[-600,600]	0
$f_{15}$	Shifted Ackley	$-20\exp\left(-0.2\sqrt{\frac{1}{n}\sum_{i=1}^{n}{\left(x_{i}-{Ο}_{i}\right)}^{2}}\right)-\exp\left(\frac{1}{n}\sum_{i=1}^{n}\cos\left(2\pi \left(x_{i}-{Ο}_{i}\right)\right)\right)+20+e$	[-30,30]	0

Ο = ( ${Ο}_{1},{Ο}_{2},..,{Ο}_{n}$ ) is the shift vector. n = dimension.

Table 2 demonstrates how the performance of the GANMA, GA, and NM algorithms for dimensions (n) 10, 20, and 30 have been evaluated by comparing the mean value (Mean), standard deviation (Std), and best value (Best) of the final solutions for each benchmark function throughout 30 trials. The algorithm achieves the best optimization performance with the least standard deviation, optimal value, and average value closer to the theoretical ideal value. Any value less than 10 ^*−*6^ in terms of mean, standard deviation, and best value will be regarded as zero. The ideal experimental outcomes are truncated.

**Table 2.** For n = 10, 20, and 30, the best, mean, and standard deviation of the GANMA, GA, and NM solutions.

Fun	Method	n = 10 Best	Mean	Std	n = 20 Best	Mean	Std	n = 30 Best	Mean	Std
	GANMA	3.92E-278	1.01E-235	0.00E+00	4.62E-52	1.10E-45	3.30E-45	1.09E-20	4.10E-17	1.10E-16
*f*_1_	GA	2.96E-01	8.90E-01	1.39E-02	1.24E+00	3.03E+00	1.45E+00	1.5E+02	2.8E+02	1.0E+02
	NM	3.96E-183	6.63E-166	0.00E+00	5.39E-37	2.03E-32	5.48E-32	2.51E-18	7.47E-13	1.11E-12
	GANMA	5.22E-29	1.44E-28	7.60E-29	3.69E-27	6.94E-26	9.75E-26	6.49E-18	1.80E-14	1.85E-14
*f*_2_	GA	5.67E+00	6.90E+00	8.78E-01	1.64E+01	1.74E+01	4.81E-01	2.62E+01	4.07E+01	1.63E+01
	NM	5.91E-29	2.38E-27	2.31E-27	1.89E-26	2.30E-25	3.20E-25	9.10E-16	1.43E-11	2.60E-11
	GANMA	7.88E-243	4.78E-232	0.00E+00	6.93E-49	3.97E-45	1.12E-44	1.58E-16	7.55E-14	1.39E-13
*f*_3_	GA	1.36E+03	5.08E+04	1.03E+04	9.85E+03	5.22E+04	2.93E+04	7.98E+03	2.41E+04	1.00E+04
	NM	9.32E-186	4.62E-165	0.00E+00	1.16E+01	1.44E+04	1.14E+04	8.87E+04	9.68E+05	3.88E+05
	GANMA	7.43E-250	6.45E-235	0.00E+00	2.09E-50	5.42E-44	1.57E-43	1.44E-16	3.78E-15	4.98E-15
*f*_4_	GA	8.60E-02	5.56E-01	3.81E-01	1.11E+00	7.66E+00	6.05E+00	1.38E+04	2.15E+04	6.00E+03
	NM	6.30E-184	4.58E-171	0.00E+00	4.59E-39	1.00E-32	2.53E-32	2.31E-15	3.89E-11	1.05E-10
	GANMA	2.88E-14	3.18E-13	1.62E-13	4.01E-13	1.11E-11	1.45E-11	7.09E-10	4.39E-11	1.29E-11
*f*_5_	GA	7.17E-02	9.74E-01	6.37E-01	4.40E-01	8.93E-01	2.78E-01	2.23E+00	3.21E+00	5.99E-01
	NM	1.86E+01	1.93E+01	3.00E-01	1.90E+01	1.94E+01	2.17E-01	1.84E+01	1.89E+01	2.53E-01
	GANMA	7.13E-02	1.46E-01	5.22E-02	1.66E-01	2.52E-01	2.17E-01	9.41E-01	1.66E+00	5.25E-01
*f*_6_	GA	1.86E-01	3.56E-01	1.11E-01	1.00E+00	1.02E+00	1.39E-02	2.47E+00	3.53E+00	9.43E-01
	NM	1.42E+00	2.57E+01	1.34E+01	7.39E-02	5.71E+00	7.57E+00	9.09E-13	2.02E+00	1.65E+00
	GANMA	1.96E-05	2.00E-04	2.00E-03	4.00E-04	8.90E-03	7.70E-03	1.19E+00	2.84E+00	1.16E+00
*f*_7_	GA	6.29E-05	2.20E-02	3.40E-02	1.00E-03	1.46E-02	1.56E-02	2.47E+00	3.58E+00	1.80E+00
	NM	8.30E+01	1.06E+02	1.74E+01	1.19E+02	1.71E+02	5.18E+01	1.88E+02	2.89E+02	5.49E+01
	GANMA	-1.97E+01	-1.57E+01	7.89E+00	-3.94E+01	-1.18E+01	1.57E+01	-9.86E+01	-4.73E+01	2.95E+01
*f*_8_	GA	3.45E-02	7.88E-02	4.12E-02	6.72E+00	8.25E+00	1.10E+00	1.09E+02	1.54E+02	3.0E+01
	NM	6.31E+02	1.37E+04	3.81E+02	2.32E+03	2.84E+03	3.82E+02	4.06E+03	5.23E+03	1.04E+03
	GANMA	5.11E-09	4.88E-06	4.95E-06	4.77E-06	1.56E-05	8.25E-06	8.83E-06	2.30E-05	1.05E-05
*f*_9_	GA	8.00E-05	8.50E-02	7.60E-02	2.56E-01	5.47E-01	1.80E-01	2.38E+00	3.89E+00	9.46E-01
	NM	6.98E-01	1.21E+00	4.94E-01	1.87E+00	6.43E+00	5.55E+00	2.25E+00	7.34E+00	4.84E+00
	GANMA	2.46E-31	8.02E-31	5.62E-31	1.01E-29	4.13E-29	2.00E-29	9.12E-16	1.05E-16	3.02E-16
*f*_10_	GA	1.18E-05	3.14E-02	6.74E-02	9.86E+01	1.37E+02	3.49E+01	1.89E+03	3.65E+03	1.11E+03
	NM	1.78E-30	7.60E-30	6.09E-30	5.08E-29	1.96E-28	1.51E-28	1.84E-15	1.13E-09	3.00E-09
	GANMA	2.90E-26	5.12E-25	5.85E-24	3.16E-24	7.45E-24	3.25E-24	1.46E-15	8.71E-14	1.69E-13
*f*_11_	GA	1.01E+00	3.49E+02	5.78E+02	1.00E+05	1.62E+05	8.76E+03	5.63E+05	1.11E+06	3.41E+06
	NM	3.68E-26	7.21E-25	1.13E-24	2.93E+03	1.98E+04	1.55E+04	1.29E+04	2.49E+05	1.51E+05
	GANMA	8.13E-29	6.12E-28	4.43E-28	1.59E-26	1.45E-25	1.92E-25	1.14E-18	2.20E-15	4.29E-15
*f*_12_	GA	9.03E+00	4.87E+01	3.27E+01	1.46E+02	2.11E+02	7.77E+01	7.88E+03	2.52E+04	1.12E+04
	NM	1.15E-27	1.59E+00	1.95E+00	3.40E-25	7.97E-01	1.59E+00	2.94E-14	3.18E+00	1.59E+00
	GANMA	1.44E-02	2.13E-02	1.02E-02	2.82E+00	3.74E+00	1.71E+00	9.01E+00	1.05E+01	1.43E+00
*f*_13_	GA	4.60E-02	1.49E-01	1.19E-01	8.14E+00	9.42E+00	1.12E+00	1.58E+01	1.98E+01	3.71E+00
	NM	6.40E+01	8.76E+01	2.60E+01	1.70E+02	2.07E+02	2.36E+01	2.08E+02	3.03E+02	5.42E+01
	GANMA	1.42E-02	4.42E-02	2.85E-02	8.11E-02	1.21E-01	7.64E-02	1.21E-13	4.90E-02	4.10E-02
*f*_14_	GA	2.10E-01	4.81E-01	1.79E-01	1.83E+00	2.32E+00	2.97E-01	2.73E+00	3.75E+00	5.10E-01
	NM	5.36E+00	3.23E+01	4.09E+01	2.40E-01	1.34E+01	9.05E+00	7.30E-02	7.99E-01	9.70E-01
	GANMA	5.01E-14	1.98E-12	3.10E-12	3.25E-12	1.43E+00	1.77E+01	2.22E+00	4.27E+00	3.10E+00
*f*_15_	GA	1.10E-01	4.17E-01	1.20E-01	2.91E+00	3.85E+00	6.97E-01	9.29E+00	1.04E+01	1.23E+00
	NM	1.85E+01	1.89E+01	2.95E-01	1.89E+01	1.93E+01	2.33E-01	1.92E+01	1.93E+01	1.31E-01

### 4.1 Experimental results and analysis

The statistical results of GANMA’s performance on 15 benchmark functions with dimensions (n) of 10, 20, and 30 are shown in Table 2. It also contains the final solutions’ best (Best), mean (Mean), and standard deviation (Std) across a 30-run period for each benchmark function. All benchmark functions for unimodal functions ( *f*_1_ - *f*_4_) have been solved in all three dimensions (10, 20, and 30). For the multimodal functions ( *f*_5_ – *f*_9_), the solutions for *f*_5_ and *f*_9_ occur in 10, 20, and 30 dimensions, whereas the solutions for *f*_6_ and *f*_7_ in 10 and 20 dimensions are almost optimal. The standard deviation range is 1 *.*62 *E* − 13 ∼ 7 *.*89 *E* + 00, 1 *.*45 *E* − 11 ∼ 1 *.*57 *E* + 01, and 1 *.*29 *E* − 11 ∼ 2 *.*95 *E* + 01, respectively, while the mean value’s variations range in the 10, 20, and 30 dimensions is 3 *.*18 *E* − 13 ∼ 1 *.*46 *E* − 01, 1 *.*11 *E* − 11 ∼ 2 *.*52 *E* − 01, and 4 *.*39 *E* − 11 ∼ 2 *.*84 *E* + 00.

Six shifted test functions have been chosen for this study to validate the performance of GANMA: three shifted multimodal test functions, denoted as *f*_13_ to *f*_15_; and three shifted unimodal test functions, denoted as *f*_10_ to *f*_12_, Sphere, Elliptic, and Rosenbrock. On functions *f*_10_, *f*_11_, *f*_12_ (in 10, 20, and 30), and *f*_15_ (in 10), GANMA achieved optimum solutions; on functions *f*_13_ (in 10) and *f*_14_ (in 10, 20, and 30), the solutions are nearly optimal. Even while GA is outperformed by the solutions of *f*_13_ and *f*_15_ (in 20 and 30) in GANMA, the solutions are still far from the optimal ones. Furthermore, the Std that GANMA found on five test functions is not too high, suggesting that GANMA’s performance on shifted test functions is steady.

Therefore, for all unimodal functions (in 10, 20, and 30 dimensions), GANMA can obtain the global optimum. GANMA can identify outcomes with negligible deviations from the global optimal value for multimodal functions. Except for *f*_5_ and *f*_9_ in dimensions 10, 20, and 30, the results of *f*_6_, and *f*_7_ in dimensions 10 and 20 are quite near to the optimal value. The outcomes produced by GANMA algorithms for shifted unimodal and multimodal functions are optimal or extremely near-optimal in all functions for all three dimensions, except *f*_13_ and *f*_15_ (in 20 and 30). The benefits of the GANMA algorithm include excellent robustness, high convergence accuracy, and steady performance in all scenarios, whether they involve multimodal functions, unimodal functions, or shifting unimodal and multimodal functions. This is shown in Table 2 under the various numbers of iterations for the corresponding dimensions, which are 300, 400, and 600 for the dimensions 10, 20, and 30, respectively.

To help further investigate the evolutionary behavior of various methods, the convergence curves of GANMA and GA for a few chosen benchmark functions are displayed in Figure 2, Figure 3, and Figure 4 for dimensions (n) = 10, 20, and 30, respectively. These graphs demonstrate the convergence behavior of methods that can help to analyze the evolutionary behavior of various algorithms. The y- and x-axes, respectively, represent the values of the fitness function and the number of iterations. The blue solid line shows the genetic algorithm (GA), while the suggested method GANMA is shown by the solid orange line.

**Figure 1.**
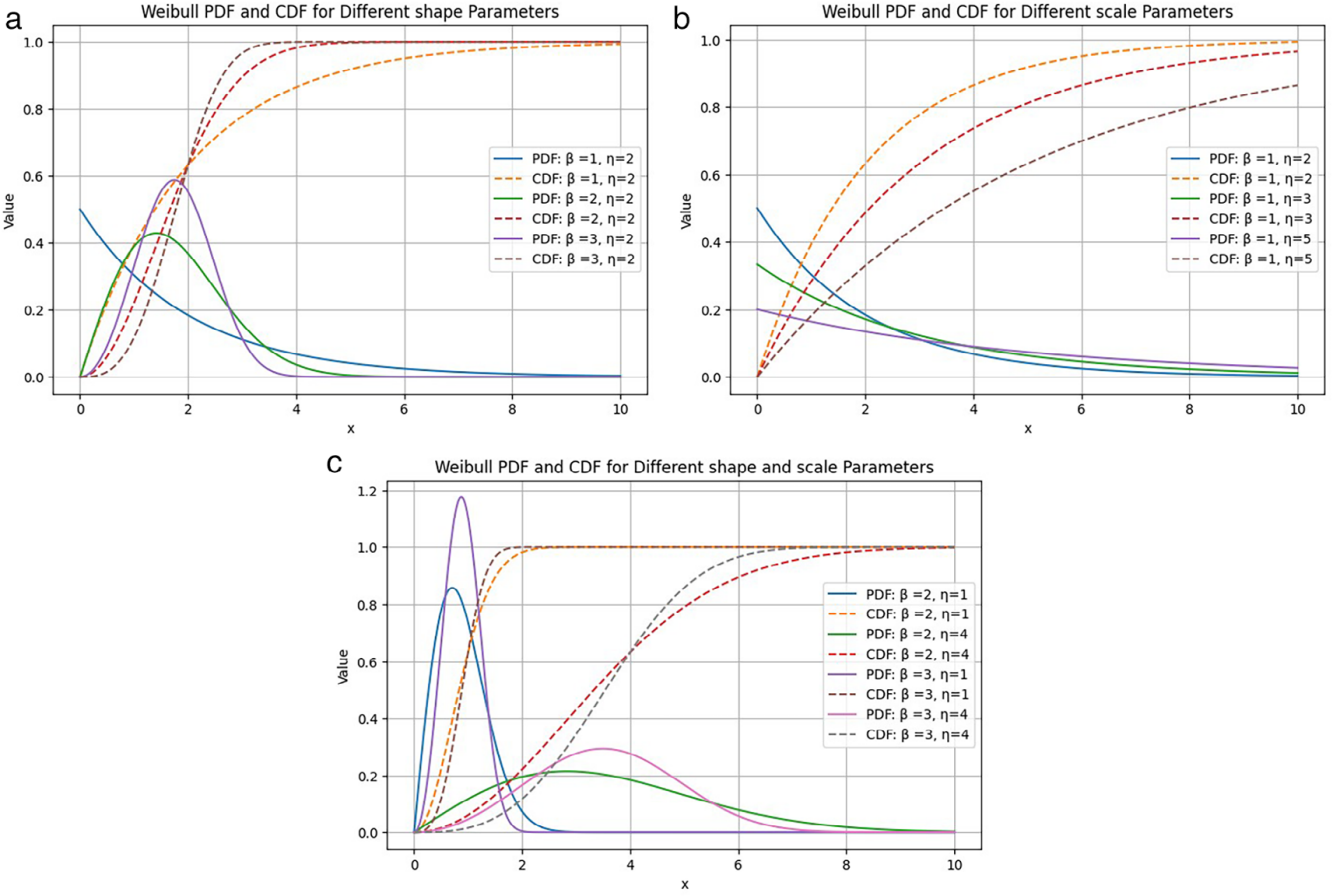
Different values of (a) shape parameter *β* and (b) scale parameter *η* are plotted in Weibull PDF (solid line) and CDF (dashed line) plots.

**Figure 2.**
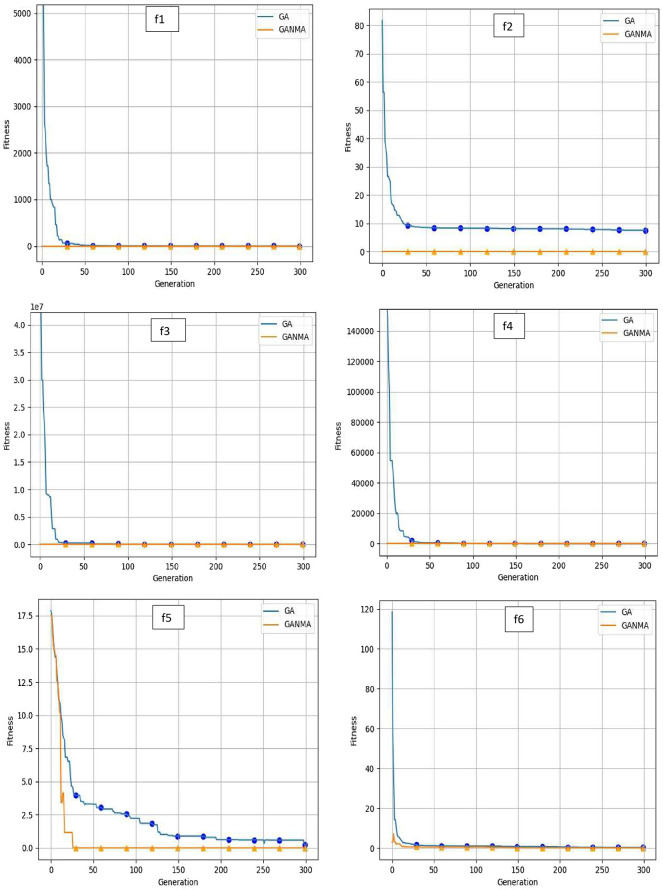

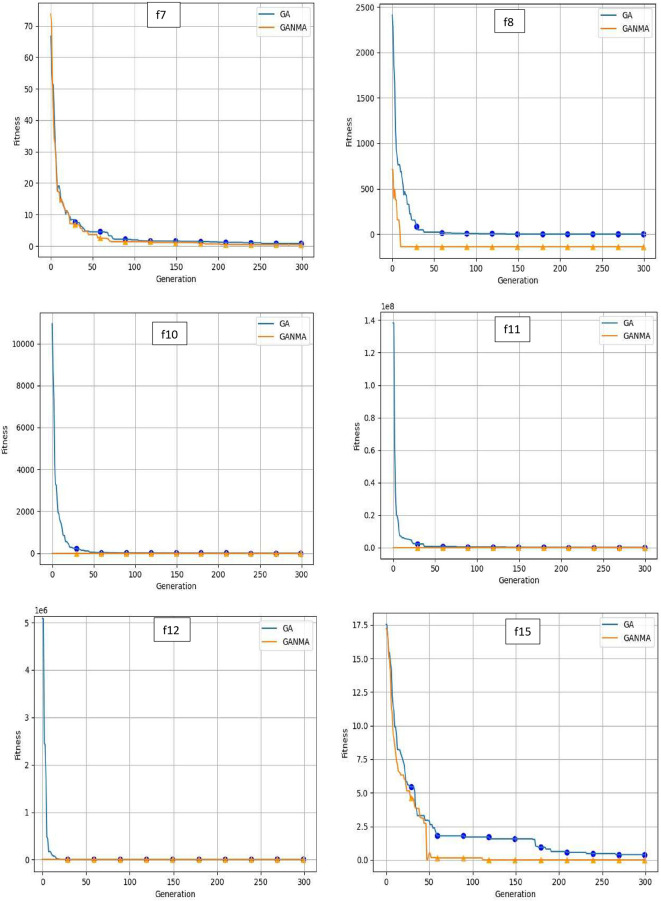
Convergence graphs of functions for n = 10.

**Figure 3.**
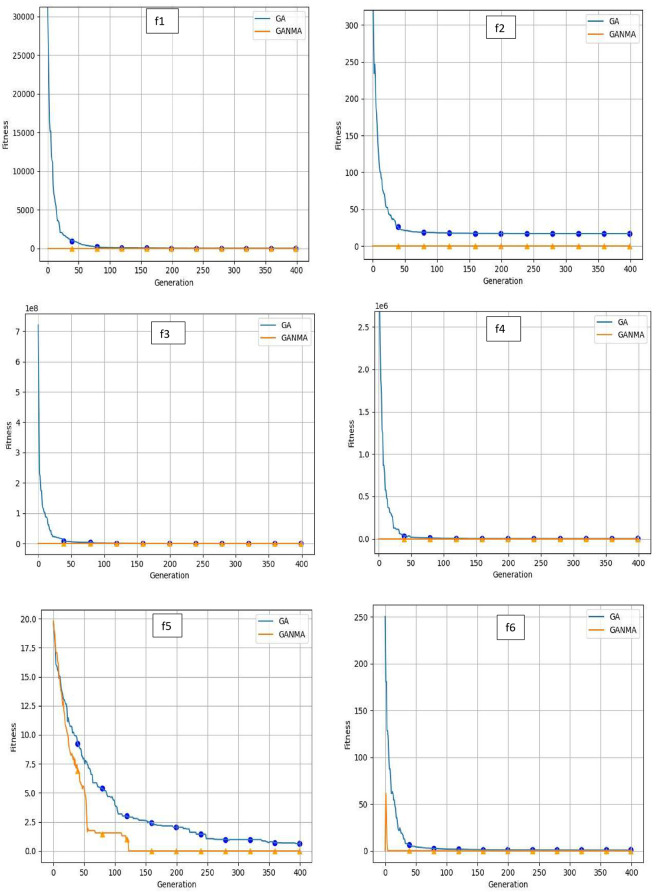

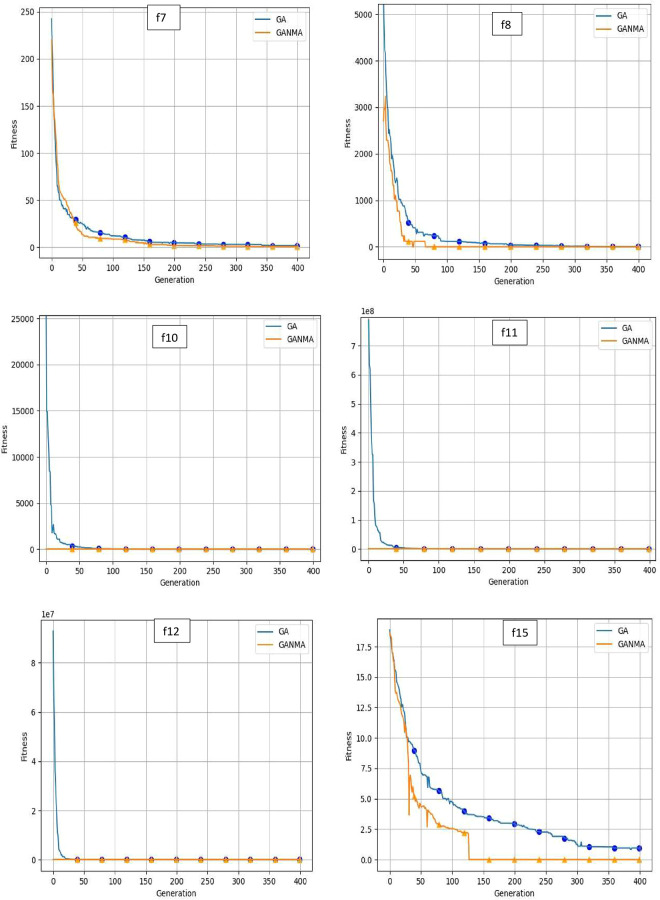
Convergence graphs of functions for n = 20.

**Figure 4.**
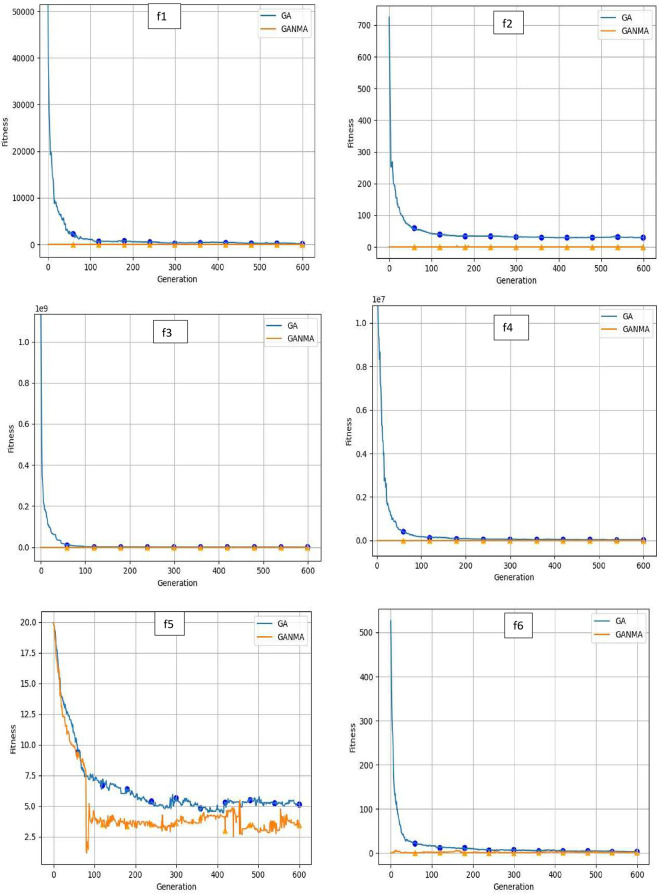

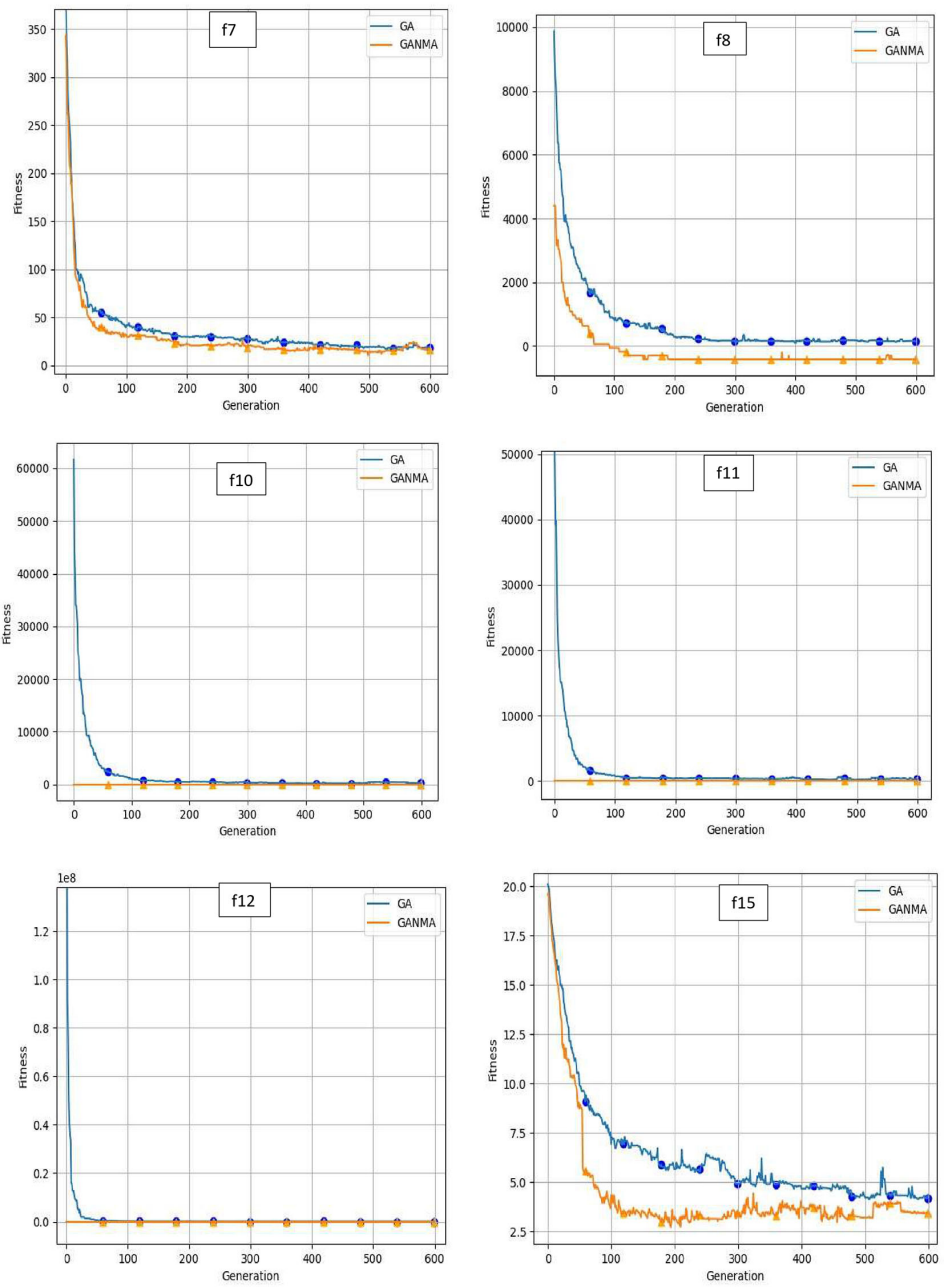
Convergence graphs of functions for n = 30.

Until the ideal solution is discovered, GA shows a decreasing trend for unimodal functions like *f*_1_, *f*_2_, *f*_3_, and *f*_4_. In contrast, GANMA presents a straight line for all three dimensions (n = 10, 20, and 30). Similar to this, for multimodal functions other than *f*_5_ and *f*_7_ (in 30), there is a greater similarity between the global optimum solution and the GANMA optimal solution in *f*_5_, *f*_7_ (in 10, and 20), and *f*_6_, *f*_8_ (in 10, 20, and 30). As a result, of these two algorithms, the lowest optimum solution and the fastest rate of convergence are found through GANMA. The curves for shifted functions, except *f*_15_ (in 20 and 30), demonstrate how well the proposed method was able to obtain the ideal solution for other functions like *f*_10_, *f*_11_, and *f*_12_ (in 10, 20, and 30). Out of these two methods, GANMA yields the lowest optimum solution and has the fastest convergence rate than GA’s in both the multimodal and shifted functions.

Examining the convergence curve and experimental results, it can be shown that GANMA generally performs exceptionally well on the 15 test functions, with a proportion of correct convergence to the global optimal solution that approaches 90%. When compared to GA and the NM algorithm, GANMA outperforms both in terms of exploration and exploitation. As a result, GANMA can meet the requirements for addressing diverse optimization issues, increasing its competitiveness.

## 5. Application of proposed algorithm (GANMA) for Weibull-Parameter Estimation

The Weibull distribution is a probability distribution that is often used in reliability and survival research. Weibull et al. ^13^ had shown that the Weibull distribution fit many different datasets and offered satisfactory results, even for small samples. The Weibull distribution, known for its flexibility in modeling various failure and survival scenarios, is defined by two parameters: the shape ( *β*) and scale ( *η*) parameters. In some cases, a location ( *α*) parameter is added to create a three-parameter Weibull distribution, allowing for greater flexibility in fitting data with location shifts. The three-parameter probability density function (pdf) will have only two parameters ^14^ when the location parameter ( *α*) is equal to zero. Because no failure may occur before or after the time is zero, the two Weibull parameters are frequently utilized in failure analysis. ^15^

Weibull parameter estimation employs a variety of methods. Method of Moments (MOM), the maximum likelihood (ML) approach, and modified maximum likelihood (MML) methods were all used by Seguro and Lambert. ^16^ They discovered that the time series data sets are more suited for the ML approach. They advised utilizing the MML technique for data sets that were formatted as frequency distributions. The least squares approach, the ML method, and the MML method were contrasted by Akgül et al. ^17^ ML was shown to be the most effective approach overall, but they also noted that MML and ML are equally effective for big data sets, despite MML’s lower computational complexity. The ML technique was used in the studies of Kollu et al. ^18^ and Akpnar and Akpnar ^19^ to estimate the Weibull parameters. Teimouri et al. ^20^ investigated the MoM using their proposed L-moment estimator, the ML approach, the logarithmic moment method, and the percentile method. They discovered that the ML method and their suggested approach are the most effective estimators. The power density approach was proposed by Akdağ and Dinler. ^12^ They concluded that it outperformed popular techniques like MoM and ML techniques. After evaluating five different methods for approximating the Weibull distribution, Saleh et al. ^21^ recommended the mean wind speed methodology and the ML method. Azad and colleagues ^22^ discovered that the MoM and ML techniques were more effective than other approaches.

Considering the Weibull distribution has a nonlinear log-likelihood function and is compatible with numerical optimization techniques like Newton-Raphson (NR) and Nelder-Mead (NM), previous studies have often used MLE approaches for parameterizing the Weibull distribution. However, the effectiveness of these iterative methods heavily relies on the initial value chosen. In a departure from traditional approaches, this study employs Genetic Algorithms (GAs) as a heuristic search method, considering a set of solutions within the search space rather than individual points, to address the initial value problem in Weibull parameter Maximum Likelihood estimation. ^23^^,^^24^ GAs have been successfully applied in various optimization contexts, ranging from optimizing mixing parameters for high-performance concrete to signal control optimization. Parameterization of distributions such as the skew-normal distribution, ^25^ nonlinear regression, ^26^ and negative binomial gamma mixed distribution ^27^ have all been applied previously. Notably, Thomas et al. ^28^ pioneered the use of GA for Weibull distribution parameter estimation in the context of break-down periods of insulating fluid data, achieving performance comparable to traditional methods based on maximizing the log-likelihood function.

### 5.1 Weibull distribution

A versatile continuous probability distribution, the Weibull distribution is frequently used in survival analysis and reliability engineering. It is characterized by its ability to model the distribution of time until an event occurs. Named after Wallodi Weibull, who described it in the 1950s, the distribution is flexible and can take different shapes depending on its parameters. The shape parameter affects the structure of the Weibull distribution curve resulting in whether the distribution appears to be a Rayleigh distribution ( *β* = 2), an exponential distribution ( *β* = 1), or another shape. The scale parameter determines the distribution’s scale or size. Together, these factors enable the Weibull distribution to simulate a wide range of events with varying shapes and sizes.

The following is the Weibull two-parameter distribution’s probability density function (PDF):

$$f\left(x;\beta ,\eta \right)=\{\begin{array}{ll} \frac{\beta }{\eta }{\left(\frac{x}{\eta }\right)}^{\beta -1}e^{-{\left(x/\eta \right)}^{\beta }}, & x\geq 0\\ 0, & x<0 \end{array}$$ (1)

where:

• *x* is the random variable,
•
β is the shape parameter,
•
η is the scale parameter.

The following represents the Weibull distribution’s cumulative distribution function (or CDF):

$$F\left(x;\beta ,\eta \right)=\left\{ \begin{array}{ll} 1-e^{-{\left(x/\eta \right)}^{\beta }}, & x\geq 0\\ 0, & x<0 \end{array}\right.$$ (2)

Probability density and cumulative distribution plots for some different parameter values are given in Figure 1.

Two-Parameter Weibull is Commonly applied in reliability engineering for modeling time until the failure of components. Whereas, Three-Parameter Weibull is Useful when considering scenarios where the event initiation may not be at zero, such as analyzing the time until an event occurs after a certain threshold.

## 6. Methods for estimating parameters

Estimating the parameters of the Weibull distribution poses a significant challenge due to the intricacies involved in utilizing sample data for accurate estimation. Parameter estimation involves the process of determining the distribution’s parameters using available sample data, aiming to derive optimal values that provide meaningful insights into the underlying data. Making incorrect parameter choices can lead to misleading results, underscoring the importance of analyzing and selecting appropriate estimation techniques for accurate modeling. Therefore, a thorough evaluation of estimation methods is essential to determine the most suitable approach for a given dataset and analysis context.

### 6.1 Maximum Likelihood Estimation (MLE)

The statistical method known as Maximum Likelihood Estimation (MLE) is used to estimate Weibull parameters by maximizing the likelihood function, which determines how well the distribution fits the observed data. MLE is known for its efficiency, but its optimization can be complex due to non-linear equations and numerical stability issues. The PDF of the Weibull distribution is given by Equation (1). Given a sample x _1_, x _2_, … x _n_ from a Weibull distribution, the likelihood function is given by:

$$L\left(\beta ,\eta \right)=\prod_{i=1}^{n}\;f\left(x_{i};\beta ,\eta \right)$$ (3)

where, *f* ( *x*; *β, η*) is the probability density function of the Weibull distribution.

The Weibull distribution’s log-likelihood function is as follows:

$$\mathrm{LnL}\left(\beta ,\eta \right)=\sum_{i=1}^{n}\;\left[\ln\left(\frac{\beta }{\eta }\right)+\left(\beta -1\right)\ln\left(\frac{x_{i}}{\eta }\right)-{\left(\frac{x_{i}}{\eta }\right)}^{\beta }\right]$$ (4)

$$\mathrm{LnL}\left(\beta ,\eta \right)=\mathrm{nln\beta}-\text{nβln}\eta +\left(\beta -1\right)\sum_{i=1}^{n}\;\ln x_{i}-\eta^{-\beta }\sum_{i=1}^{n}\;x_{i}\beta$$ (5)

The log-likelihood function is differentiated with respect to *β* and *η*, the derivatives are set to zero, and the resultant system of equations is solved to get the MLE.

$$\frac{\partial L}{\partial \eta }=-\frac{\mathrm{n\beta}}{\eta }+\frac{\beta }{\eta^{\beta }+1}\sum_{i=1}^{n}\;x_{i}^{\beta }=0$$ (6)

$$\frac{\partial L}{\partial \beta }=\frac{n}{\beta }-\mathrm{nln\eta}-\frac{\sum_{i=1}^{n}\;x_{i}\beta -\ln\eta \sum_{i=1}^{n}\;x_{i}\beta }{\eta^{\beta }}+\sum_{i=1}^{n}\;\ln x_{i}=0$$ (7)

By eliminating *α* from the above equations and simplifying the equations we get,

$$\hat{\eta }={\left(\frac{1}{n}\sum_{i=1}^{n}\;x_{i}\beta \right)}^{\frac{1}{\beta }}$$ (8)

$$\frac{1}{\beta }-\frac{\sum_{i=1}^{n}\;x_{i}^{\beta }\ln x_{i}}{\sum_{i=1}^{n}\;x_{i}^{\beta }}+\frac{1}{n}\sum_{i=1}^{n}\;\ln x_{i}=0$$ (9)

Eqn. (8) may be used to calculate the estimate $\hat{\eta }$ . However, because of Eqn. (9) did not give an analytical solution, the estimate $\hat{\beta }$ must be calculated numerically. This is possible by using the optimization strategy. The Nelder-Mead, Newton Rapson, simulated annealing, or GA algorithms can all be used to solve the nonlinear function that the ML estimator of the shape parameter *β* contains. In this study, the suggested method, GA, and NM were all used to optimize the log-likelihood function. Nelder-Mead is a powerful algorithm that converges quickly, but its performance is dependent on the initial guess. As a result, we took into account the GA while maximizing the Weibull distribution’s loglikelihood function. Eqn. (5) is considered a fitness function for GA and NM methods.

Below the proposed method on MLE of Weibull Distribution has been described briefly.

***6.1.1 Proposed method***

***(Genetic and Nelder -Mead Algorithm (GANMA))***

To improve the precision and reliability of parameter estimation, we proposed a hybrid approach GANMA that integrates the GA and the NM method with MLE for two-parameter Weibull distributions. The GA aids in exploring the parameter space globally, generating diverse candidate solutions, while the NM fine-tunes these solutions through local search, aiming for optimal parameter estimates. To the best of our knowledge, this is the first instance where the GANMA is being utilized to estimate the Weibull distribution’s parameters.

The steps of the proposed method in this study are summarized as follows:

**Step 1:** Problem Formulation - We aim to find the MLE parameters *β* (shape) and *η* (scale) for a Weibull distribution.

**Step 2:** Genetic Algorithm (GA) Phase -

• Generate an initial population (P) of possible solutions. For the Weibull distribution, each solution indicates a collection of parameters ( *β*, *η*).
• Define the fitness function f( *β*, *η*) that measures the goodness of fit between the observed data and the Weibull distribution with the given parameters. A suitable fitness function could be the log-likelihood shown in Equation 5.
• Select individuals within the population according to their fitness by using a selection process (tournament selection). Higher fitness levels increase the probability of selection.
• Apply crossover operations (one-point crossover) to pairs of selected individuals to create new candidate solutions.
• Introduce small random changes (mutations) to the parameters of some individuals to add diversity to the population.

**Step 3:** Nelder-Mead Algorithm (NM) Phase -

• Take the best individual from the final population of the GA as an initial guess for the parameters ( *β*1, *η*1).
• Define the log-likelihood function L( *β*, *η*) for the Weibull distribution shown in equation 5.
• To minimize the log-likelihood function and improve the parameter estimations (i.e., reflection, expansion, contraction, and shrinkage), apply the Nelder-Mead method.
• Repeat the iterations until convergence criteria are met (e.g., small changes in parameters or a maximum number of iterations).

**Step 4:** Repeat the selection, crossover, and mutation steps for several generations until convergence is met (i.e. end of GA phase).

**Step 5:** Apply the NM method to the best GA solution once again after the GA phase.

**Step 6:** Result - The final parameters ( $\hat{\beta }$ , $\hat{\eta }$ ) obtained from the Nelder-Mead optimization represent the Maximum Likelihood Estimates (MLE) for the Weibull distribution.

## 7. Monte Carlo simulations

The two-parameter Weibull distribution parameter estimation methods were investigated using a Monte Carlo simulation. The scale parameter was set to 1, while the other shape parameters were set to 0.5, 1, 3, and 6. The simulation has been repeated 1000 times for sample sizes of 20, 100, and 500 respectively. With a population size of 100, the GA and GANMA have corresponding crossover and mutation rates of 0.1 and 0.8. The parameters that are used to compare the goodness-of-fit of different parameter estimating methods are mean absolute error (MAE) and bias. For the parameters *β* (shape) and *η* (scale), MAE and bias are computed using the formula provided by:

(For shape parameter)

$$\text{MAE}\left(\hat{\beta }\right)=\frac{1}{n}\sum_{i=1}^{n}\left|\hat{\beta_{i}}-\beta_{i}\right|$$ (10)

$$\text{bias}\left(\hat{\beta }\right)=\frac{1}{n}\sum_{i=1}^{n}\left(\hat{\beta_{i}}-\beta_{i}\right)$$ (11)

(For scale parameter)

$$\mathrm{MAE}\left(\hat{\eta }\right)=\frac{1}{n}\sum_{i=1}^{n}\left|\hat{\eta_{i}}-\eta_{i}\right|$$ (12)

$$\text{bias}\left(\hat{\eta }\right)=\frac{1}{n}\sum_{i=1}^{n}\left(\hat{\eta_{i}}-\eta_{i}\right)$$ (13)

Greater efficiency is implied by lower absolute values of the bias and MAE. For various data sizes and shape parameters, Tables 3- 5 displays the parameter estimates, bias, and MAE for each parameter estimation method. The results of the simulation demonstrate that the GANMA approach performed better than NM and GA when estimating shape and scale parameters based on MAE and bias criteria. The best results are highlighted in bold.

**Table 3.** Estimations of parameters, MAE, and bias values for several simulation scenarios with n = 20 of a two-parameter distribution for *β* = 0.5, 1, 3, and 6.

n	*β*	Method	$\hat{\beta }$			$\hat{\eta }$		
			Mean	MAE	Bias	Mean	MAE	Bias
		NM	0.62394	0.12395	0.12393	2.19877	1.19876	1.19871
	0.5	GA	0.60901	0.13641	0.10901	1.76523	0.81895	0.76523
		GANMA	0.60514	**0.10514**	**0.10514**	1.50806	**0.50806**	**0.50806**
	1	NM	1.21029	0.21028	0.21029	1.22803	0.22810	0.22808
		GA	1.23962	0.29000	0.23961	1.38053	0.41456	0.38053
		GANMA	1.21029	**0.21029**	**0.21009**	1.22803	**0.22803**	**0.22805**
20	3	NM	2.04136	0.95863	-0.95863	1.95863	0.14136	-0.14163
		GA	3.63089	0.70843	**0.41377**	1.07488	0.09595	0.074881
		GANMA	3.41374	**0.63089**	0.63088	1.07087	**0.07087**	**0.070870**
		NM	3.08815	2.91184	-2.91146	1.91184	0.08815	-0.08813
	6	GA	5.3846	1.0844	**-0.61433**	1.05115	0.07196	0.05115
		GANMA	6.26177	**1.06178**	1.26178	1.03482	**0.03482**	**0.034828**

**Table 4.** Estimations of parameters, MAE, and bias values for several simulation scenarios with n = 100 of a two-parameter distribution for *β* = 0.5, 1, 3, and 6.

n	*β*	Method	$\hat{\beta }$			$\hat{\eta }$		
			Mean	MAE	Bias	Mean	MAE	Bias
		NM	0.50000	2.66453	2.88657	1.24999	0.24999	0.24989
	0.5	GA	0.58267	0.08833	-0.04039	1.74933	0.92293	0.67225
		GANMA	0.49477	**0.01522**	**-0.0152**	0.08211	**0.17880**	**-0.17868**
	1	NM	1.00000	1.7763	1.7322	1.00000	**0.0000**	**0.0000**
		GA	0.76607	0.17435	**-0.01683**	0.83393	0.28349	0.11227
		GANMA	0.97954	**0.03045**	-0.03045	0.90620	0.09379	-0.09386
100	3	NM	2.10606	0.89393	-0.89396	0.89393	0.10606	-0.10606
		GA	2.90103	0.45456	**-0.01648**	1.07781	0.09780	-0.08386
		GANMA	2.91863	**0.09136**	-0.09135	0.96769	**0.03230**	**-0.03231**
		NM	2.12499	3.87500	-3.87501	0.87500	0.12499	-0.12497
	6	GA	4.48285	1.10891	-0.93864	0.98534	0.05621	-0.01811
		GANMA	5.51726	**0.18273**	**-0.18273**1	0.98371	**0.01628**	**-0.01625**

**Table 5.** Estimations of parameters, MAE, and bias values for several simulation scenarios with n = 500 of a two-parameter distribution for *β* = 0.5, 1, 3, and 6.

n	*β*	Method	$\hat{\beta }$			$\hat{\eta }$		
			Mean	MAE	Bias	Mean	MAE	Bias
		NM	0.50392	0.00392	0.00391	1.24934	0.249345	0.249344
	0.5	GA	0.5055	0.0863	0.0055	1.8336	0.9586	0.8336
		GANMA	0.49826	**0.00677**	**-0.00671**	1.0054	**0.00545**	**-0.00544**
		NM	1.000	**0.000**	**0.000**	1.000	**0.000**	**0.000**
	1	GA	0.9676	0.1473	-0.0323	1.1998	0.29807	0.19986
500		GANM	0.98653	0.0134	-0.0133	1.00272	0.00272	-0.00277
		NM	2.0825	0.91747	-0.91746	0.91747	0.08252	-0.08251
	3	GA	3.2000	0.52541	0.2000	1.0404	0.08547	0.0404
		GANMA	2.95959	**0.04040**	**-0.04040**	1.00090	**0.00090**	**-0.00080**
		NM	2.11664	3.88335	-3.88334	0.883357	0.11664	-0.11667
	6	GA	5.2108	1.0329	-0.7891	1.0060	0.06851	0.00605
		GANMA	5.6191	**0.08080**	**-0.08081**	1.0004	**0.00045**	**-0.00043**

### 7.1 Result analysis

**Figure 5.**
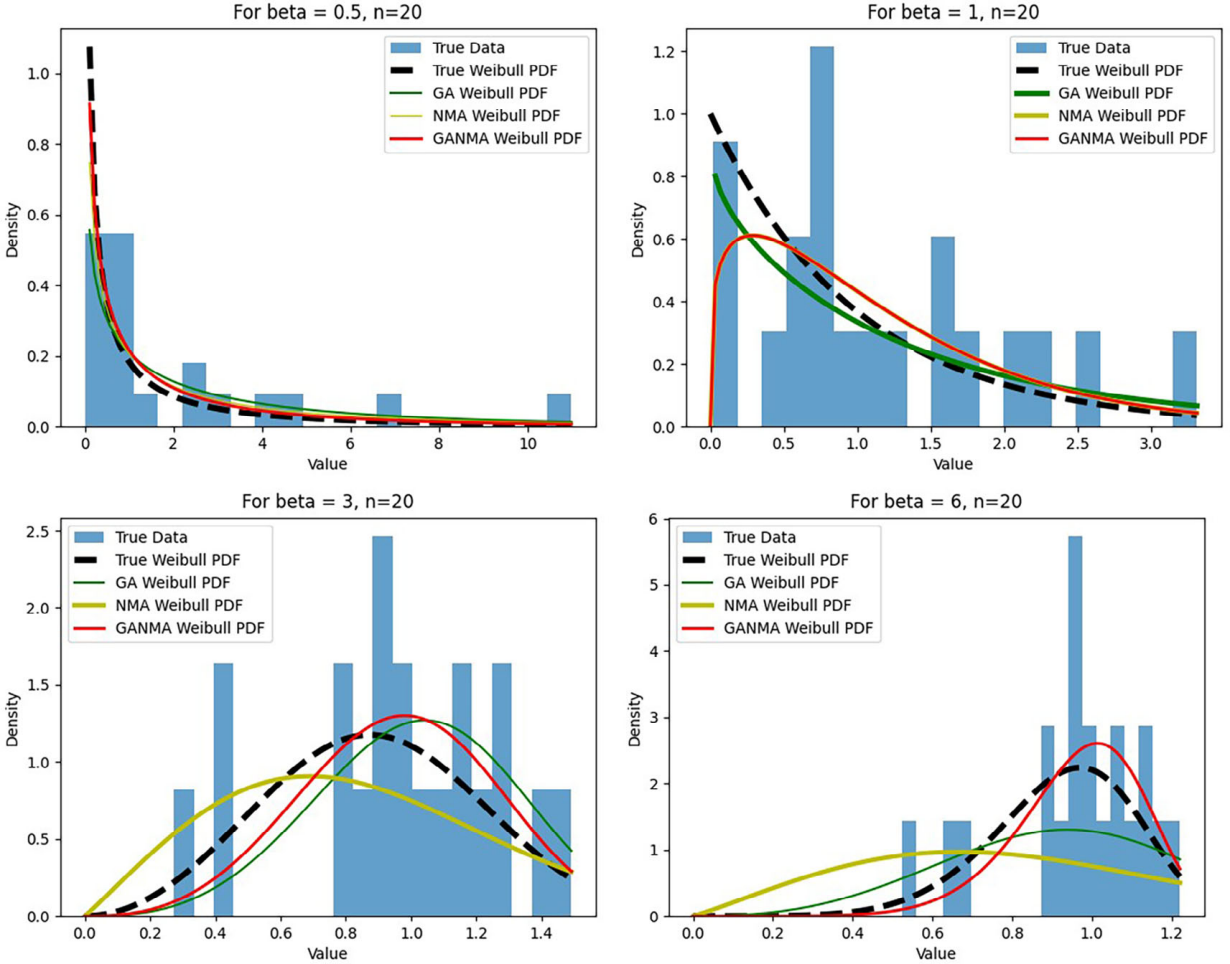
Histogram and MLE PDF of Weibull 2- parameter Distribution for *β* = 0.5, 1, 3, and 6 with n = 20.

**Figure 6.**
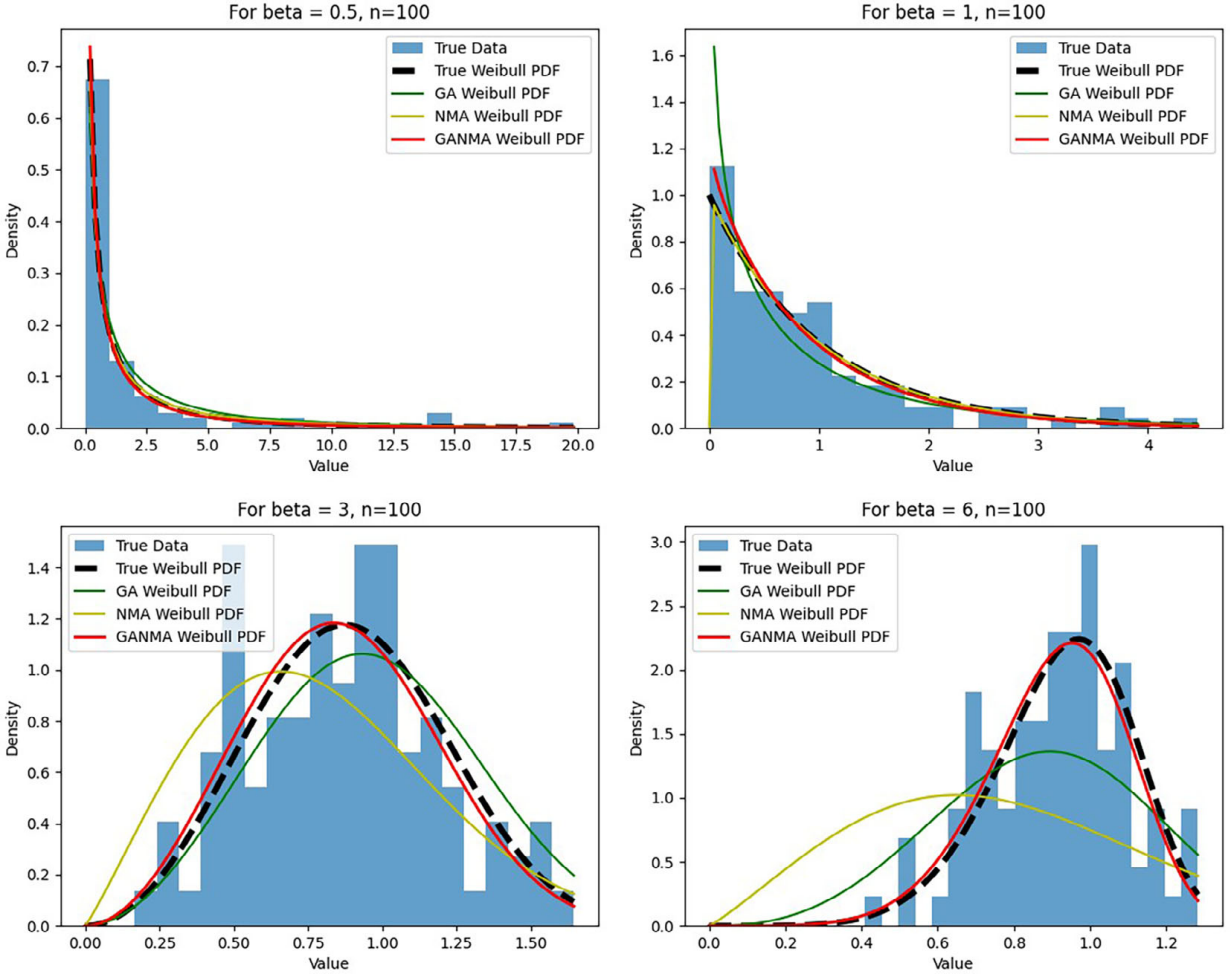
Histogram and MLE PDF of Weibull 2- parameter Distribution for *β* = 0.5, 1, 3, and 6 with n = 100.

**Figure 7.**
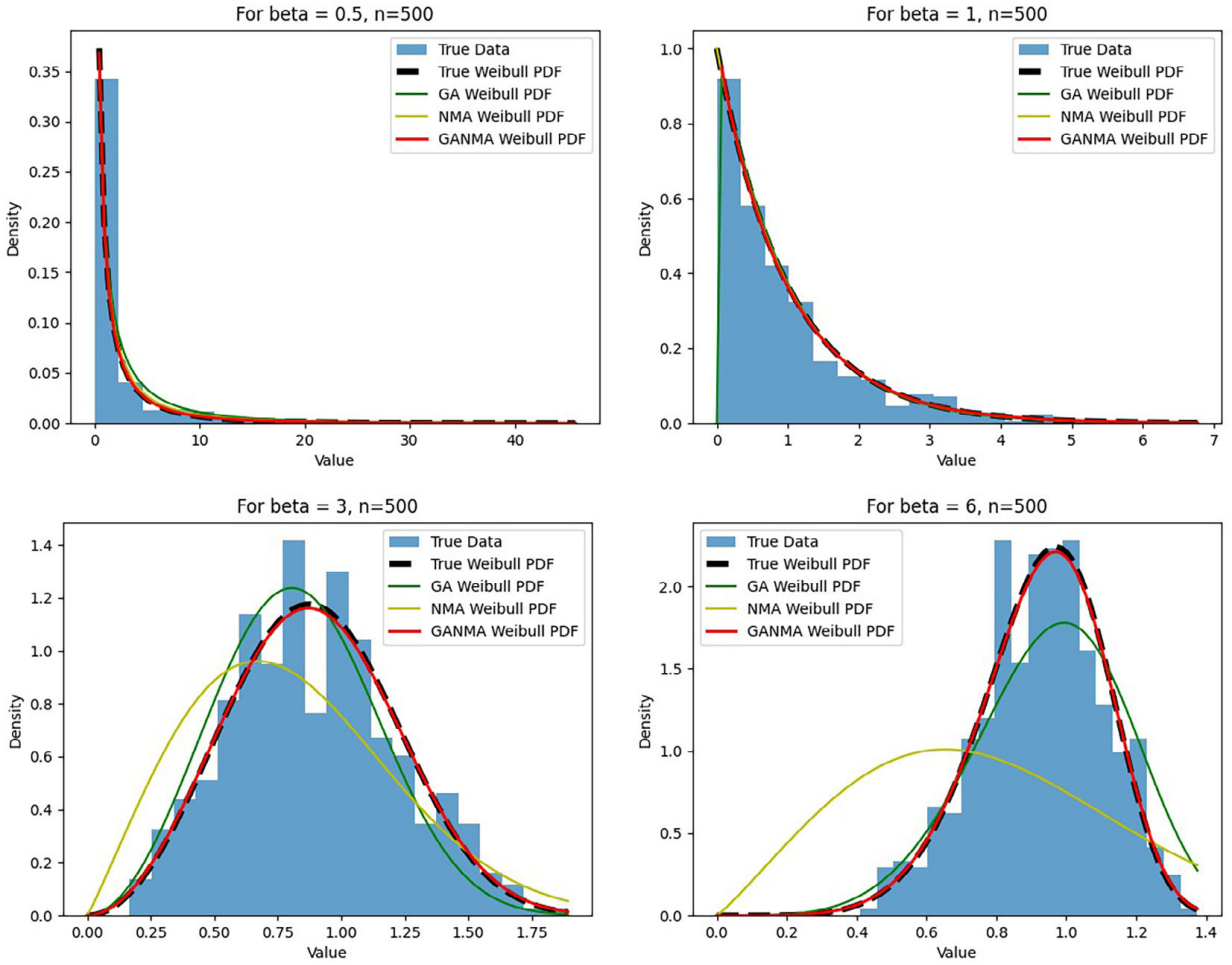
Histogram and MLE PDF of Weibull 2- parameter Distribution for *β* = 0.5, 1, 3, and 6 with n = 500.

Figures 5- 7 illustrate the outcome across various shape parameters while keeping the scale parameter constant—as well as various data sizes by plotting the convergence graph of the PDF of Weibull parameters and the PDF of MLE of parameters using NM, GA, and GANMA. The solid black line depicts the PDF of parameters ( *β*, *η*), whilst the usual genetic algorithm is illustrated by the solid green line, the yellow solid line shows the Weibull PDF using NM, and the suggested method GANMA is shown by the solid red line. It has been found that parameter estimation using the suggested technique converges with the original PDF as the shape parameter and data size increase. GANMA, the suggested algorithm, performs better than GA and NM in all types of situations.

Based on MAE and bias criteria, the simulation results demonstrate that the GANMA technique outperformed NM and GA in the estimation of shape and scale parameters. In each simulated scenario, the GANMA technique yielded the best shape parameter efficiency in terms of bias and MAE for sample sizes of 20, 100, and 500 respectively.

**Figure 8.**
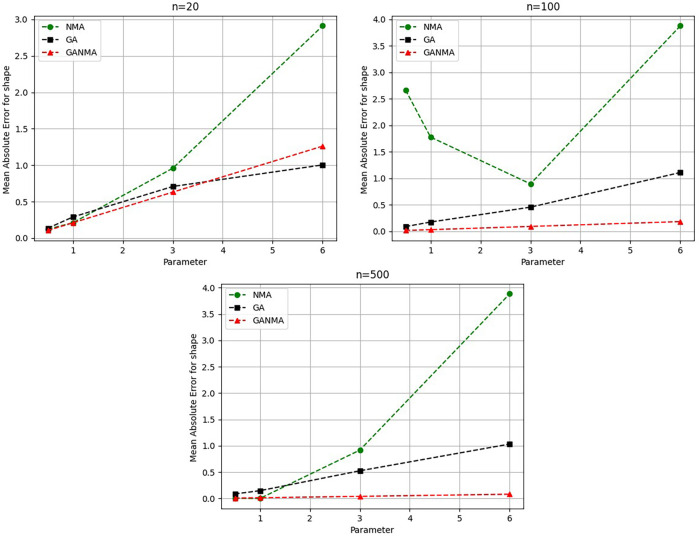
Comparison of parameter estimate approaches for *β* using the MAE criteria.

**Figure 9.**
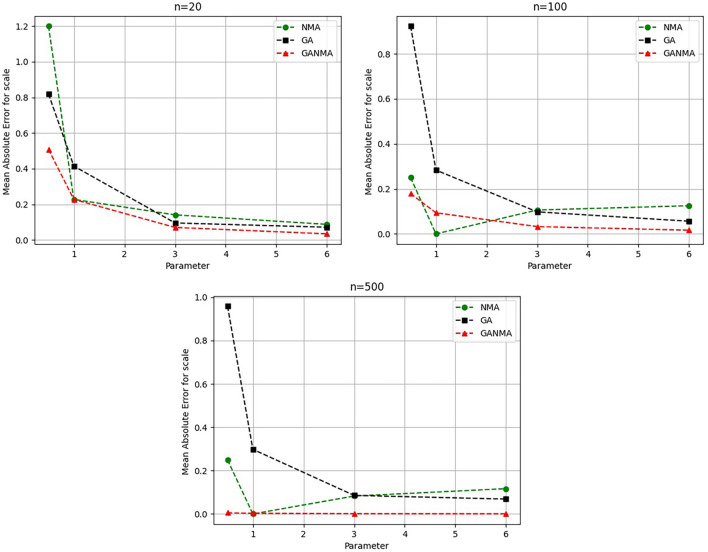
Comparison of parameter estimate approaches for *η* using the MAE criteria.

**Figure 10.**
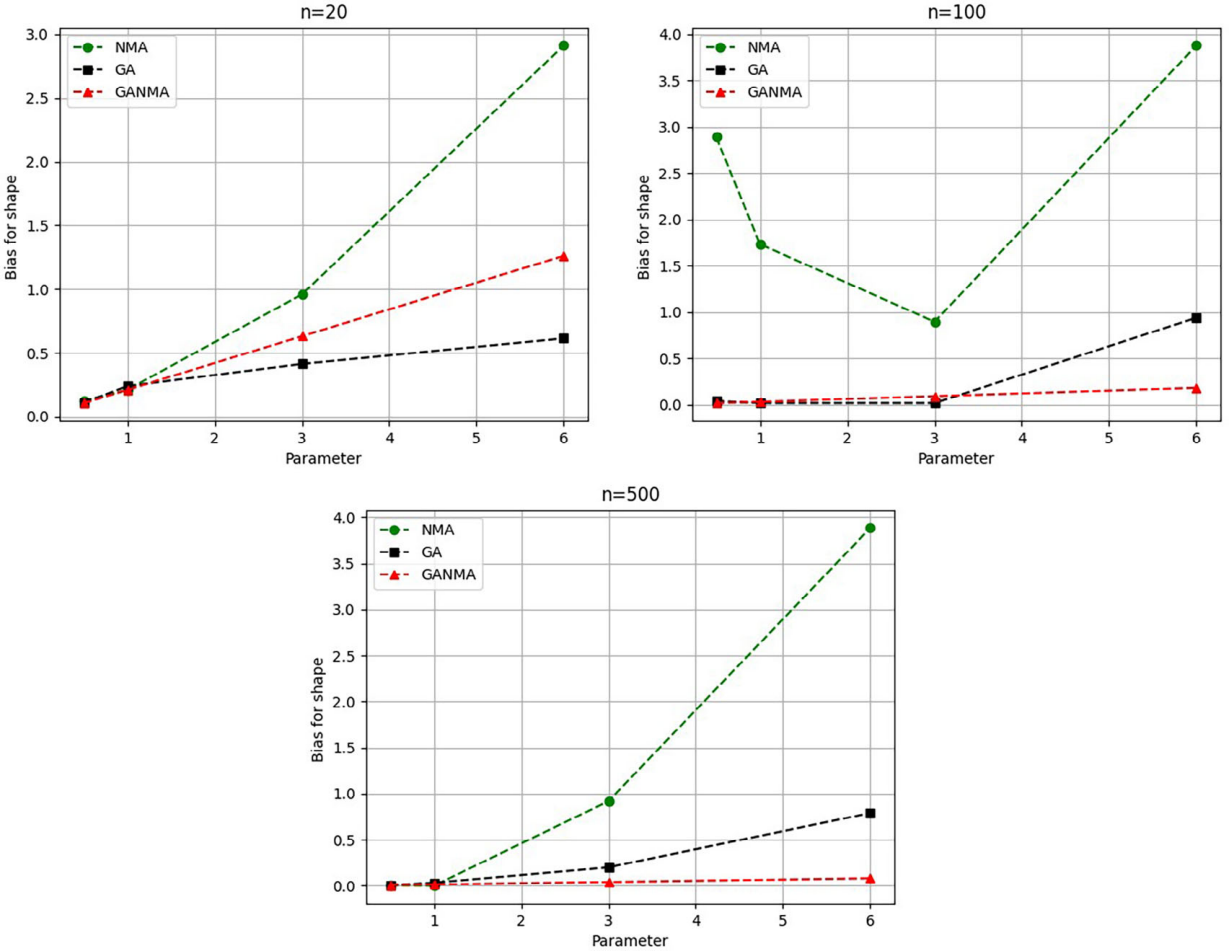
Comparison of parameter estimate approaches for β using the bias criteria.

**Figure 11.**
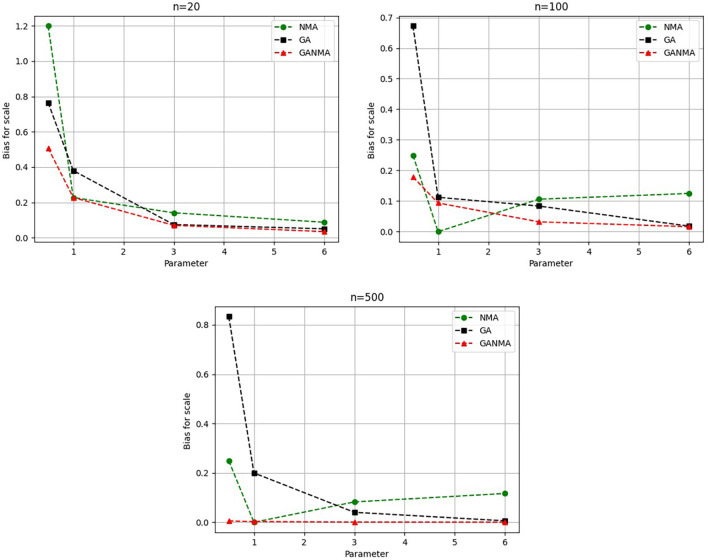
Comparison of parameter estimate approaches for η using the bias criteria.

Throughout almost every simulated scenario, GANMA achieved the maximum efficiency in the estimate of scale parameters for sample sizes of 20, 100, and 500, based on at least one decision criterion. By analyzing MAE and bias for each simulation scenario, GANMA proved to be the most effective approach for the data size 20. For small, moderate, and high sample sizes, GANMA is a fairly effective strategy overall. Additionally shown in Figures 8- 11 are the absolute values of the biases and the MAE.

The MAE values for the shape parameter *β* are shown in Figure 8. In every simulated scenario, GANMA outperformed NM and GA in terms of efficiency. The second-best approach is NM. An increase in sample size resulted in lower MAE values. On the other hand, MAE values increased along with an increase in the form parameter value.

The scale parameter *η*’s MAE values are displayed in Figure 9. For sample sizes of 20, 100, and 500, GANMA proved to be the most effective approach. When the shape parameter is set to a higher value, the MAE values drop. Likewise, as the sample size is raised, the MAE values drop.

The shape parameter *β*’s absolute bias value is displayed in Figure 10. The most efficient results were obtained using GANMA. NM outperformed GA on some occasions. As with MAE values, larger sample sizes resulted in lower absolute bias levels. Increasing the parameter value resulted in higher absolute bias levels.

The absolute bias for the scale parameter *η* is shown in Figure 11. Most of the time, GANMA outperformed other methods in terms of efficiency. The second-best approach is NM. Increasing the shape parameter and sample size leads to lower absolute bias levels.

## 8. Estimation of Weibull-Parameter in Wind speed analysis

The decrease in fossil fuel supplies and their lack of reliability in meeting future energy demands have made renewable energy a hot topic for academics. Wind is one of the main sources of renewable energy, and wind speed modeling has been studied in great detail. In wind power applications, the most popular Weibull distribution is two parameters. It has been discovered that this PDF is correct for the majority of wind regimes observed in nature, is easy to use, and is adaptable. In several research, it has been noted that the wind speed data cannot be adequately represented for specific applications, including those with bimodal distributions, short time horizons, low and high wind speeds, and domains with a high frequency of nulls. ^12^^,^^29^^,^^30^ The given equation may be used to determine the probability density function.

$$f\left(v\right)=\frac{\beta }{\eta }{\left(\frac{v}{\eta }\right)}^{\beta -1}e^{-{\left(v/\eta \right)}^{\beta }}$$ (14)

where *v* is wind speed.

**Power density**

Power density in wind speed analysis refers to the amount of power that can be obtained from the wind per unit area. This statistic is critical when evaluating the feasibility and potential viability of wind energy projects since it quantifies the energy available from the wind at a given place. The power density ( *P _D_*) may be easily calculated using the following equation once *β* and *η* have been established.

$$P_{D}=\frac{\rho_{a}\;\eta^{3}}{2}\;\frac{3}{\beta }\;\Gamma \left(\frac{3}{\beta }\right)$$ (15)

where, $\rho_{a}$ is the air density and symbol Γ denotes the gamma function. The standard value of air density $\rho_{a}$ is taken as $\rho_{a}=1.225\;\text{kg}/m^{3}$ .

### 8.1. Result and Discussion

In this challenge, two real-world data sets have been used to examine wind-speed analysis. The very first set of data came from the seas surrounding the Maluku Islands and Sulawesi. The data under analysis were gathered by the satellite Quikscat, which measured the ocean wind 10 meters above sea level using a scatterometer. The measurement’s horizontal and vertical spatial resolution is 0.25°earth grid. The information from the January measurement point at latitude 116° and longitude 85.5° is included in the accessible data. ^31^

Tarama Island and Iriomote Island, which are close to northern Taiwan, had their wind speeds recorded in the second data set. At Iriomotejima Meteorological Station, the maximum daily wind speed and direction were recorded in March 2012. ^32^

The Kolmogorov-Smirnov (K-S) test is a nonparametric statistical test used to compare two distributions. The K-S test calculates the maximum absolute difference between the empirical cumulative distribution functions (ECDFs) of the distributions being compared, providing a test statistic (D). A p-value derived from this statistic indicates the significance of the difference, helping in goodness-of-fit testing, comparing sample distributions, and model validation without assuming any specific distribution for the data.

The statistical confirmation that the monthly data sets come from the Weibull distribution can be obtained by doing the K-S test separately for each data set. The most significant difference between the theoretical distribution, *S _N_* ( *x*), and the observed distribution, *F*_0_( *x*), is the K-S test statistic. ^33^

$$D=\max|F_{o}\left(x\right)-S_{N}\left(x\right)|$$ (16)

Monthly distributions from the Weibull distribution are selected for further investigation following the K-S test (p−value≥0.05 ), which indicates the probability of observing a discrepancy as large as the one computed if the two distributions were the same.

**Figure 12.**
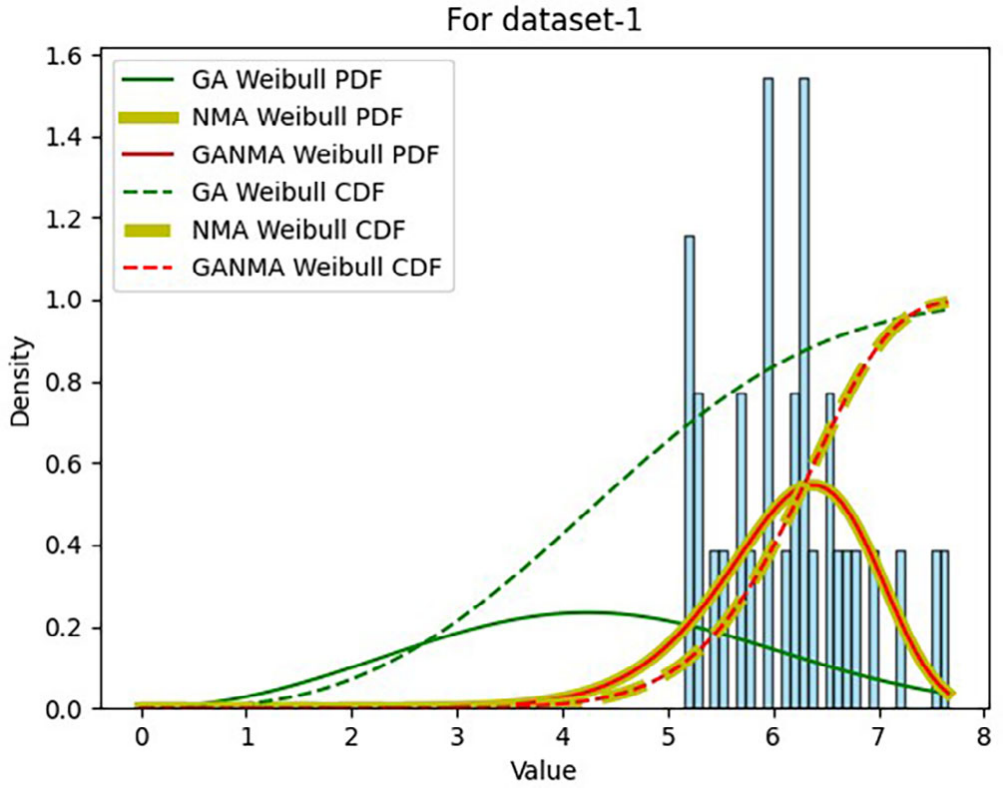
Histogram, MLE PDF and CDF using GA, NM, and GANMA for data set 1.

**Figure 13.**
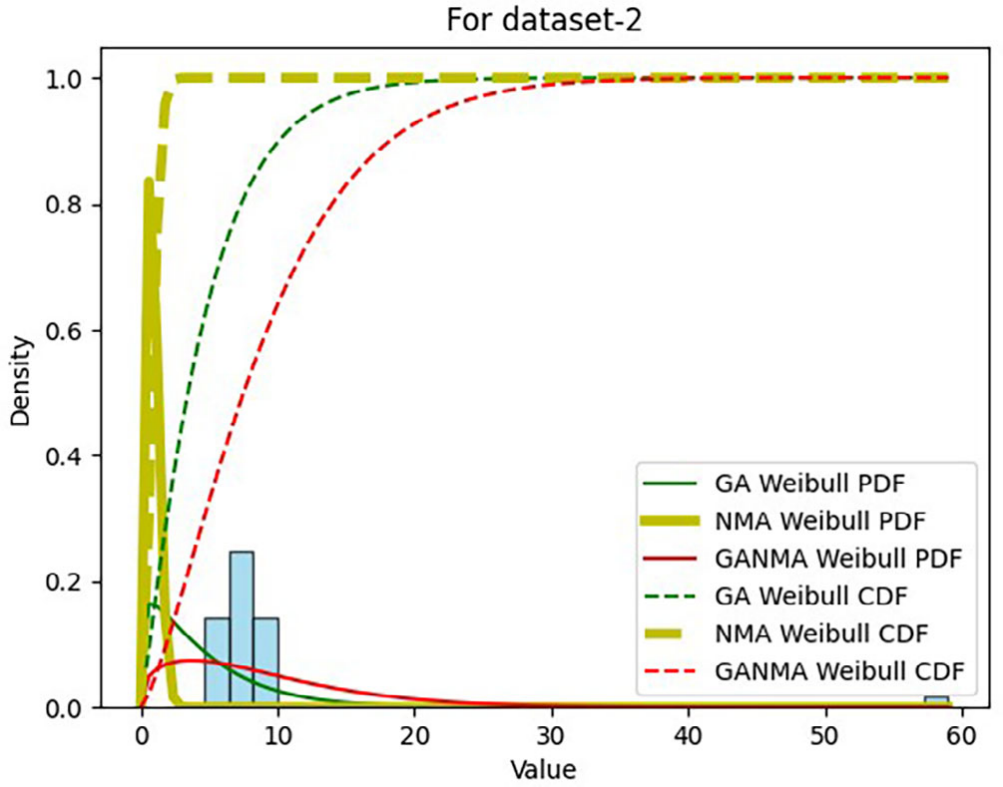
Histogram, MLE PDF and CDF using GA, NM, and GANMA for data set 2.

Results across shape and scale parameters were obtained by plotting the convergence graph between the PDF and CDF of MLE of parameters using NM, GA, and GANMA, as shown in Figures 12 and 13. The solid green line and dotted green line represent the PDF and CDF of the standard genetic algorithm, the yellow solid line, and dotted yellow line represent the Weibull PDF and CDF using NM, and the solid red line and dotted red line represent the suggested method for both the PDF and CDF, respectively. Figure 12 illustrates that the PDF and CDF for both GANMA and NM convergence are on the same line.

Tables 6 and 7 present the shape and scale parameters, k-s value, p-value, and power density for the first and second data sets, respectively, for all three estimation techniques. The greatest p-value and the lowest k-s statistic for both data sets are produced by the suggested approach (GANMA) out of the three estimation techniques. The Weibull distribution and the actual wind speed data seem similar, as indicated by the p-value exceeding the selected significance threshold (e.g., 0.05). In other words, the data is well-fitted by the Weibull distribution. The parameters estimated using GANMA are considered the best fit for describing the wind speed data, based on the K-S test findings. The observed wind speed data and the predicted Weibull distribution with these parameters were well recognized, as evidenced by the low K-S statistic and high p-value.

**Table 6.** Parameter estimations for data set-1.

Method	$\hat{\beta }$	$\hat{\eta }$	k-s value	p-value	P _D_ (watt/m ^2^)
NM	9.52382	6.44868	0.13685	0.56069	147.05921
GA	3.48312	4.97007	0.67756	1.274E-14	71.36770
GANMA	9.52340	6.44863	0.13682	0.560978	147.05541

**Table 7.** Parameter estimations for data set-2.

Method	$\hat{\beta }$	$\hat{\eta }$	k-s value	p-value	P _D_ (watt/m ^2^)
NM	2.0	1.0	0.99999	2.63E-285	0.81422
GA	1.07418	4.97152	0.62512	3.105E-12	350.27224
GANMA	1.35925	9.85611	0.35982	0.00042	1431.63678

The maximum power density is demonstrated by the parameters estimated through MLE implementing NM, as shown in Table 6. This suggests that the parameters possess greater absolute performance in terms of power generation. Despite the slightly lower power density value of the parameters estimated by MLE using GANMA compared to NM, they are nevertheless selected as the best fit since they have the greatest p-value and the least k-s statistic. This suggests that for wind speed data set 1, parameters calculated by MLE using GANMA offer the best match.

The parameters that are estimated by MLE using GANMA are found to provide the best fit in Table 7, as shown by their lowest K-S statistic and highest p-value. Additionally, superior performance in terms of power generation is indicated by the higher power density value associated with these parameters.

## 9. Conclusion

To improve the exploitation capabilities of GA, this study presents a unique hybridized approach called the Genetic and Nelder-Mead Algorithm (GANMA), in which NM is included. GANMA has been employed to verify the robustness and efficiency of the suggested technique on fifteen benchmark problems for three separate dimensions. Because of its high level of accuracy and stability, GANMA performs very well in improving unimodal, multimodal, and shifting unimodal/multi-modal functions, as shown by the test function comparison experiment table. According to the testing results, the suggested method is strong and has the potential to solve benchmark issues more quickly than the other two algorithms in the majority of situations.

Furthermore, estimating the Weibull distribution’s scale ( *η*) and shape ( *β*) parameters, this study aims to assess the efficacy of three estimation methods: ML estimators employing GA, NM, and GANMA. The MAE and bias criteria are used to assess the efficiency of the parameter estimating techniques. Based on the conclusions drawn from the Monte Carlo simulation and the examination of real-world wind speed data, the ML estimator using GANMA performs better in Weibull parameter estimation than the ML estimator using NM and GA estimator. We used the K-S test to compare three sets of parameters for two fitting wind speed data sets with a Weibull distribution and selected the set of parameters that minimized the K-S statistic and maximized the associated p-value, indicating the best fit. Moreover, it may be said that the two sets of data were collected in two different geographic locations with different meteorological conditions. In these data sets, which included a variety of meteorological situations, GANMA demonstrated superiority.

## Compliance with ethical standards

### Disclosures & disclaimer

We certify that the submitted manuscript is our original work that is not currently being considered elsewhere. The paper is an unfunded independent piece of labor.

### Ethical approval

This article does not include any research that any of the authors conducted using humans or animals.

## Data Availability

All data supporting the findings of this study, including figures and tables, have been deposited in the given link; https://doi.org/10.5281/zenodo.13309711 ^34^ The files are as follows: [Data Sets] data has been obtained from a third party for two real-life problems, which are available at data sets.docx The extended data files are available in Zenodo at the following DOI: [ https://doi.org/10.5281/zenodo.13309711.v3] ^34^ [Algorithm] The algorithm described in the manuscript available at algorithm.docx The files included: raw data of functions.docx and raw data of functions(generation wise).docx Contains data analysis that supports the study but are not included in the main manuscript. [Tables] This file contains all the tables referenced in the manuscript. tables.docx [Figures] This file contains all the figures referenced in the manuscript, including detailed captions. figures.docx Data are available under the terms of the Creative Commons Attribution 4.0 International license (CC-BY 4.0).

## Software availability

Python is an open-source source and is available at https://python.org

Archived software available from: https://doi.org/10.5281/zenodo.13309711 ^34^ (source code.zip) where archived source code can be accessed.
