# A novel hybrid genetic algorithm and Nelder-Mead approach and it’s application for parameter estimation

**DOI:** 10.12688/f1000research.154598.2

**Published:** 2025-03-10

**Authors:** Neha Majhi, Rajashree Mishra

**Affiliations:** 1 Mathematics, Kalinga Institute of Industrial Technology, Bhubaneswar, Odisha, 751024, India

**Keywords:** Genetic Algorithm, Maximum Likelihood Estimation, Nelder-Mead algorithm, Power Density, Weibull Distribution, Wind speed analysis

## Abstract

**Background:**

Traditional optimization methods often struggle to balance global exploration and local refinement, particularly in complex real-world problems. To address this challenge, we introduce a novel hybrid optimization strategy that integrates the Nelder-Mead (NM) technique and the Genetic Algorithm (GA), named the Genetic and Nelder-Mead Algorithm (GANMA). This hybrid approach aims to enhance performance across various benchmark functions and parameter estimation tasks.

**Methods:**

GANMA combines the global search capabilities of GA with the local refinement strength of NM. It is first tested on 15 benchmark functions commonly used to evaluate optimization strategies. The effectiveness of GANMA is also demonstrated through its application to parameter estimation problems, showcasing its practical utility in real-world scenarios.

**Results:**

GANMA outperforms traditional optimization methods in terms of robustness, convergence speed, and solution quality. The hybrid algorithm excels across different function landscapes, including those with high dimensionality and multimodality, which are often encountered in real-world optimization issues. Additionally, GANMA improves model accuracy and interpretability in parameter estimation tasks, enhancing both model fitting and prediction.

**Conclusions:**

GANMA proves to be a flexible and powerful optimization method suitable for both benchmark optimization and real-world parameter estimation challenges. Its capability to efficiently explore parameter spaces and refine solutions makes it a promising tool for scientific, engineering, and economic applications. GANMA offers a valuable solution for improving model performance and effectively handling complex optimization problems.

## 1. Introduction

In the continuous pursuit of optimization, where achieving the finest possible outcomes with utmost efficiency and accuracy is crucial, the fusion of diverse methodologies frequently yields superior solutions. Optimization algorithms are always looking for ways to improve efficiency and robustness, encouraging professionals and scholars to investigate novel ideas that happen to be mostly inspired by nature or mathematical concepts. Hybridization in optimization algorithms has garnered significant attention in recent years, offering a potent means to enhance efficacy and efficiency. Among these methodologies, Genetic Algorithms (GA) and the Nelder-Mead Simplex Algorithm (NM) emerge as prominent contenders, each boasting distinct advantages and applications. However, the fusion of these theories has recently proven to be an enticing strategy for enhancing optimization capabilities across various domains.

Inspired by evolution and natural selection, genetic algorithms operate by repeatedly developing a population of potential solutions over a series of generations. The concepts of genetic recombination and survival of the fittest are collectively mirrored by the selection, crossover, and mutation operators involved in this evolutionary process. GAs are a popular choice in various industries, including engineering, finance, and biology, because of their impressive effectiveness in solving complicated, high-dimensional optimization problems with non-linear and multimodal objective functions.

The Nelder-Mead Simplex Algorithm, on the other hand, provides a geometric method for repeatedly refining a simplex—a multi-dimensional geometric shape in the direction of the ideal solution. Its foundation is mathematical optimization. Nelder- Mead algorithms are especially well-suited for problems with few variables or smooth objective functions because, in contrast to GAs, which rely on a population-based approach, they operate on a single point or simplex at each iteration. Due to its ease of use, simplicity, and speedy convergence to local optima, it now has a deserving place within the optimization toolbox.

Genetic Algorithm (GA) based hybrids have emerged as powerful tools for optimization, combining GA’s global search capabilities with the local refinement strengths of other algorithms. These hybrids balance exploration and exploitation, allowing for efficient navigation of complex, high-dimensional search spaces. However, they are not without limitations. Challenges such as slow convergence rates, parameter sensitivity, and computational overhead remain prevalent. Furthermore, many existing studies lack comprehensive comparisons of hybrid methodologies and fail to explore their scalability and adaptability to diverse optimization problems. Gaps also persist in understanding the interplay between exploration and exploitation in these hybrids, leaving room for novel approaches that address these issues.

### Overview of Recent GA Hybrids

Genetic Algorithm (GA) based hybrid approaches have become a cornerstone in modern optimization research, combining GA’s global exploration abilities with various techniques to enhance local refinement. The following highlights some key advancements:

1. **GA-Nelder–Mead (GA-NM):** ^1^
  • Methodology: Integrates GA for its broad search capabilities with the Nelder–Mead (NM) simplex algorithm, which excels in refining solutions locally.
  • Strengths: Offers improved convergence speeds and precision in parameter estimation, effectively balancing exploration and exploitation.
  • Weaknesses: Struggles with scalability in higher dimensions and demands careful tuning of parameters for optimal performance.
2. **GA-Harris Hawks Optimization (GA-HHO):** ^2^
  • Methodology: Combines GA’s exploration strength with the Harris Hawks Optimization (HHO) method for exploiting promising regions.
  • Strengths: Demonstrates exceptional performance in handling complex, multimodal optimization problems.
  • Weaknesses: Computational demands increase significantly, and parameter sensitivity can affect robustness.
3. **Real-Value Genetic Algorithm and Extended Nelder–Mead (RVGA- ENM):** ^3^
  • Methodology: Employs RVGA for global searches and the Extended Nelder–Mead (ENM) algorithm for refining solutions, specifically applied to energy demand forecasting.
  • Strengths: Achieves superior accuracy in predictions and effective refinement of solutions.
  • Weaknesses: Highly reliant on the quality of the initial population and available training data.
4. **GA-Tabu Search (GA-TS):** ^4^
  • Methodology: Utilizes GA for broad search capabilities and Tabu Search (TS) for local optimization, designed for maintenance scheduling in cogeneration plants.
  • Strengths: Efficiently handles scheduling challenges in complex systems.
  • Weaknesses: Suffers from significant computational overhead as the problem size grows.
5. **GA-Machine Learning (GA-ML):** ^5^
  • Methodology: Integrates GA with machine learning (ML) models to optimize graph-related problems.
  • Strengths: Provides adaptability and enhanced performance through insights derived from ML techniques.
  • Weaknesses: Complexity increases due to the integration of ML, leading to greater computational requirements.
6. **Harris Hawks-Nelder–Mead (HH-NM):** ^6^
  • Methodology: Combines HHO and NM for tackling optimization in design and manufacturing scenarios.
  • Strengths: Demonstrates strong convergence performance and resilience in solving intricate problems.
  • Weaknesses: Requires fine-tuned parameter adjustments to maintain consistency.
7. **GA-Artificial Neural Network (GA-ANN):** ^7^
  • Methodology: Couples GA with Artificial Neural Networks (ANNs) for optimizing process parameters, particularly in plastic injection molding.
  • Strengths: Effectively enhances manufacturing quality and process efficiency.
  • Weaknesses: Dependence on ANN training data can limit its applicability to diverse scenarios.
8. **GA-Simulated Annealing (GA-SA):** ^8^
  • Methodology: Merges GA’s exploratory capabilities with Simulated Annealing’s (SA) temperature-based refinement strategy.
  • Strengths: Efficiently escapes local optima and maintains diversity in the search process.
  • Weaknesses: Computational costs are high, with slower convergence for high-dimensional tasks.
9. **GA-Particle Swarm Optimization (GA-PSO):** ^9^
  • Methodology: Combines GA’s global exploration with Particle Swarm Optimization (PSO) for exploiting solutions.
  • Strengths: Performs exceptionally well in multimodal optimization landscapes.
  • Weaknesses: Risks stagnation in local optima if not equipped with adaptive mechanisms.
10. **GA-Nelder-Mead (GA-NM):** ^10^
  • Methodology: Utilizes the NM simplex method within GA to enhance solution precision in smooth, low-dimensional problems.
  • Strengths: Improves optimization precision through effective local refinement.
  • Weaknesses: Faces challenges in scalability and requires precise parameter settings.

These developments illustrate the versatility and potential of GA hybrids in addressing a range of optimization challenges while emphasizing the need for careful parameter tuning and scalability enhancements. GANMA builds on this foundation by offering a structured, robust framework that addresses existing limitations.

Despite advancements in hybrid optimization algorithms, several key challenges persist. Many studies lack comprehensive comparisons, failing to evaluate scalability, convergence, and adaptability across diverse tasks. Additionally, the balance between global exploration and local exploitation remains under-explored, limiting efficiency in finding optimal solutions. Scalability issues are prominent, as many hybrids falter in high-dimensional problems, highlighting the need for robust methods capable of maintaining performance in complex spaces. Parameter sensitivity is another hurdle, with insufficient adaptive tuning mechanisms leading to inconsistent results. Furthermore, validation is often restricted to benchmark functions, offering limited insight into real world applicability where constraints and objectives are more complex. These gaps emphasize the need for innovative hybrids that address these issues while ensuring efficiency, scalability, and practical relevance.

Individually, both GA and NM have strengths and limits that make them appropriate for specific optimization scenarios. GA excels in global exploration, utilizing population variety to explore large solution spaces and avoid local optima. On the other hand, NM excels at local refinement, expertly traversing convex and smooth terrain to locate specific optima. The hybridization of GA with NMA addresses the limitation of GA in fine-tuning solutions near optima, at which NMA excels. This synergy improves the algorithm’s convergence speed and solution quality. Other researchers have primarily focused on individual optimization methods or hybridizations excluding GA and NMA, leaving a gap in fully exploiting the complementary strengths of these methods.

The GANMA method effectively addresses these gaps through its innovative design and balanced approach. By seamlessly integrating Genetic Algorithm (GA) and Nelder-Mead Algorithm (NM), GANMA achieves a robust balance between global exploration and local exploitation, enhancing its efficiency in diverse optimization tasks. Its structured framework allows for improved scalability, maintaining performance even in high-dimensional problem spaces. Additionally, GANMA incorporates adaptive mechanisms for parameter tuning, reducing sensitivity and ensuring consistent results across various scenarios. Unlike many existing hybrids, GANMA has been rigorously tested on both benchmark functions and real-world parameter estimation tasks, demonstrating its adaptability and robustness. These features position GANMA as a superior hybrid optimization method, addressing the limitations of existing approaches while offering practical solutions for complex, multidimensional challenges.

Many industries are interested in using the Nelder-Mead Simplex Algorithm (NM) working together with Genetic Algorithms (GA), including bio-informatics, ^11^ finance, ^12, 13^ and engineering. ^14, 15^ Combining these methods provides a potent method of resolving challenging optimization issues in engineering, where designs are complicated and rules are demanding. Combining GA with NM helps improve portfolio management and risk assessment in the financial industry, where on-time and correct choices are essential. Similarly, hybrid algorithms speed up tasks like genomic analysis and drug discovery ^16, 17^ in bio-informatics, where understanding biology relies on smart computer methods. This article explores how combining NM and GA enhances both, highlighting how they work together to solve real-world issues. This paper has tested the GANMA algorithm on fifteen benchmark problems in three dimensions (10, 20, and 30). According to the results from the experiment, the suggested GANMA algorithm is a promising one that can quickly find the best or almost the best solution for most of the functions examined.

The remaining portion of the research study is structured as follows: Section 2 provides the fundamentals of the Genetic Algorithm and the Nelder-Mead simplex search. Section 3 discusses the proposed hybridized method, benchmark functions, and an alternative hybridization approach. The parameter setup for all methods and computational configurations are detailed in Section 4. Section 5 presents the results and discussion for benchmark functions, while Section 6 focuses on the Weibull distribution. Parameter estimation methods are described in Section 7, and Section 8 provides an analysis of Monte Carlo simulations and results. Two real-world wind speed datasets are analyzed in Section 9 to demonstrate the effectiveness of the proposed technique. Finally, Section 10 concludes the study with key observations.

## 2. Overview of GA, and NM

A brief overview of GA, and NM have been described below.

### 2.1 Real-Coded Genetic Algorithm (GA)

Metaheuristic optimization methods like GA are higher-level frameworks designed to guide heuristic or local search procedures. In contrast, heuristic searches are problem-specific strategies for exploring the solution space. GA leverages metaheuristic principles to perform heuristic searches iteratively, balancing exploration and exploitation. So, we can say that GA is an approach to heuristic search. The ideas of the biological evolution of species serve as its inspiration. In contrast to traditional optimization methods, GA ^11^^,^^18^ starts with a collection of starting solutions known as chromosomes.

Genetic algorithms (GAs) work by continually improving solutions based on their fitness, which measures how well they solve a problem. Unlike some traditional methods, GAs don’t assume anything about the problem, like whether it’s smooth or has just one best solution. Instead, they explore different possibilities to find good solutions, even in complex situations where there might be many equally good answers. GAs have been used successfully in many difficult optimization problems. They often work better than traditional methods, especially when there are multiple equally good solutions. This flexibility and ability to handle complex situations make GA a valuable tool for solving optimization problems in various fields.

Following is a summary of the GA stages in this study:

I. Initialization:
  • First, create a vector of real values for each variable between predefined ranges. This vector will represent the initial population of individuals.
II. Evaluation:
  • Evaluate the fitness of each individual using an objective function.
III. Selection:
  • Select individuals from the population to create a mating pool based on their fitness values.
IV. Crossover (Recombination):
  • Pair selected individuals and perform crossover to create offspring by blending or combining their real values.
V. Mutation:
  • Introduce random changes to the real values of offspring to promote exploration of the search space.
VI. Combining Populations:
  • Combine the offspring generated from crossover and mutation with the initial population.
VII. Sorting:
  • The combined population is sorted based on their fitness levels, with the most fit people having the lowest fitness values.
VIII. Elitism:
  • Keep only the top half of the sorted population, discarding the bottom half. This ensures that the best-performing individuals from the previous generation are preserved for the next generation.
IX. Termination:
  • Steps 2 through Step 8 should be repeated for the designated number of generations or until a termination criterion—such as achieving a maximum number of iterations or reaching a certain fitness level is satisfied.

This approach with elitism helps maintain diversity in the population while ensuring that the best individuals are preserved across generations, ultimately leading to the discovery of better solutions in the optimization process. Real-coded genetic algorithms are suitable for optimization problems with continuous decision variables and offer advantages such as direct representation of real-valued solutions, robustness, and ability to handle high-dimensional search spaces.

### 2.2 Nelder–Mead Simplex Search Method (NM)

The simplex search technique has been widely used for basic unconstrained minimization problems, such as nonlinear least squares, nonlinear simultaneous equations, and general function minimization. ^19^ Originally proposed by Spendley, Hext, and Himsworth (1962), ^20^ the method was later refined by Nelder and Mead (1965) ^21^ to improve its efficiency and applicability.

The Nelder-Mead Algorithm (NMA) is selected for its simplicity and effectiveness in local solution refinement, making it a strong complement to the Genetic Algorithm’s (GA) global search capabilities. While a variety of optimization algorithms exist, NMA’s low computational overhead and reliability in small-dimensional spaces make it an efficient and practical choice for hybridization.

However, NMA’s reliance on simplex geometry and localized operations restricts its exploratory capacity, often causing it to converge prematurely to local optima in complex, multi-modal landscapes. Preliminary experiments (to be included) under these limitations highlight the necessity of GA’s global search to overcome such challenges.

The steps of the Nelder-Mead ^21^^,^^22^ algorithm are summarized in as follows:

I. Initialization:
  • A simplex is a collection of *n* + 1 vertices in a *n* dimensional space. These vertices can be deliberately selected or created at random.
  • At every simplex vertex, evaluate the objective of the function.
II. Ordering:
  • Order the vertices based on their corresponding function values.
  • Let *x*_1_ *, x*_2_ *, …, x*_*n*+1_ denote the vertices such that *f* ( *x*_1_) ≤ *f* ( *x*_2_) ≤ *…* ≤ *f* ( *x*_*n*+1_).
III. Centroid:
  • Calculate the centroid of each vertex, except the worst (highest) one:
$$x_{\text{centroid}}=\frac{1}{n}\sum_{i=1}^{n}x_{i}$$ (1)
IV. Reflection:
  • Reflect the worst vertex (highest) through the centroid to obtain a trial point
$$x_{r}=x_{\text{centroid}}+\alpha \left(x_{\text{centroid}}-x_{n+1}\right)$$ (2)
where *α* is a reflection coefficient, typically set to 1
  • Evaluate the objective function at $x_{r}$ .
V. Expansion:
  • Expanding further should be considered if the reflected point $x_{e}$ is superior to the second-worst vertex:
$$x_{e}=x_{\text{centroid}}+\gamma \left(x_{r}-x_{\text{centroid}}\right)$$ (3)
where γ is an expansion coefficient, usually γ>1

  • Evaluate the objective function at $x_{e}$ .
VI. Contraction:
  • Contraction should be done if the reflected point $x_{r}$ is worse than the worst vertex:
    – Outside contraction:
$$x_{c}=x_{\text{centroid}}+\rho \left(x_{r}-x_{\text{centroid}}\right)$$ (4)
    – Inside contraction:
$$x_{c}=x_{\text{centroid}}+\sigma \left(x_{\text{centroid}}-x_{r}\right)$$ (5)
where *ρ* is a contraction coefficient, typically 0 *< ρ, σ <*1 (typically 0.5).
  • Evaluate the objective function at $x_{c}$ .
VII. Update simplex:
  • Replace the worst vertex with the new trial point if it improves the function value.
VIII. Termination:
  • Till a termination criterion such as a maximum number of iterations, a small modification in step size, or a slight modification in the function value is satisfied, repeat the steps described above.

The algorithm converges when the simplex becomes sufficiently small or when the function values at the vertices are close to each other. The choice of parameters *α*, *γ*, and *ρ* can significantly affect the performance of the algorithm and may need to be tuned based on the problem characteristics.

## 3. Methods

### 3.1 Motivation

The combination of Genetic Algorithms (GA) with the Nelder-Mead simplex algorithm (NM) is driven by their supportive characteristics in both global exploration and local exploitation. GA is a population-based technique that effectively explores diverse sections of the search space, although fine-tuning solutions at local optima may provide issues. In contrast, it requires greater capacity for worldwide investigation. Combining both methods intends to take advantage of the characteristics of both algorithms, resulting in a more balanced and efficient optimization process. This hybridization method has the potential to improve convergence rates, solution quality, and robustness, making it a compelling choice for handling complicated optimization problems across several domains.

### 3.2 Genetic algorithm with Nelder-Mead Simplex Search (GANMA)

The suggested algorithm’s (GANMA) stages are summed up as follows:

I. Initialization:
  • Generate an initial population of solutions for the GA.
II. Evaluation:
  • Analyze each solution’s objective function within the population.
III. Genetic Algorithm (GA) Cycle:
  • Selection: Select a parent from the current population. Selection techniques that are often used include rank-based, roulette wheel, and tournament selection.
  • Crossover: Perform crossover to create offspring solutions. Since this is a real coded GA, a common method is the arithmetic crossover or simulated binary crossover.
  • Mutation: Apply mutation operators to the offspring solutions. Here is where the Nelder-Mead simplex algorithm comes into play. After mutation, the simplex is formed around the mutated solutions.
  • Elitism: Combine the initial population and offspring after mutation, then calculate the mean combination. Sort the combined population according to their fitness and keep the first half population while rejecting the other half.
  • Replacement: Replace the initial population with the best half from the previous step.
IV. Nelder-Mead Simplex Algorithm:
  • Define the simplex for the NM algorithm. This can be done by selecting a set of initial points around the best solution found by the GA so far. (The simplex in NMA is defined around the best GA solution to ensure the refinement starts near a promising region. This choice leverages GA's exploration strength, as demonstrated in our results section.)
  • Reflection: Take the centroid of the remaining points and reflect the worst point of the simplex.
  • Expansion: Attempt to extend the simplex in that direction if the reflected point is superior to the second-worst but not superior to the greatest.
    • Contraction: If neither reflection nor expansion produces a better point, contract the simplex towards the best point.
    • Update the simplex based on the chosen operation (reflection, expansion, contraction, or shrinkage).
    • Repeat the above steps until convergence criteria are met.
V. Termination:
  • Repeat the GA cycle and NM algorithm until a termination criterion is satisfied. This could be a maximum number of iterations, reaching a specific fitness threshold, or convergence of the simplex.
VI. Output:
  • The best solution found after the termination criterion is met.
  • Apply the Nelder-Mead simplex algorithm to the best solution.
VII. Optimal:
  • The best solution found after the NM algorithm.

NMA is applied to the best solution after reproduction and mutation in each iteration, not just the final solution. This strategy allows continuous refinement throughout the optimization process. By combining GA with NM in this way, you leverage the GA’s global exploration capability with the NM’s local refinement ability, potentially leading to improved convergence and robustness in optimization tasks.

GANMA stands out as a versatile hybrid algorithm capable of addressing a wide range of optimization problems, transcending the domain-specific focus of many existing hybrids. Its well-balanced framework effectively combines the global search power of Genetic Algorithms (GA) with the local refinement precision of the Nelder-Mead Algorithm (NMA), ensuring scalability, robustness, and efficiency. This synergy enables GANMA to overcome common challenges, such as parameter sensitivity and poor performance in high-dimensional or multimodal landscapes. Furthermore, GANMA’s structured approach is rigorously validated, making it a reliable solution for both theoretical benchmark functions and complex real-world applications.

The pseudo-code for the hybridization of the GA and Nelder-Mead simplex algorithm is presented in Algorithm 1.

Algorithm 1. **Combination of GA and Nelder-Mead.**

1: Initialize GA parameters (size of population, rate of mutation, rate of crossover, number of generations)

2: Initial population

3: **while** termination condition is not met **do**

4:   Evaluate each individual’s current level of fitness

5:   Select parents (using tournament selection) for crossover

6: **for** each pair of parents **do**

7:       Apply one-point crossover

8:       Apply uniform mutation

9: **end for**

10:   Combine initial population with offspring

11:   Evaluate the fitness of the combined population

12:   Sort the combined population by fitness

13:   Keep the top half of the sorted population

14:   Create a simplex from the best individuals (e.g., top 2)

15:   Perform Nelder-Mead steps on the simplex:

16:     - Reflection

17:     - Expansion

18:     - Contraction

19:     - Shrink

20:   Update the simplex

21:   Replace the worst individuals with the simplex’s best individuals

22:   Evaluate the fitness of the updated population

23: **end while**

24: From the final population, choose the best solution

25: Perform Nelder-Mead steps on the best solution

26: Find the optimal solution

**Figure 1.**
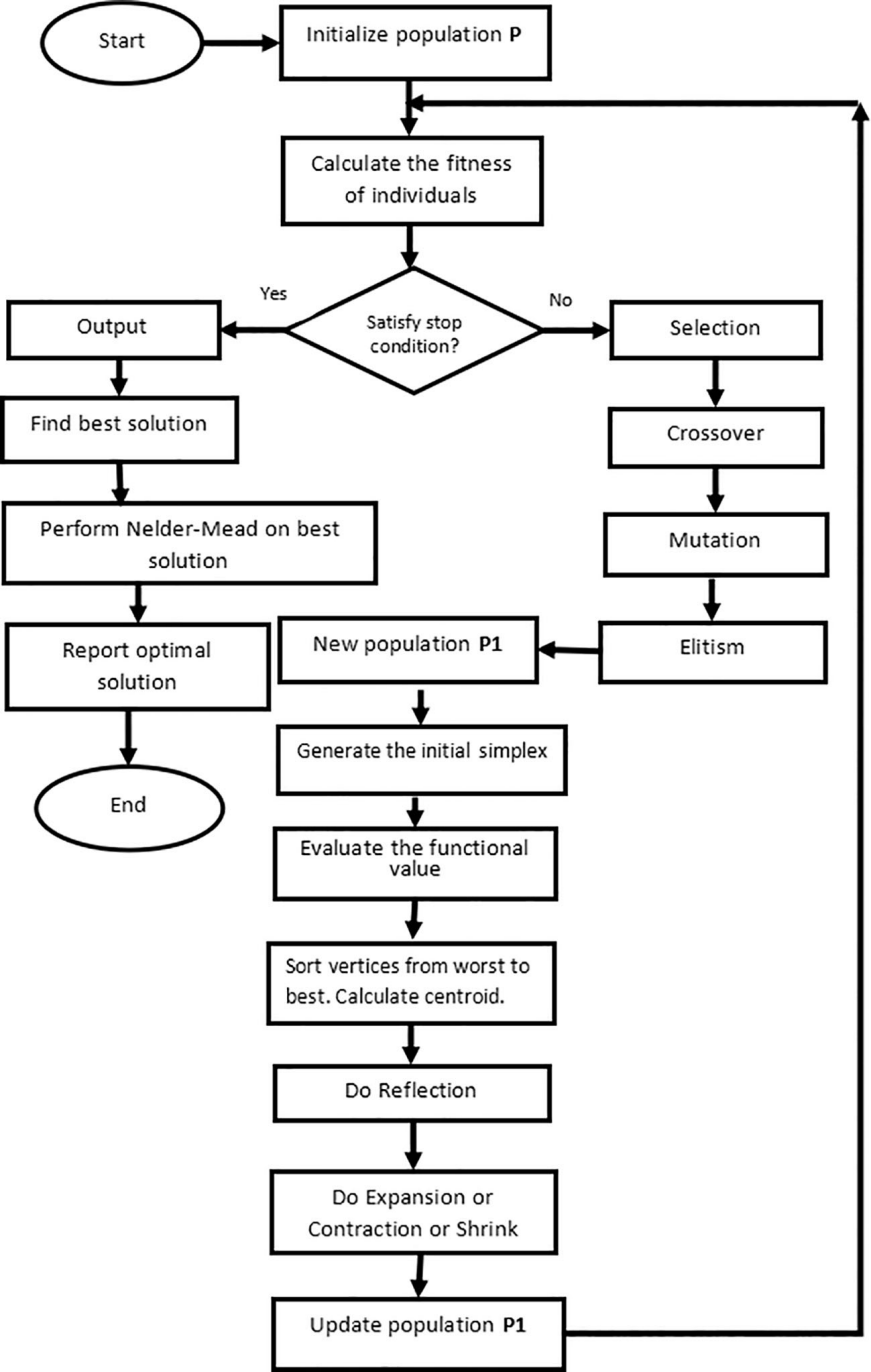
Flow-chart of GANMA.

The detailed flow diagram of the proposed algorithm is shown in Figure 1.

### 3.3 Hybrid Genetic Algorithm with Nelder-Mead (GA-NMA)

Here is an algorithm that combines a Genetic Algorithm (GA) with the Nelder-Mead Algorithm (NMA), where the GA first locates the interval containing the global minimum, and NMA refines the solution:

I. **Initialize** GA Population Generate an initial population of candidate solutions. Define the fitness function for evaluation.
II. **Apply GA Operations Selection:** Choose individuals based on their fitness. **Crossover:** Combine pairs of individuals to produce offspring. **Mutation:** Introduce random variations to maintain diversity.
III. **Evaluate the Population** Compute the fitness of each individual.
IV. **Iterate GA Process** Repeat the selection, crossover, mutation, and evaluation steps for a predefined number of generations or until convergence criteria are met.
V. **Identify Promising Interval** Extract the best individual(s) from the final GA population. Define the search interval around the best individual to locate the global minimum.
VI. **Initialize NMA** Use the best solution from GA as the starting point for NMA. Construct an initial simplex based on the chosen starting point.
VII. **Apply NMA** Iteratively refine the solution using simplex operations (reflection, expansion, contraction, and shrinkage). Stop when the termination criteria (e.g., small simplex size or convergence) are met.
VIII. **Output Final Solution** Return the refined solution as the global minimum estimate.

The pseudo-code for the hybridization of the GA and Nelder-Mead simplex algorithm is presented in Algorithm 2.

Algorithm 2. **Hybrid GA-NMA Algorithm.**

1: **Initialize GA Population:** Generate an initial population of candidate solutions.

Define the fitness function for evaluation.

2: **while** stopping criteria are not met **do**

3: **Selection:** Choose individuals based on their fitness.

4: **Crossover:** Combine pairs of individuals to produce offspring.

5: **Mutation:** Introduce random variations to maintain diversity.

6: **Evaluate Population:** Compute the fitness of each individual.

7: **end while**

8: **Identify Promising Interval:** Extract the best individual(s) from the final GA population and define the search interval around the best individual.

9: **Initialize NMA:** Use the best solution from GA as the starting point. Construct an initial simplex based on this starting point.

10: **repeat**

11: **Apply Simplex Operations:** Perform reflection, expansion, contraction, and shrinkage steps.

12: **until** termination criteria are met (e.g., small simplex size or convergence)

13: **Output Final Solution:** Return the refined solution as the global minimum estimate.

### 3.4 Benchmark functions

This study analyzes 15 benchmark test functions for simulation tests to fully investigate the feasibility as well as the effectiveness of GANMA. The 15 benchmark test functions (denoted as *f*_1_ to *f*_15_), cover different types. The unimodal functions (A function with a single peak or trough, making it straightforward to locate the global optimum) *f*_1_ through *f*_4_ are included in the first kind. Multimodal functions (A function with multiple peaks or troughs, presenting challenges in finding the global optimum due to local optima) *f*_5_ through *f*_9_ are included in the second category. Shifted unimodal and multimodal functions (Shifted Unimodal Function: an unimodal function whose peak or trough is relocated to a different position in the search space. Shifted Multimodal Function: A multimodal function with its peaks or troughs displaced, adding complexity by altering the relative positions of local and global optima), *f*_10_ - *f*_15_, are included in the third category. Table 1 displays the expressions, ranges, and global minimum values of the 15 test functions. The function’s dimensions (n) are 10, 20, and 30, in that order.

**Table 1.** Benchmark test functions.

No	Function name	Formulation	Range	$f_{\min}$
$f_{1}$	Sphere	$\sum_{i=1}^{n}{x_{i}}^{2}$	[-100,100]	0
$f_{2}$	Rosenbrock	$\sum_{i=1}^{n-1}\left(100{\left(x_{i+1}-{x_{i}}^{2}\right)}^{2}+{\left(1-x_{i}\right)}^{2}\right)$	[-2,2]	0
$f_{3}$	Rotated high-conditioned elliptic	$\sum_{i=1}^{n}{\left(10^{6}\right)}^{\frac{i-1}{n-1}}{x_{i}}^{2}$	[-100,100]	0
$f_{4}$	Ellipsoid	$\sum_{i=1}^{n}{\left(i.x_{i}\right)}^{2}$	[-100,100]	0
$f_{5}$	Ackley	$-20\exp\left(-0.2\sqrt{\frac{1}{n}\sum_{i=1}^{n}{x_{i}}^{2}}\right)-\exp\left(\;\frac{1}{n}\sum_{i=1}^{n}\cos\left(2\pi x_{i}\right)\right)+20+e$	[-30,30]	0
$f_{6}$	Griewank	$\sum_{i=1}^{n}{x_{i}}^{2}/4000-\prod_{i=1}^{n}\cos\left(\frac{x_{i}}{\sqrt{i}}\right)+1$	[-600,600]	0
$f_{7}$	Rastrigin	$10n+\sum_{i=1}^{n}\left({x_{i}}^{2}-10\cos\left(2\pi x_{i}\right)\right)$	[-5,5]	0
$f_{8}$	Schwefel	$418.9829n-\sum_{i=1}^{n}x_{i}\;\sin\left(\sqrt{\left|x_{i}\right|}\right)$	[-500,500]	0
$f_{9}$	Schwefel1.2	$\sum_{i=1}^{n}|x_{i}|$	[-5,5]	0
$f_{10}$	Shifted Sphere	$\sum_{i=1}^{n}{\left(x_{i}-{Ο}_{i}\right)}^{2}$	[-100,100]	0
$f_{11}$	Shifted Elliptic	$\sum_{i=1}^{n}{\left(10^{6}\right)}^{\frac{i-1}{n-1}}\;{\left(x_{i}-{Ο}_{i}\right)}^{2}$	[-100,100]	0
$f_{12}$	Shifted Rosenbrock	$\;\sum_{i=1}^{n-1}\left(100{\left(x_{i+1}-{x_{i}}^{2}\right)}^{2}+{\left(x_{i}-{Ο}_{i}\right)}^{2}\right)$	[-30,30]	0
$f_{13}$	Shifted Rastrigin	$\;\sum_{i=1}^{n}\left[{\left(x_{i}-{Ο}_{i}\right)}^{2}-10\cos\left(2\pi \left(x_{i}-{Ο}_{i}\right)\right)\right]$	[-5,5]	0
$f_{14}$	Shifted Griewank	$\frac{1}{4000}\sum_{i=1}^{n}{\left(x_{i}-{Ο}_{i}\right)}^{2}-\prod_{i=1}^{n}\cos\left(\frac{x_{i}-{Ο}_{i}}{\sqrt{i}}\right)+1$	[-600,600]	0
$f_{15}$	Shifted Ackley	$-20\exp\left(-0.2\sqrt{\frac{1}{n}\sum_{i=1}^{n}{\left(x_{i}-{Ο}_{i}\right)}^{2}}\right)-\exp\left(\frac{1}{n}\sum_{i=1}^{n}\cos\left(2\pi \left(x_{i}-{Ο}_{i}\right)\right)\right)+20+e$	[-30,30]	0

Ο = ( ${Ο}_{1},{Ο}_{2},..,{Ο}_{n}$ ) is the shift vector. n = dimension.

## 4. Parameter setup

### 4.1 Genetic Algorithm (GA) Parameters

For problem dimensions 10, 20, and 30, the Genetic Algorithm (GA) was executed for 300, 400, and 600 generations, respectively, starting with a population of 100 individuals. Eighty percent of the population had a one-point crossover, which translates to a crossover rate of 0.8. With a mutation rate of 0.05, random mutation was employed. With a tournament size of five, parents were selected by tournament selection, and the top 10 percent of each generation’s top performers were preserved through an elitism technique.

### 4.2 Nelder-Mead Algorithm (NM) Parameters

The Nelder-Mead (NM) algorithm was initialized using the solutions provided by the GA. Standard transformation coefficients were applied, including a reflection coefficient ( *α*) of 1, an expansion coefficient ( *γ*) of 1.5, and both contraction ( *ρ*) and shrinkage ( *σ*) coefficients set to 0.5. The step size was maintained at 1.0. The algorithm’s simplex shrinking process concluded when the convergence tolerance reached 10 ^*−*6^.

### 4.3 Hybrid Algorithm (GANMA) Settings

The hybrid process iterated through GA and NMA stages for 300, 400, and 600 generations for 10, 20, and 30 dimensions respectively. The stopping criteria were based on either the maximum number of iterations or fitness convergence, defined by a fitness tolerance of *ϵ* = 10 ^*−*5^, ensuring early detection of optimal solutions. Table 1 displays the expressions, dimensions, ranges, and global minimum values of the fifteen benchmark test functions (denoted as *f*_1_ - *f*_15_).

### 4.4 Computational Environment

The experiments were conducted in a consistent computational environment using Python 3.11. The hybrid GANMA algorithm was implemented from scratch, leveraging key Python libraries. NumPy handled arrays and matrix operations, Matplotlib was used for visualizing convergence and results, and SciPy supported NMA-based optimization. All tests were executed in a Jupyter Notebook environment to allow for easy experimentation and tuning. Each experiment was repeated 50 times to ensure statistical reliability.

Table 2 demonstrates how the performance of the GANMA, GA, and NM algorithms for dimensions (n) 10, 20, and 30 have been evaluated by comparing the mean value (Mean), standard deviation (Std), and best value (Best) of the final solutions for each benchmark function throughout 30 trials. The algorithm achieves the best optimization performance with the least standard deviation, optimal value, and average value closer to the theoretical ideal value. Any value less than 10 ^*−*6^ in terms of mean, standard deviation, and best value will be regarded as zero. The ideal experimental outcomes are truncated.

**Table 2.** For n = 10, 20, and 30, the best, mean, and standard deviation of the GANMA, GA, and NM solutions.

*Fun*	*Method*	*n = 10 Best*	*Mean*	*Std*	*n = 20 Best*	*Mean*	*Std*	*n = 30 Best*	*Mean*	*Std*
*f*_1_	GANMA	3.92E-278	1.01E-235	0.00E+00	4.62E-52	1.10E-45	3.30E-45	1.09E-20	4.10E-17	1.10E-16
	GA-NMA	1.24E-201	1.95E-184	0.00E+00	4.24E-49	2.97E-43	6.64E-43	3.85E-14	1.53E-12	4.03E-12
	GA	2.96E-01	8.90E-01	1.39E-02	1.24E+00	3.03E+00	1.45E+00	1.5E+02	2.8E+02	1.0E+02
	NM	3.96E-183	6.63E-166	0.00E+00	5.39E-37	2.03E-32	5.48E-32	2.51E-18	7.47E-13	1.11E-12
*f*_2_	GANMA	5.22E-29	1.44E-28	7.60E-29	3.69E-27	6.94E-26	9.75E-26	6.49E-18	1.80E-14	1.85E-14
	GA-NMA	6.18E29	4.06E-27	6.02E-27	2.22E-26	8.78E-25	2.11E-24	1.02E-24	8.93E-24	1.49E-23
	GA	5.67E+00	6.90E+00	8.78E-01	1.64E+01	1.74E+01	4.81E-01	2.62E+01	4.07E+01	1.63E+01
	NM	5.91E-29	2.38E-27	2.31E-27	1.89E-26	2.30E-25	3.20E-25	9.10E-16	1.43E-11	2.60E-11
*f*_3_	GANMA	7.88E-243	4.78E-232	0.00E+00	6.93E-49	3.97E-45	1.12E-44	1.58E-16	7.55E-14	1.39E-13
	GA-NMA	1.17E-195	1.91E-179	0.00E+00	1.00E-49	7.69E-46	1.57E-45	1.76E-27	2.32E-25	4.67E-25
	GA	1.36E+03	5.08E+04	1.03E+04	9.85E+03	5.22E+04	2.93E+04	7.98E+03	2.41E+04	1.00E+04
	NM	9.32E-186	4.62E-165	0.00E+00	1.16E+01	1.44E+04	1.14E+04	8.87E+04	9.68E+05	3.88E+05
*f*_4_	GANMA	7.43E-250	6.45E-235	0.00E+00	2.09E-50	5.42E-44	1.57E-43	1.44E-16	3.78E-15	4.98E-15
	GA-NMA	9.26E-199	1.10E-186	0.00E+00	4.03E-49	5.47E-45	8.47E-45	9.03E-27	7.78E-23	2.02E-22
	GA	8.60E-02	5.56E-01	3.81E-01	1.11E+00	7.66E+00	6.05E+00	1.38E+04	2.15E+04	6.00E+03
	NM	6.30E-184	4.58E-171	0.00E+00	4.59E-39	1.00E-32	2.53E-32	2.31E-15	3.89E-11	1.05E-10
*f*_5_	GANMA	2.88E-14	3.18E-13	1.62E-13	4.01E-13	1.11E-11	1.45E-11	7.09E-10	4.39E-11	1.29E-11
	GA-NMA	2.17E-14	1.22E-01	2.09E-01	3.77E-13	1.22E-01	8.16E-01	1.52E-11	2.70E+00	1.58E+00
	GA	7.17E-02	9.74E-01	6.37E-01	4.40E-01	8.93E-01	2.78E-01	2.23E+00	3.21E+00	5.99E-01
	NM	1.86E+01	1.93E+01	3.00E-01	1.90E+01	1.94E+01	2.17E-01	1.84E+01	1.89E+01	2.53E-01
*f*_6_	GANMA	7.13E-02	1.46E-01	5.22E-02	1.66E-01	2.52E-01	2.17E-01	9.41E-01	1.66E+00	5.25E-01
	GA-NMA	1.23E-02	1.40E-01	9.68E-02	5.21E-01	2.50E-01	1.74E-01	1.01E+00	2.133E+00	2.20E+00
	GA	1.86E-01	3.56E-01	1.11E-01	1.00E+00	1.02E+00	1.39E-02	2.47E+00	3.53E+00	9.43E-01
	NM	1.42E+00	2.57E+01	1.34E+01	7.39E-02	5.71E+00	7.57E+00	9.09E-13	2.02E+00	1.65E+00
*f*_7_	GANMA	1.96E-05	2.00E-04	2.00E-03	4.00E-04	8.90E-03	7.70E-03	1.19E+00	2.84E+00	1.16E+00
	GA-NMA	5.65E-02	1.75E-01	1.24E-01	6.95E-01	1.18E+00	3.71E-01	8.78E+00	1.36E+01	3.28E+00
	GA	6.29E-05	2.20E-02	3.40E-02	1.00E-03	1.46E-02	1.56E-02	2.47E+00	3.58E+00	1.80E+00
	NM	8.30E+01	1.06E+02	1.74E+01	1.19E+02	1.71E+02	5.18E+01	1.88E+02	2.89E+02	5.49E+01
*f*_8_	GANMA	-1.97E+01	-1.57E+01	7.89E+00	-3.94E+01	-1.18E+01	1.57E+01	-9.86E+01	-4.73E+01	2.95E+01
	GA-NMA	1.27E-04	1.27E-04	3.03E-10	2.54E-04	2.54E-04	3.66E-08	3.81E-04	3.81E-04	2.56E-08
	GA	3.45E-02	7.88E-02	4.12E-02	6.72E+00	8.25E+00	1.10E+00	1.09E+02	1.54E+02	3.0E+01
	NM	6.31E+02	1.37E+04	3.81E+02	2.32E+03	2.84E+03	3.82E+02	4.06E+03	5.23E+03	1.04E+03
*f*_9_	GANMA	5.11E-09	4.88E-06	4.95E-06	4.77E-06	1.56E-05	8.25E-06	8.83E-06	2.30E-05	1.05E-05
	GA-NMA	1.86E-05	1.96E-03	1.80E-03	6.16E-04	4.73E-03	3.95E-03	1.14E-02	6.55E-02	6.38E-02
	GA	8.00E-05	8.50E-02	7.60E-02	2.56E-01	5.47E-01	1.80E-01	2.38E+00	3.89E+00	9.46E-01
	NM	6.98E-01	1.21E+00	4.94E-01	1.87E+00	6.43E+00	5.55E+00	2.25E+00	7.34E+00	4.84E+00
*f*_10_	GANMA	2.46E-31	8.02E-31	5.62E-31	1.01E-29	4.13E-29	2.00E-29	9.12E-16	1.05E-16	3.02E-16
	GA-NMA	1.23E-31	3.50E-29	7.57E-29	6.69E-29	9.97E-28	1.63E-27	1.75E-27	6.74E-25	1.64E-24
	GA	1.18E-05	3.14E-02	6.74E-02	9.86E+01	1.37E+02	3.49E+01	1.89E+03	3.65E+03	1.11E+03
	NM	1.78E-30	7.60E-30	6.09E-30	5.08E-29	1.96E-28	1.51E-28	1.84E-15	1.13E-09	3.00E-09
*f*_11_	GANMA	2.90E-26	5.12E-25	5.85E-24	3.16E-24	7.45E-24	3.25E-24	1.46E-15	8.71E-14	1.69E-13
	GA-NMA	4.39E-26	1.00E-24	2.12E-24	7.13E-24	4.67E-23	4.92E-23	3.03E-23	1.09E-21	1.60E-21
	GA	1.01E+00	3.49E+02	5.78E+02	1.00E+05	1.62E+05	8.76E+03	5.63E+05	1.11E+06	3.41E+06
	NM	3.68E-26	7.21E-25	1.13E-24	2.93E+03	1.98E+04	1.55E+04	1.29E+04	2.49E+05	1.51E+05
*f*_12_	GANMA	8.13E-29	6.12E-28	4.43E-28	1.59E-26	1.45E-25	1.92E-25	1.14E-18	2.20E-15	4.29E-15
	GA-NMA	9.81E-28	4.29E+00	1.87E+01	5.26E-26	5.97E-01	1.42E+00	2.37E-24	1.59E+00	1.95E+00
	GA	9.03E+00	4.87E+01	3.27E+01	1.46E+02	2.11E+02	7.77E+01	7.88E+03	2.52E+04	1.12E+04
	NM	1.15E-27	1.59E+00	1.95E+00	3.40E-25	7.97E-01	1.59E+00	2.94E-14	3.18E+00	1.59E+00
*f*_13_	GANMA	1.44E-02	2.13E-02	1.02E-02	2.82E+00	3.74E+00	1.71E+00	9.01E+00	1.05E+01	1.43E+00
	GA-NMA	3.61E-02	1.73E-01	7.70E-02	5.12E-01	9.97E-01	3.23E-01	8.86E+00	1.52E+01	2.74E+00
	GA	4.60E-02	1.49E-01	1.19E-01	8.14E+00	9.42E+00	1.12E+00	1.58E+01	1.98E+01	3.71E+00
	NM	6.40E+01	8.76E+01	2.60E+01	1.70E+02	2.07E+02	2.36E+01	2.08E+02	3.03E+02	5.42E+01
*f*_14_	GANMA	1.42E-02	4.42E-02	2.85E-02	8.11E-02	1.21E-01	7.64E-02	1.21E-13	4.90E-02	4.10E-02
	GA-NMA	2.33E-15	1.01E-01	7.00E-02	6.77E-15	9.34E-03	1.28E-02	1.57E-13	1.26E-02	1.29E-02
	GA	2.10E-01	4.81E-01	1.79E-01	1.83E+00	2.32E+00	2.97E-01	2.73E+00	3.75E+00	5.10E-01
	NM	5.36E+00	3.23E+01	4.09E+01	2.40E-01	1.34E+01	9.05E+00	7.30E-02	7.99E-01	9.70E-01
*f*_15_	GANMA	5.01E-14	1.98E-12	3.10E-12	3.25E-12	1.43E+00	1.77E+01	2.22E+00	4.27E+00	3.10E+00
	GA-NMA	3.24E-14	8.55E-02	1.31E-01	1.82E-12	4.49E+01	1.20E+01	1.48E-11	2.45E+02	1.66E+02
	GA	1.10E-01	4.17E-01	1.20E-01	2.91E+00	3.85E+00	6.97E-01	9.29E+00	1.04E+01	1.23E+00
	NM	1.85E+01	1.89E+01	2.95E-01	1.89E+01	1.93E+01	2.33E-01	1.92E+01	1.93E+01	1.31E-01

## 5. Experimental results and analysis

The statistical results of GANMA’s performance on 15 benchmark functions with dimensions (n) of 10, 20, and 30 are shown in Table 2. It also contains the final solutions’ best (Best), mean (Mean), and standard deviation (Std) across a 30-run period for each benchmark function. All benchmark functions for unimodal functions ( *f*_1_ - *f*_4_) have been solved in all three dimensions (10, 20, and 30). For the multimodal functions ( *f*_5_ – *f*_9_), the solutions for *f*_5_ and *f*_9_ occur in 10, 20, and 30 dimensions, whereas the solutions for *f*_6_ and *f*_7_ in 10 and 20 dimensions are almost optimal. The standard deviation range is 1 *.*62 *E* − 13 ∼ 7 *.*89 *E* + 00, 1 *.*45 *E* − 11 ∼ 1 *.*57 *E* + 01, and 1 *.*29 *E* − 11 ∼ 2 *.*95 *E* + 01, respectively, while the mean value’s variations range in the 10, 20, and 30 dimensions is 3 *.*18 *E* − 13 ∼ 1 *.*46 *E* − 01, 1 *.*11 *E* − 11 ∼ 2 *.*52 *E* − 01, and 4 *.*39 *E* − 11 ∼ 2 *.*84 *E* + 00.

Six shifted test functions have been chosen for this study to validate the performance of GANMA: three shifted multimodal test functions, denoted as *f*_13_ to *f*_15_; and three shifted unimodal test functions, denoted as *f*_10_ to *f*_12_, Sphere, Elliptic, and Rosenbrock. On functions *f*_10_, *f*_11_, *f*_12_ (in 10, 20, and 30), and *f*_15_ (in 10), GANMA achieved optimum solutions; on functions *f*_13_ (in 10) and *f*_14_ (in 10, 20, and 30), the solutions are nearly optimal. Even while GA is outperformed by the solutions of *f*_13_ and *f*_15_ (in 20 and 30) in GANMA, the solutions are still far from the optimal ones. Furthermore, the Std that GANMA found on five test functions is not too high, suggesting that GANMA’s performance on shifted test functions is steady.

Therefore, for all unimodal functions (in 10, 20, and 30 dimensions), GANMA can obtain the global optimum. GANMA can identify outcomes with negligible deviations from the global optimal value for multimodal functions. Except for *f*_5_ and *f*_9_ in dimensions 10, 20, and 30, the results of *f*_6_, and *f*_7_ in dimensions 10 and 20 are quite near to the optimal value. The outcomes produced by GANMA algorithms for shifted unimodal and multimodal functions are optimal or extremely near-optimal in all functions for all three dimensions, except *f*_13_ and *f*_15_ (in 20 and 30). The benefits of the GANMA algorithm include excellent robustness, high convergence accuracy, and steady performance in all scenarios, whether they involve multimodal functions, unimodal functions, or shifting unimodal and multimodal functions. This is shown in Table 2 under the various numbers of iterations for the corresponding dimensions, which are 300, 400, and 600 for the dimensions 10, 20, and 30, respectively.

GANMA consistently outperforms GA-NMA, shown in Table 2, across various function categories, particularly in unimodal functions. For example, in *f*_1_ and *f*_4_, GANMA achieves near-zero fitness values across all dimensions, demonstrating its ability to efficiently refine solutions in smooth landscapes. Its lower standard deviations further indicate robust and stable convergence compared to GA-NMA, which struggles to maintain similar precision. In multimodal functions like *f*_8_, GANMA excels by navigating complex landscapes with multiple local optima, achieving superior results in higher dimensions (e.g., n = 30). Its hybrid structure effectively balances global exploration and local exploitation, reducing the risk of premature convergence. In contrast, GA-NMA often stagnates in local optima due to less dynamic exploration capabilities, leading to higher fitness values and greater variability.

For shifted unimodal functions such as *f*_10_, GANMA demonstrates its adaptability by achieving significantly lower best and mean fitness values, overcoming challenges introduced by displaced optima. Similarly, in shifted multimodal functions like *f*_13_ and *f*_15_, GANMA showcases its robustness by effectively handling complex, displaced landscapes. GANMA achieves accurate and dependable convergence by fine-tuning solutions even in challenging environments by utilizing Nelder-Mead for local refining. GA-NMA, however, struggles with the combined challenges of shifting and multimodal complexity, resulting in higher fitness values and inconsistent performance. Overall, GANMA’s adaptability and superior optimization capabilities make it a robust choice for diverse and challenging optimization problems.

**Figure 2.**
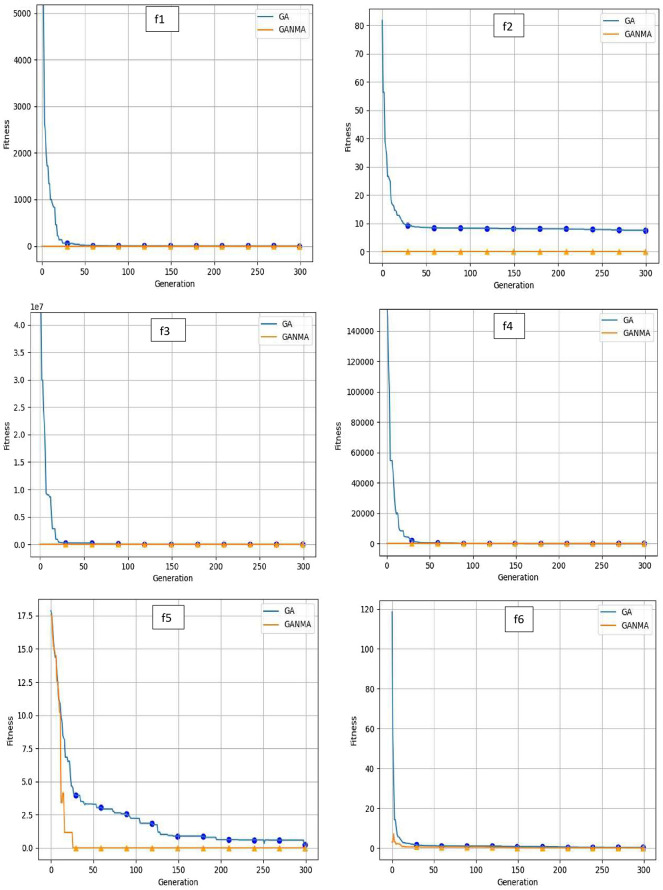

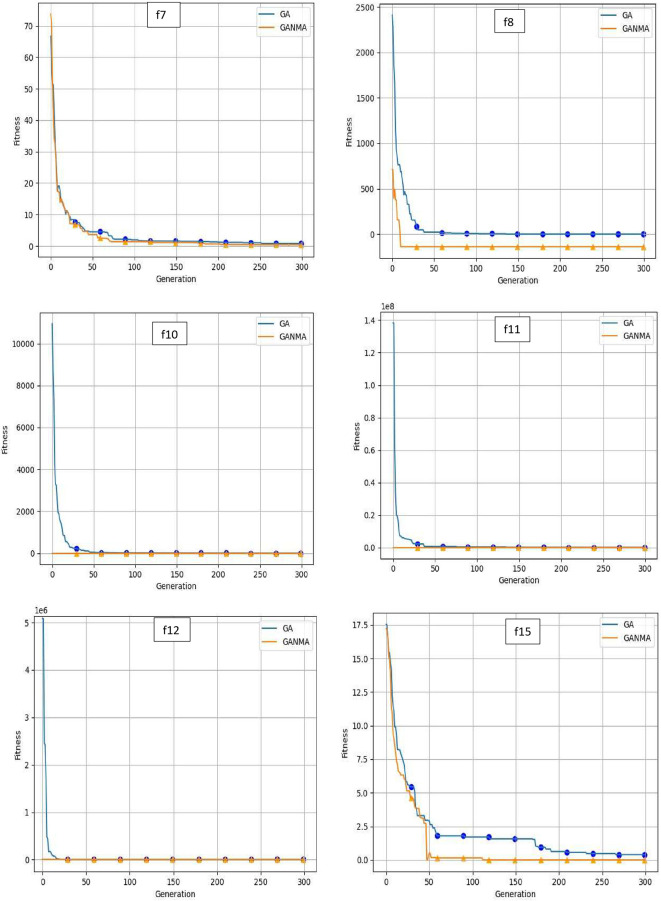
Convergence graphs of functions for n = 10.

**Figure 3.**
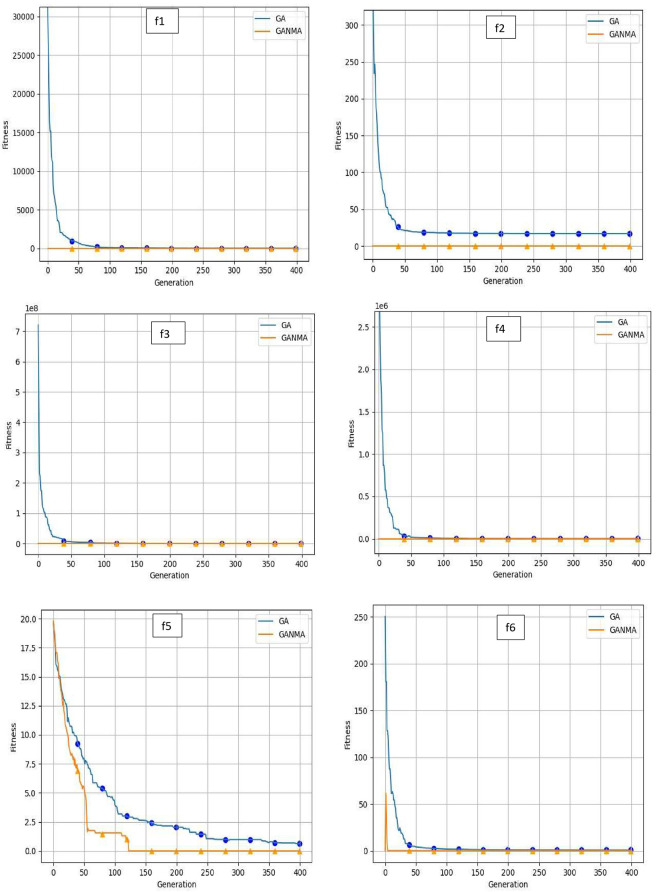

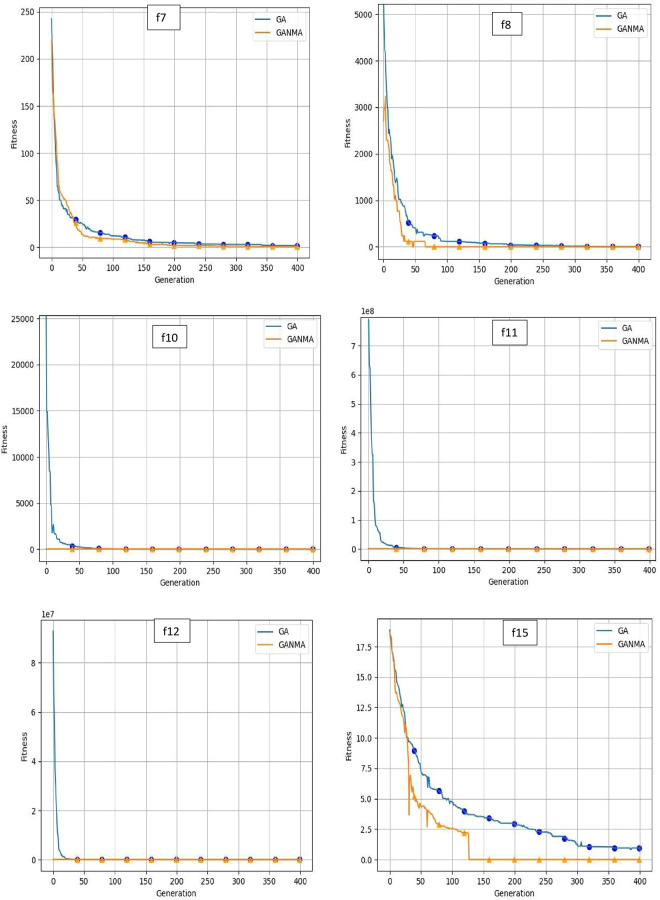
Convergence graphs of functions for n = 20.

**Figure 4.**
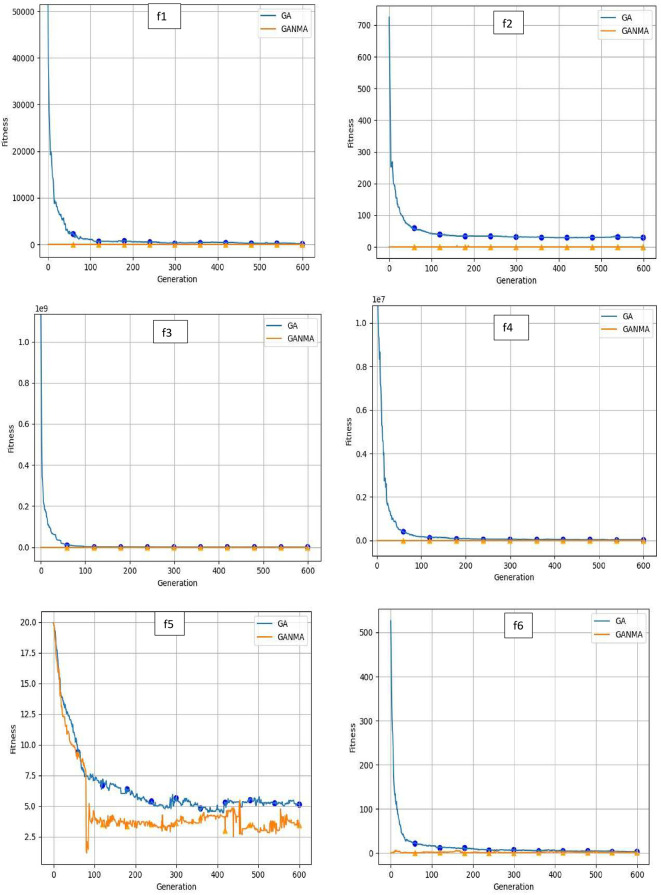

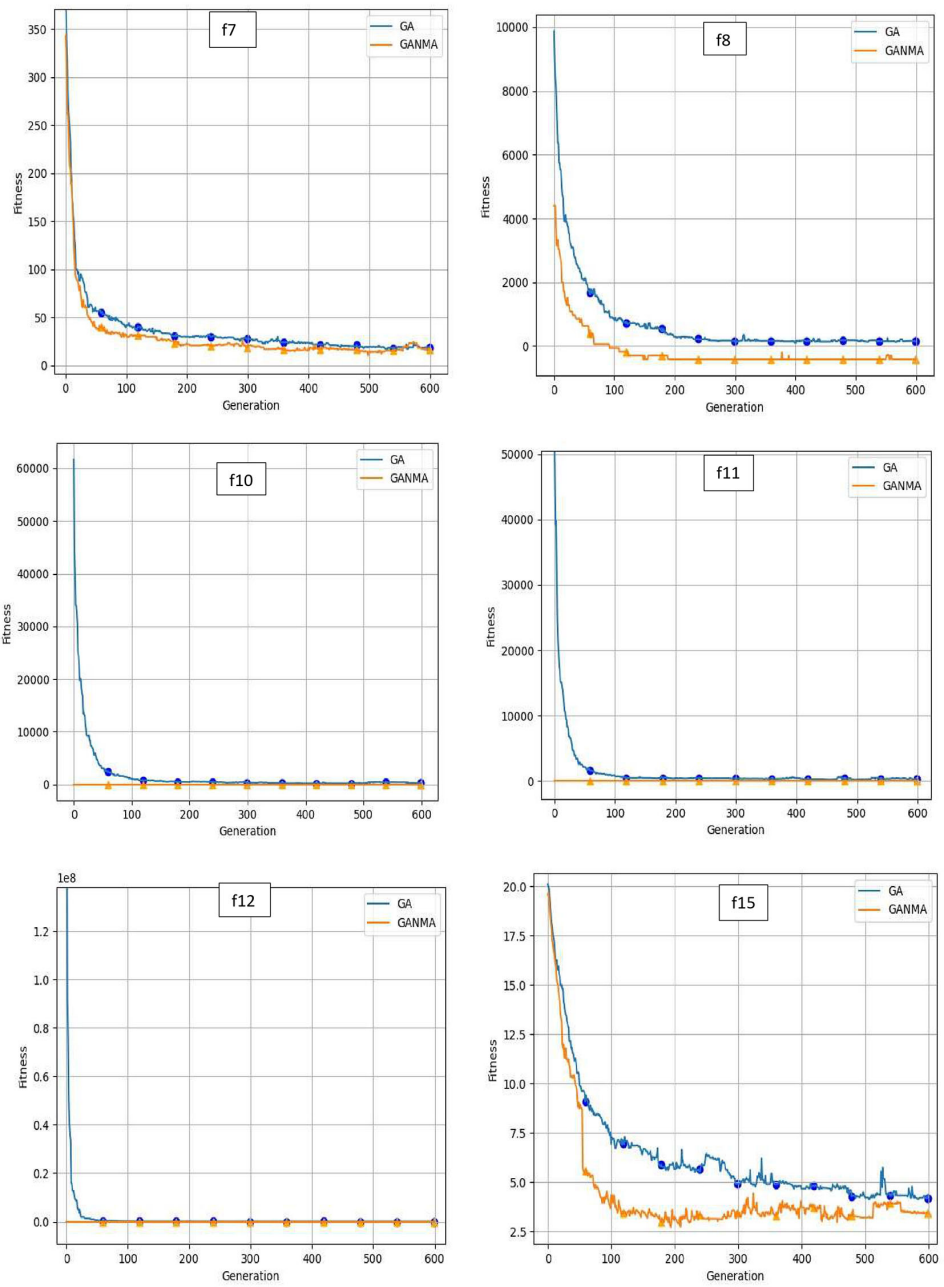
Convergence graphs of functions for n = 30.

To help further investigate the evolutionary behavior of various methods, the convergence curves of GANMA and GA for a few chosen benchmark functions are displayed in Figure 2, Figure 3, and Figure 4 for dimensions (n) = 10, 20, and 30, respectively. These graphs demonstrate the convergence behavior of methods that can help to analyze the evolutionary behavior of various algorithms. The y- and x-axes, respectively, represent the values of the fitness function and the number of iterations. The blue solid line shows the genetic algorithm (GA), while the suggested method GANMA is shown by the solid orange line.

Until the ideal solution is discovered, GA shows a decreasing trend for unimodal functions like *f*_1_, *f*_2_, *f*_3_, and *f*_4_. In contrast, GANMA presents a straight line for all three dimensions (n = 10, 20, and 30). Similar to this, for multimodal functions other than *f*_5_ and *f*_7_ (in 30), there is a greater similarity between the global optimum solution and the GANMA optimal solution in *f*_5_, *f*_7_ (in 10, and 20), and *f*_6_, *f*_8_ (in 10, 20, and 30). As a result, of these two algorithms, the lowest optimum solution and the fastest rate of convergence are found through GANMA. The curves for shifted functions, except *f*_15_ (in 20 and 30), demonstrate how well the proposed method was able to obtain the ideal solution for other functions like *f*_10_, *f*_11_, and *f*_12_ (in 10, 20, and 30).

The dynamic character of the algorithm during its exploitation phases is reflected in the zigzag behavior shown in the figures. The main cause of this pattern is the optimization algorithms’ natural localized refinement processes. Local search methods cause local search methods cause local search methods cause these variations, such as the Nelder-Mead algorithm, which concentrates on enhancing solutions within a limited area of the search space. Furthermore, mutation processes in Genetic Algorithms (GA) add variation by slightly altering individual solutions. The observed zigzag patterns might result from these alterations, which can lead to brief departures from a smooth convergence trajectory. Even while these variations can seem erratic, they highlight how the exploration and exploitation stages actively interact, demonstrating the algorithm’s attempts to improve solutions and converge to the best result. Out of these two methods, GANMA yields the lowest optimum solution and has the fastest convergence rate than GA’s in both the multimodal and shifted functions.

It is demonstrated by analyzing the convergence curve and experimental findings that GANMA typically exhibits remarkable performance on the 15 test functions, with a fraction of proper convergence to the global optimal solution that is close to 90%. In terms of exploration and exploitation, GANMA performs better than GA and the NM algorithm. Consequently, GANMA achieves lower fitness values, less variability, and more steady convergence than GA-NMA, GA, and NM. GANMA is a flexible and dependable hybrid algorithm because of its capacity to adjust to optimization problems ranging from simple unimodal functions to intricate shifting multimodal ones. This robustness highlights its advantage in solving diverse real-world optimization problems.

## 6. Application of proposed algorithm (GANMA) for Weibull-Parameter Estimation

The Weibull distribution is a probability distribution that is often used in reliability and survival research. Weibull et al. ^23^ had shown that the Weibull distribution fit many different datasets and offered satisfactory results, even for small samples. The Weibull distribution, known for its flexibility in modeling various failure and survival scenarios, is defined by two parameters: the shape ( *β*) and scale ( *η*) parameters. In some cases, a location ( *α*) parameter is added to create a three-parameter Weibull distribution, allowing for greater flexibility in fitting data with location shifts. The three-parameter probability density function (pdf ) will have only two parameters ^24^ when the location parameter ( *α*) is equal to zero. Due to the fact that no failure may occur before or after the time is zero, the two Weibull parameters are frequently utilized in failure analysis. ^25^

Weibull parameter estimation employs a variety of methods. Method of Moments (MOM), the maximum likelihood (ML) approach, and modified maximum likelihood (MML) methods were all used by Seguro and Lambert. ^26^ They discovered that the time series data sets are more suited for the ML approach. They advised utilizing the MML technique for data sets that were formatted as frequency distributions. The least squares approach, the ML method, and the MML method were contrasted by Akgül et al. ^27^ ML was shown to be the most effective approach overall, but they also noted that MML and ML are equally effective for big data sets, despite MML’s lower computational complexity. The ML technique was used in the studies of Kollu et al. ^28^ and Akpnar and Akpnar ^29^ to estimate the Weibull parameters. Teimouri et al. ^30^ investigated the MoM using their proposed L-moment estimator, the ML approach, the logarithmic moment method, and the percentile method. They discovered that the ML method and their suggested approach are the most effective estimators. The power density approach was proposed by Akda and Dinler. ^31^ They concluded that it outperformed popular techniques like MoM and ML techniques. After evaluating five different methods for approximating the Weibull distribution, Saleh et al. ^32^ recommended the mean wind speed methodology and the ML method. Azad and colleagues ^33^ discovered that the MoM and ML techniques were more effective than other approaches.

Considering the Weibull distribution has a nonlinear log-likelihood function and is compatible with numerical optimization techniques like Newton-Raphson (NR) and Nelder-Mead (NM), previous studies have often used MLE approaches for parameterizing the Weibull distribution. ^34, 35^ However, the effectiveness of these iterative methods heavily relies on the initial value chosen. ^36^ In a departure from traditional approaches, this study employs Genetic Algorithms (GAs) as a heuristic search method, considering a set of solutions within the search space rather than individual points, to address the initial value problem in Weibull parameter Maximum Likelihood Estimation. ^37, 38^ GAs have been successfully applied in various optimization contexts, ranging from optimizing mixing parameters for high-performance concrete to signal control optimization. ^39^ Parameterization of distributions such as the skew-normal distribution, ^40^ nonlinear regression, ^41^ and negative binomial gamma mixed distribution ^42^ have all been applied in previous works.

Notably, Thomas et al. ^43^ pioneered the use of GA for Weibull distribution parameter estimation in the context of breakdown periods of insulating fluid data, achieving performance comparable to traditional methods based on maximizing the log-likelihood function. Furthermore, hybrid approaches combining GA with other methods, such as the improved Nelder-Mead algorithm for controlling synchronous generator output voltage, ^36^ and memetic algorithms applied to parameter identification in electrical engineering, ^44^ underscore the versatility of heuristic and hybrid optimization techniques in solving complex problems.

### 6.1 Weibull distribution

A versatile continuous probability distribution, the Weibull distribution is frequently used in survival analysis and reliability engineering. It is characterized by its ability to model the distribution of time until an event occurs. Named after Wallodi Weibull, who described it in the 1950s, the distribution is flexible and can take different shapes depending on its parameters. The shape parameter affects the structure of the Weibull distribution curve resulting in whether the distribution appears to be a Rayleigh distribution ( *β* = 2), an exponential distribution ( *β* = 1), or another shape. The scale parameter determines the distribution’s scale or size. Together, these factors enable the Weibull distribution to simulate a wide range of events with varying shapes and sizes.

The following is the Weibull two-parameter distribution’s probability density function (PDF):

$$f\left(x;\beta ,\eta \right)=\{\begin{array}{ll} \frac{\beta }{\eta }{\left(\frac{x}{\eta }\right)}^{\beta -1}e^{-{\left(x/\eta \right)}^{\beta }}, & x\geq 0\\ 0, & x<0 \end{array}$$ (6)

where:

• *x* is the random variable,
•
β is the shape parameter,
•
η is the scale parameter.

The following represents the Weibull distribution’s cumulative distribution function (or CDF):

$$F\left(x;\beta ,\eta \right)=\left\{ \begin{array}{ll} 1-e^{-{\left(x/\eta \right)}^{\beta }}, & x\geq 0\\ 0, & x<0 \end{array}\right.$$ (7)

**Figure 5.**
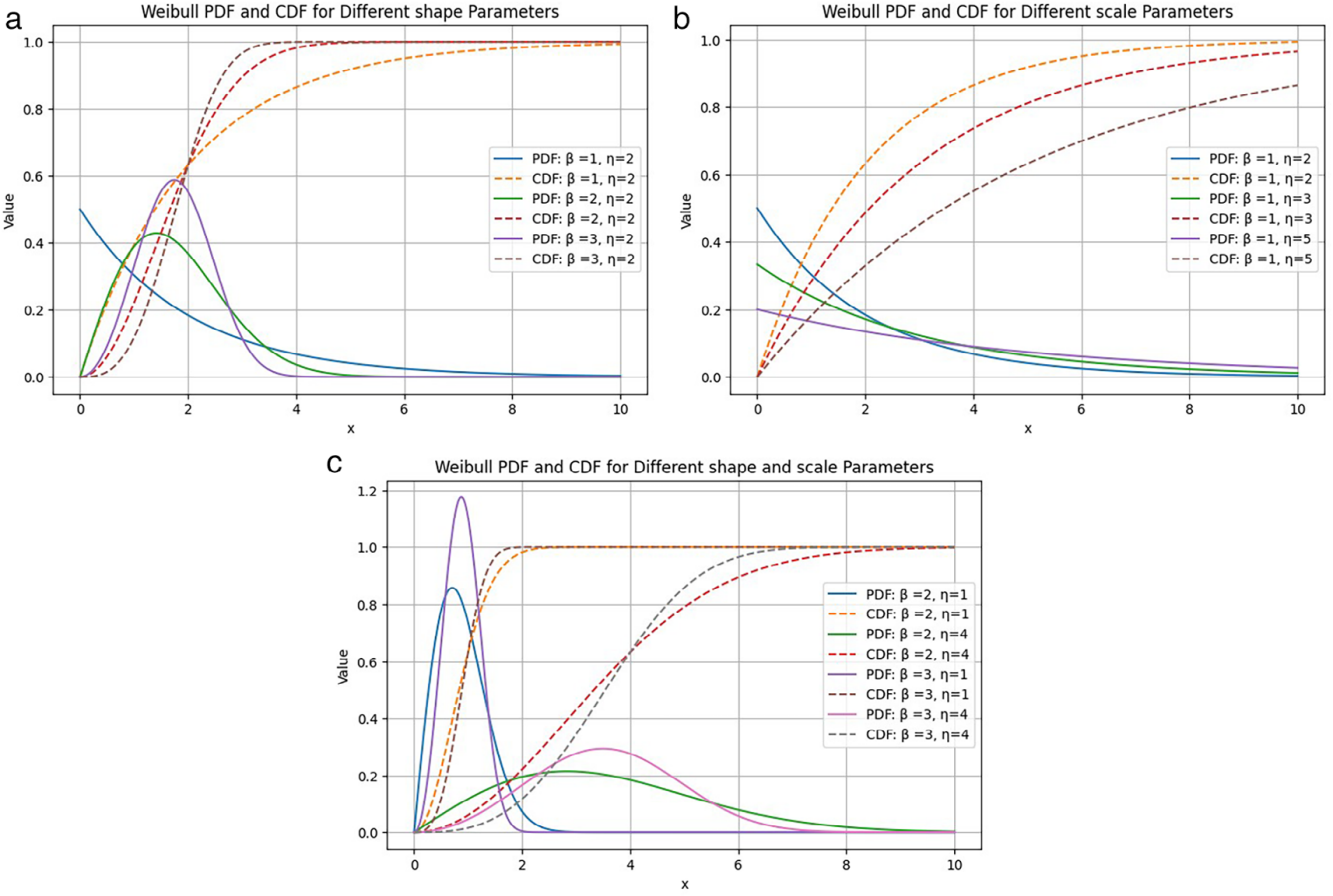
Different values of (a) shape parameter *β* and (b) scale parameter *η* are plotted in Weibull PDF (solid line) and CDF (dashed line) plots.

Probability density and cumulative distribution plots for some different parameter values are given in Figure 5.

Two-Parameter Weibull is Commonly applied in reliability engineering for modeling time until the failure of components. Whereas, Three-Parameter Weibull is Useful when considering scenarios where the event initiation may not be at zero, such as analyzing the time until an event occurs after a certain threshold.

## 7. Methods for estimating parameters

Estimating the parameters of the Weibull distribution poses a significant challenge due to the intricacies involved in utilizing sample data for accurate estimation. Parameter estimation involves the process of determining the distribution’s parameters using available sample data, aiming to derive optimal values that provide meaningful insights into the underlying data. Making incorrect parameter choices can lead to misleading results, underscoring the importance of analyzing and selecting appropriate estimation techniques for accurate modelling. Therefore, a thorough evaluation of estimation methods is essential to determine the most suitable approach for a given dataset and analysis context.

### 7.1 Maximum Likelihood Estimation (MLE)

The statistical method known as Maximum Likelihood Estimation (MLE) is used to estimate Weibull parameters by maximizing the likelihood function, which determines how well the distribution fits the observed data. MLE is known for its efficiency, but its optimization can be complex due to non-linear equations and numerical stability issues. The PDF of the Weibull distribution is given by Equation (6). Given a sample x _1_, x _2_, … x _n_ from a Weibull distribution, the likelihood function is given by:

$$L\left(\beta ,\eta \right)=\prod_{i=1}^{n}\;f\left(x_{i};\beta ,\eta \right)$$ (8)

where, *f* ( *x*; *β, η*) is the probability density function of the Weibull distribution.

The Weibull distribution’s log-likelihood function is as follows:

$$\mathrm{LnL}\left(\beta ,\eta \right)=\sum_{i=1}^{n}\;\left[\ln\left(\frac{\beta }{\eta }\right)+\left(\beta -1\right)\ln\left(\frac{x_{i}}{\eta }\right)-{\left(\frac{x_{i}}{\eta }\right)}^{\beta }\right]$$ (9)

$$\mathrm{LnL}\left(\beta ,\eta \right)=\mathrm{nln\beta}-\text{nβln}\eta +\left(\beta -1\right)\sum_{i=1}^{n}\;\ln x_{i}-\eta^{-\beta }\sum_{i=1}^{n}\;x_{i}\beta$$ (10)

The log-likelihood function is differentiated for *β* and *η*, the derivatives are set to zero, and the resultant system of equations is solved to get the MLE.

$$\frac{\partial L}{\partial \eta }=-\frac{\mathrm{n\beta}}{\eta }+\frac{\beta }{\eta^{\beta }+1}\sum_{i=1}^{n}\;x_{i}^{\beta }=0$$ (11)

$$\frac{\partial L}{\partial \beta }=\frac{n}{\beta }-\mathrm{nln\eta}-\frac{\sum_{i=1}^{n}\;x_{i}\beta -\ln\eta \sum_{i=1}^{n}\;x_{i}\beta }{\eta^{\beta }}+\sum_{i=1}^{n}\;\ln x_{i}=0$$ (12)

By eliminating *α* from the above equations and simplifying the equations we get,

$$\hat{\eta }={\left(\frac{1}{n}\sum_{i=1}^{n}\;x_{i}\beta \right)}^{\frac{1}{\beta }}$$ (13)

$$\frac{1}{\beta }-\frac{\sum_{i=1}^{n}\;x_{i}^{\beta }\ln x_{i}}{\sum_{i=1}^{n}\;x_{i}^{\beta }}+\frac{1}{n}\sum_{i=1}^{n}\;\ln x_{i}=0$$ (14)

Eqn. (13) may be used to calculate the estimate $\hat{\eta }$ . However, because of Eqn. (14) did not give an analytical solution, the estimate $\hat{\beta }$ must be calculated numerically. This is possible by using the optimization strategy. The Nelder-Mead, Newton Rapson, simulated annealing, or GA algorithms can all be used to solve the nonlinear function that the ML estimator of the shape parameter *β* contains. In this study, the suggested method, GA, and NM were all used to optimize the log-likelihood function. Nelder-Mead is a powerful algorithm that converges quickly, but its performance is dependent on the initial guess. As a result, we took into account the GA while maximizing the Weibull distribution’s loglikelihood function. Eqn. (10) is considered a fitness function for GA and NM methods.

Below the proposed method on MLE of Weibull Distribution has been described briefly.

***7.1.1 Proposed method***

***(Genetic and Nelder -Mead Algorithm (GANMA))***

To improve the precision and reliability of parameter estimation, we proposed a hybrid approach GANMA that integrates the GA and the NM method with MLE for two-parameter Weibull distributions. The GA aids in exploring the parameter space globally, generating diverse candidate solutions, while the NM fine-tunes these solutions through local search, aiming for optimal parameter estimates. To the best of our knowledge, this is the first instance where the GANMA is being utilized to estimate the Weibull distribution’s parameters.

The steps of the proposed method in this study are summarized as follows:

**Step 1:** Problem Formulation - We aim to find the MLE parameters *β* (shape) and *η* (scale) for a Weibull distribution.

**Step 2:** Genetic Algorithm (GA) Phase -

• Generate an initial population (P) of possible solutions. For the Weibull distribution, each solution indicates a collection of parameters ( *β*, *η*).
• Define the fitness function *f*( *β*, *η*) that measures the goodness of fit between the observed data and the Weibull distribution with the given parameters. A suitable fitness function could be the log-likelihood shown in Equation 10.
• Select individuals within the population according to their fitness by using a selection process (tournament selection). Higher fitness levels increase the probability of selection.
• Apply crossover operations (one-point crossover) to pairs of selected individuals to create new candidate solutions.
• Introduce small random changes (mutations) to the parameters of some individuals to add diversity to the population.

**Step 3:** Nelder-Mead Algorithm (NM) Phase -

• Take the best individual from the final population of the GA as an initial guess for the parameters ( *β*1, *η*1).
• Define the log-likelihood function L( *β*, *η*) for the Weibull distribution shown in Equation 5.
• To minimize the log-likelihood function and improve the parameter estimations (i.e., reflection, expansion, contraction, and shrinkage), apply the Nelder-Mead method.
• Repeat the iterations until convergence criteria are met (e.g., small changes in parameters or a maximum number of iterations).

**Step 4:** Repeat the selection, crossover, and mutation steps for several generations until convergence is met (i.e. end of GA phase).

**Step 5:** Apply the NM method to the best GA solution once again after the GA phase.

**Step 6:** Result - The final parameters ( $\hat{\beta }$ , $\hat{\eta }$ ) obtained from the Nelder-Mead optimization represent the Maximum Likelihood Estimates (MLE) for the Weibull distribution.

## 8. Monte Carlo simulations

The two-parameter Weibull distribution parameter estimation methods were investigated using a Monte Carlo simulation. The scale parameter was set to 1, while the other shape parameters were set to 0.5, 1, 3, and 6. The simulation has been repeated 1000 times for sample sizes of 20, 100, and 500 respectively. With a population size of 100, the GA and GANMA have corresponding crossover and mutation rates of 0.1 and 0.8. The parameters that are used to compare the goodness-of-fit of different parameter estimating methods are mean absolute error (MAE) and bias. For the parameters *β* (shape) and *η* (scale), MAE and bias are computed using the formula provided by:

(For shape parameter)

$$\text{MAE}\left(\hat{\beta }\right)=\frac{1}{n}\sum_{i=1}^{n}\left|\hat{\beta_{i}}-\beta_{i}\right|$$ (15)

$$\text{bias}\left(\hat{\beta }\right)=\frac{1}{n}\sum_{i=1}^{n}\left(\hat{\beta_{i}}-\beta_{i}\right)$$ (16)

(For scale parameter)

$$\mathrm{MAE}\left(\hat{\eta }\right)=\frac{1}{n}\sum_{i=1}^{n}\left|\hat{\eta_{i}}-\eta_{i}\right|$$ (17)

$$\text{bias}\left(\hat{\eta }\right)=\frac{1}{n}\sum_{i=1}^{n}\left(\hat{\eta_{i}}-\eta_{i}\right)$$ (18)

Greater efficiency is implied by lower absolute values of the bias and MAE. For various data sizes and shape parameters, Tables 3- 5 display the parameter estimates, bias, and MAE for each parameter estimation method. The results of the simulation demonstrate that the GANMA approach performed better than NM and GA when estimating shape and scale parameters based on MAE and bias criteria. The best results are highlighted in bold.

**Table 3.** Estimations of parameters, MAE, and bias values for several simulation scenarios with n = 20 of a two-parameter distribution for *β* = 0.5, 1, 3, and 6.

n	*β*	Method		$\hat{\beta }$			$\hat{\eta }$	
			**Mean**	**MAE**	**Bias**	**Mean**	**MAE**	** Bias**
		NM	0.62394	0.12395	0.12393	2.19877	1.19876	1.19871
	0.5	GA	0.60901	0.13641	0.10901	1.76523	0.81895	0.76523
		GANMA	0.60514	**0.10514**	**0.10514**	1.50806	**0.50806**	**0.50806**
		NM	1.21029	0.21028	0.21029	1.22803	0.22810	0.22808
	1	GA	1.23962	0.29000	0.23961	1.380531	0.41456	0.38053
20		GANMA	1.21029	**0.21029**	**0.21009**	1.22803	**0.22803**	**0.22805**
		NM	2.04136	0.95863	-0.95863	1.95863	0.14163	-0.14163
	3	GA	3.63089	0.70843	**0.41377**	1.07488	0.09595	0.074881
		GANMA	3.41374	**0.63089**	0.63088	1.07087	**0.070870**	**0.070870**
		NM	3.08815	2.91184	-2.91146	1.91184	0.08815	-0.08813
	6	GA	5.3846	1.0844	-0.61433	1.05115	0.07196	0.05115
		GANMA	6.26177	**1.06178**	1.26178	1.03482	**0.03482**	**0.034828**

**Table 4.** Estimations of parameters, MAE, and bias values for several simulation scenarios with n = 100 of a two-parameter distribution for *β* = 0.5, 1, 3, and 6.

n	*β*	Method		$\hat{\beta }$			$\hat{\eta }$	
			**Mean**	**MAE**	**Bias**	**Mean**	**MAE**	** Bias**
		NM	0.50000	2.66453	2.88657	1.24999	0.24999	0.24989
	0.5	GA	0.58267	0.08833	-0.04039	1.74933	0.92293	0.67225
		GANMA	0.49477	**0.01522**	**-0.0152**	0.08211	**0.17880**	**-0.17868**
		NM	1.00000	1.7763	1.7322	1.0000	**0.0000**	**0.0000**
	1	GA	0.76607	0.17435	**-0.01683**	0.83393	0.28349	0.11227
100		GANMA	0.97954	**0.03045**	-0.03045	0.90620	0.09379	-0.09386
		NM	2.10606	0.89393	-0.89396	0.89393	0.10606	-0.10606
	3	GA	2.90103	0.45456	**-0.01648**	1.07781	0.09780	-0.08386
		GANMA	2.91863	**0.09136**	-0.09135	0.96769	**0.03230**	**-0.03231**
		NM	2.12499	3.87500	-3.87501	0.87500	0.12499	-0.12497
	6	GA	4.48285	1.10891	-0.93864	0.98534	0.05621	-0.01811
		GANMA	5.51726	**0.18273**	**-0.18273**	0.98371	**0.01628**	**-0.01625**

**Table 5.** Estimations of parameters, MAE, and bias values for several simulation scenarios with n = 500 of a two-parameter distribution for *β* = 0.5, 1, 3, and 6.

n	*β*	Method		$\hat{\beta }$			$\hat{\eta }$	
			**Mean**	**MAE**	**Bias**	**Mean**	**MAE**	** Bias**
		NM	0.50392	0.00392	0.00391	1.24934	0.249345	0.249344
	0.5	GA	0.50555	0.0863	0.0055	1.8336	0.9586	0.8336
		GANMA	0.49826	**0.00677**	**-0.00671**	1.0054	**0.00545**	**-0.00544**
		NM	1.0000	**0.0000**	**0.0000**	1.0000	**0.000**	**0.000**
	1	GA	0.9676	0.1473	-0.0323	1.1998	0.29807	0.19986
100		GANMA	0.98653	0.0134	-0.0133	1.00272	0.00272	-0.00277
		NM	2.0825	0.91747	-0.91746	0.91747	0.08252	-0.08251
	3	GA	3.2000	0.52541	0.20000	1.0404	0.08547	0.0404
		GANMA	2.95959	**0.04040**	**-0.04040**	1.00090	**0.00090**	**-0.00080**
		NM	2.1166	3.88335	-3.88334	0.88335	0.11664	-0.11667
	6	GA	5.2108	1.0329	-0.7891	1.0060	0.06851	0.00605
		GANMA	5.6191	**0.08080**	**-0.080801**	1.0004	**0.00045**	**-0.00043**

### 8.1 Result analysis

**Figure 6.**
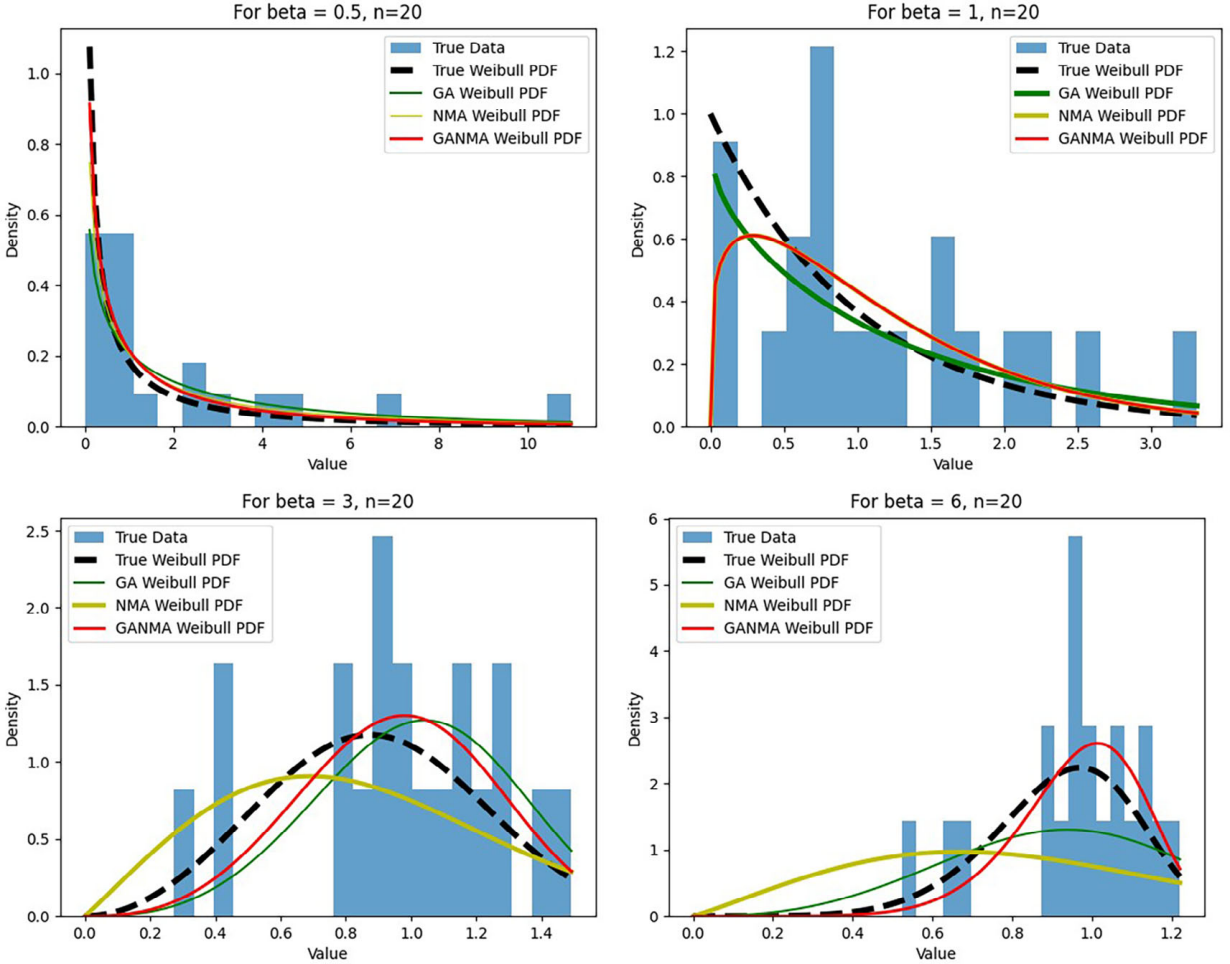
Histogram and MLE PDF of Weibull 2- parameter Distribution for *β* = 0.5, 1, 3, and 6 with n = 20.

**Figure 7.**
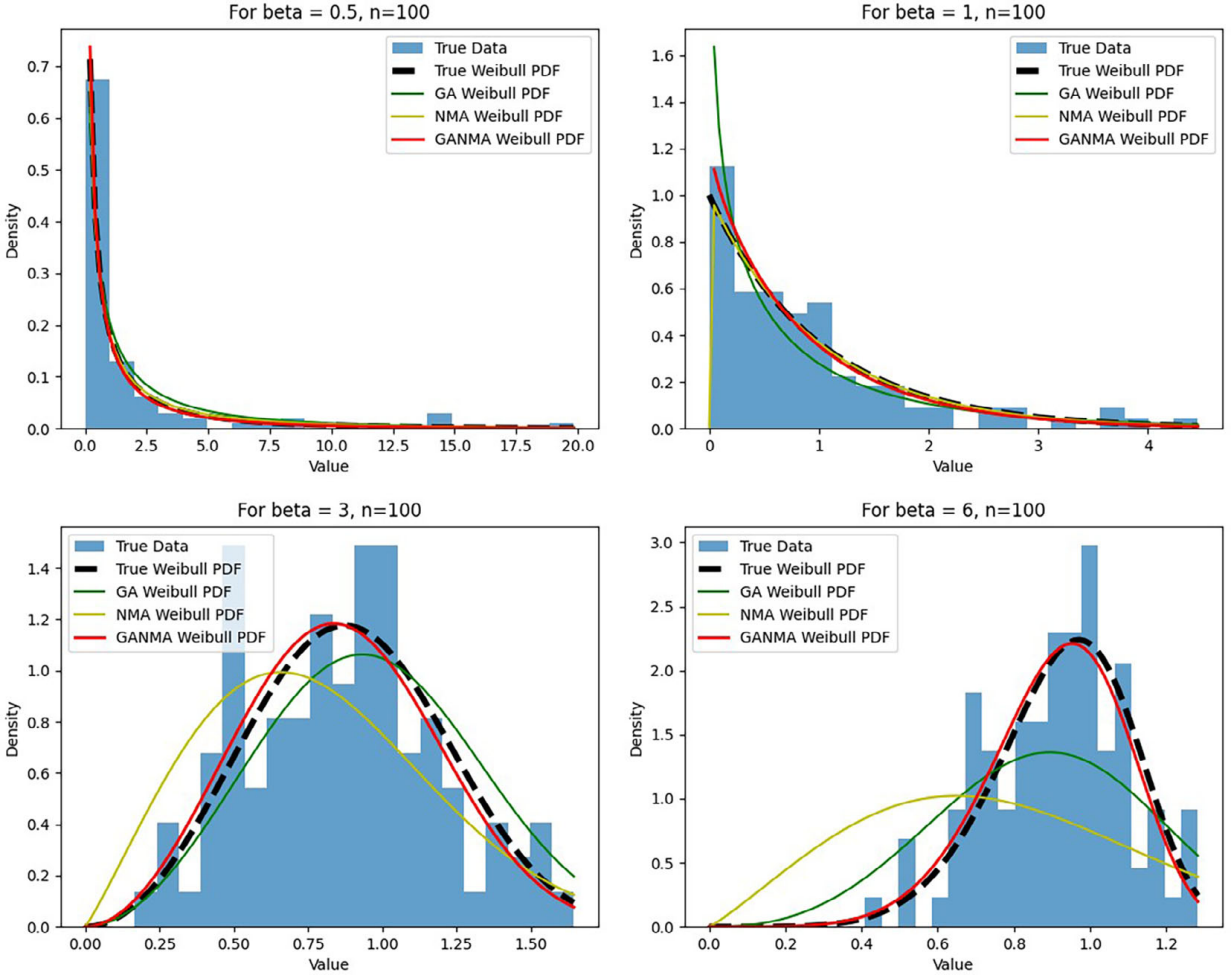
Histogram and MLE PDF of Weibull 2- parameter Distribution for *β* = 0.5, 1, 3, and 6 with n = 100.

**Figure 8.**
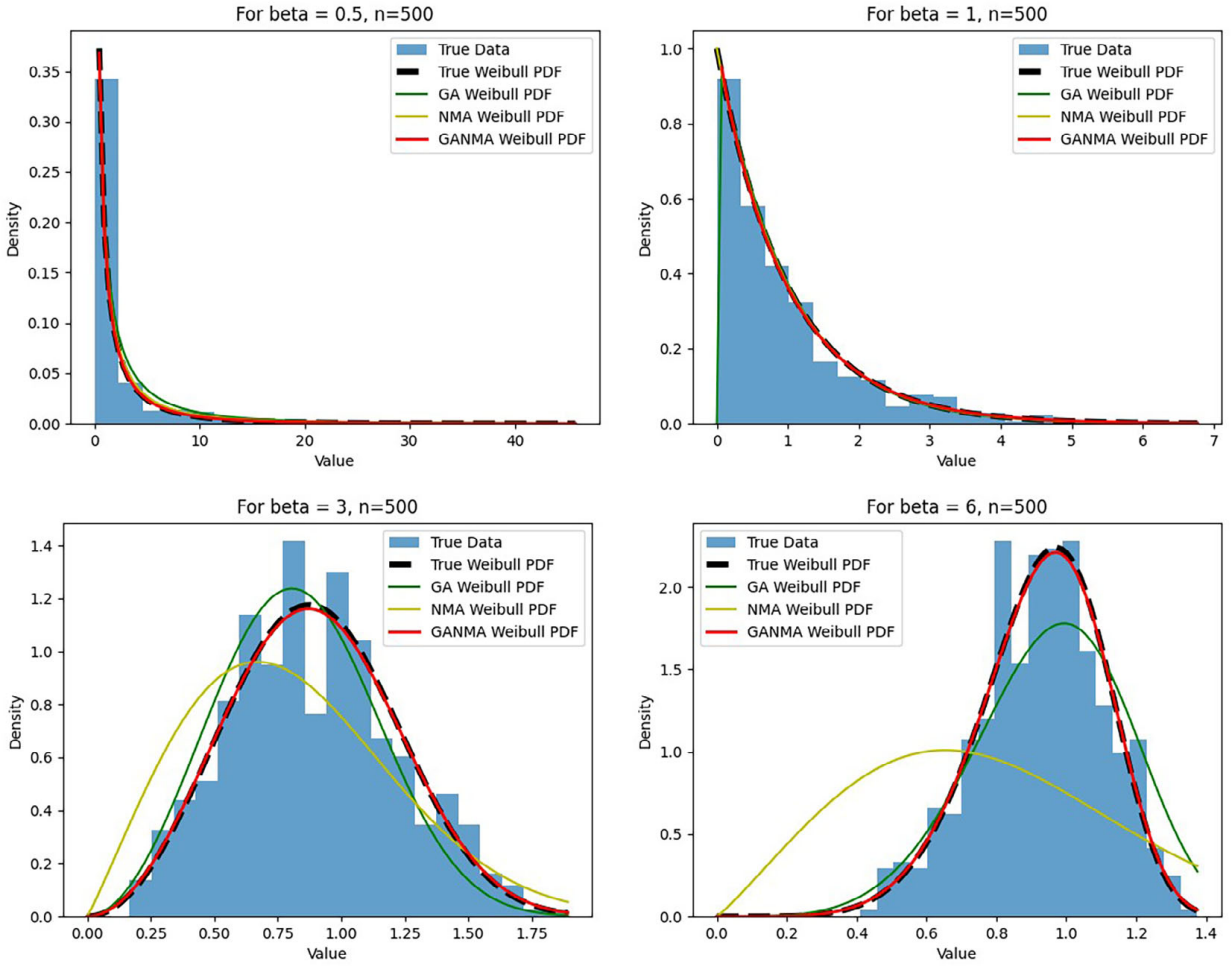
Histogram and MLE PDF of Weibull 2- parameter Distribution for *β* = 0.5, 1, 3, and 6 with n = 500.

Figures 6- 8 illustrate the outcome across various shape parameters while keeping the scale parameter constant—as well as various data sizes by plotting the convergence graph of the PDF of Weibull parameters and the PDF of MLE of parameters using NM, GA, and GANMA. The solid black line depicts the PDF of parameters ( *β*, *η*), whilst the usual genetic algorithm is illustrated by the solid green line, the yellow solid line shows the Weibull PDF using NM, and the suggested method GANMA is shown by the solid red line. It has been found that parameter estimation using the suggested technique converges with the original PDF as the shape parameter and data size increase. GANMA, the suggested algorithm, performs better than GA and NM in all types of situations.

Based on MAE and bias criteria, the simulation results demonstrate that the GANMA technique outperformed NM and GA in the estimation of shape and scale parameters. In each simulated scenario, the GANMA technique yielded the best shape parameter efficiency in terms of bias and MAE for sample sizes of 20, 100, and 500 respectively.

**Figure 9.**
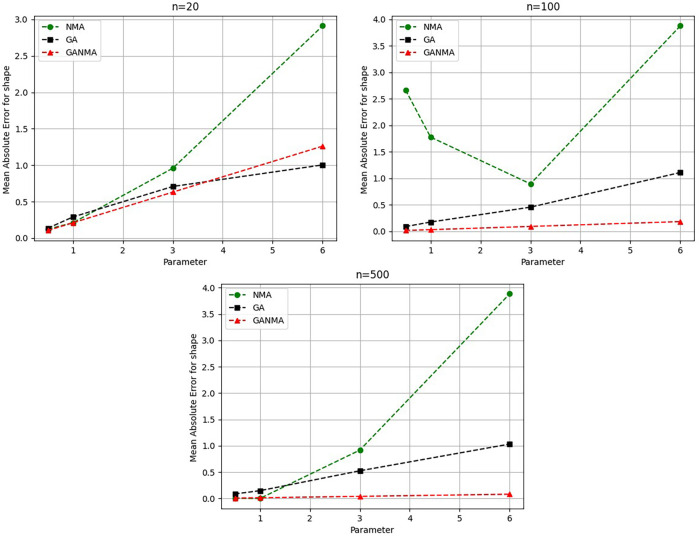
Comparison of parameter estimate approaches for *β* using the MAE criteria.

**Figure 10.**
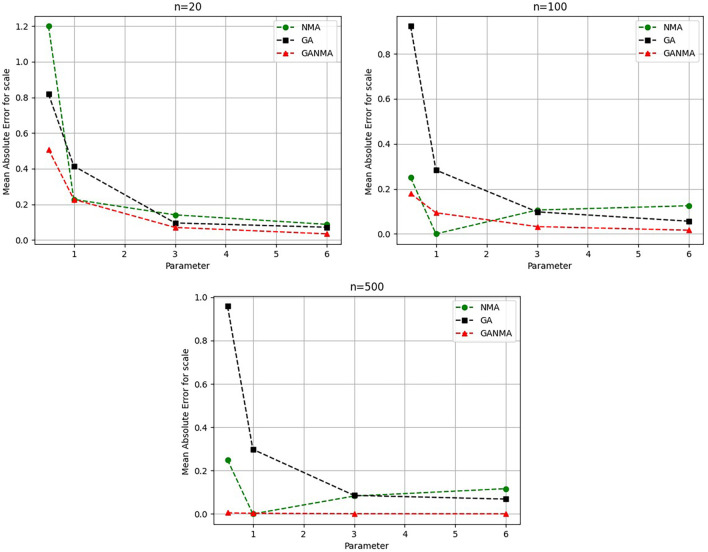
Comparison of parameter estimate approaches for *η* using the MAE criteria.

**Figure 11.**
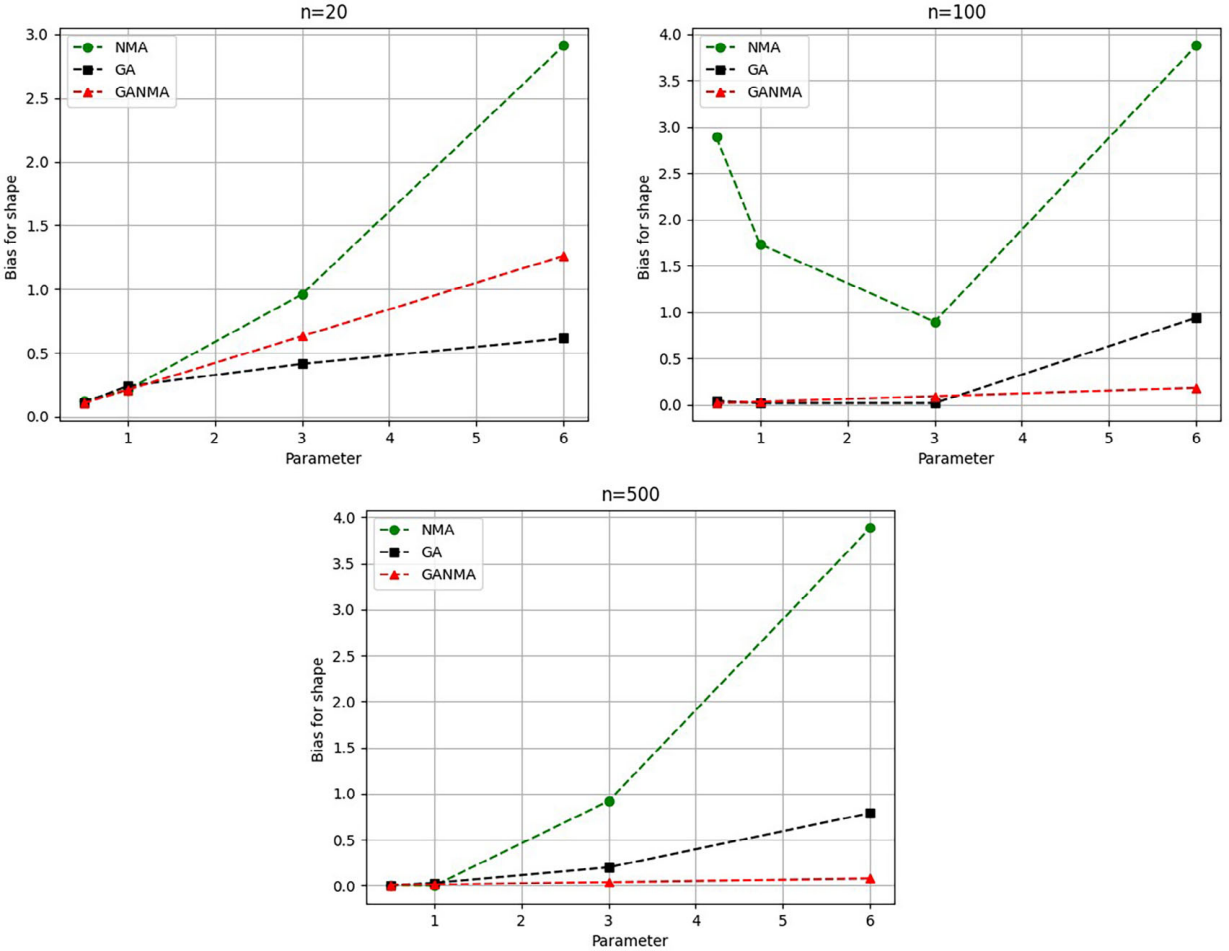
Comparison of parameter estimate approaches for β using the bias criteria.

**Figure 12.**
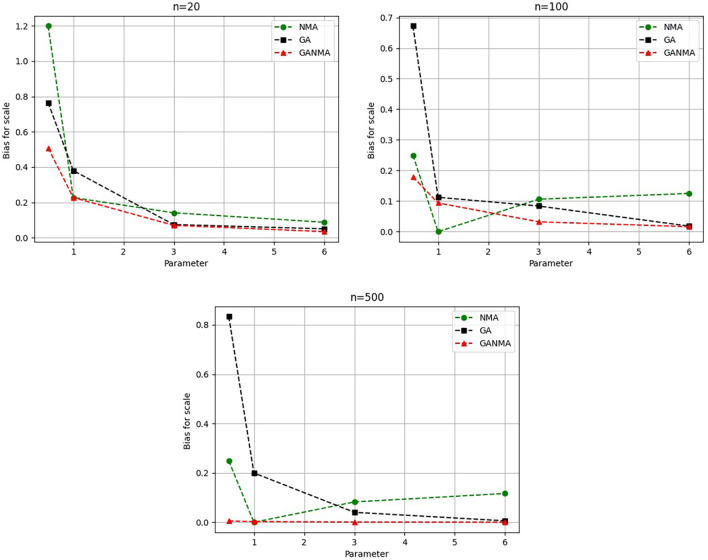
Comparison of parameter estimate approaches for *η* using the bias criteria.

Throughout almost every simulated scenario, GANMA achieved the maximum efficiency in the estimate of scale parameters for sample sizes of 20, 100, and 500, based on at least one decision criterion. By analyzing MAE and bias for each simulation scenario, GANMA proved to be the most effective approach for the data size 20. For small, moderate, and high sample sizes, GANMA is a fairly effective strategy overall. Additionally shown in Figures 9- 12 are the absolute values of the biases and the MAE.

The MAE values for the shape parameter *β* are shown in Figure 9. In every simulated scenario, GANMA outperformed NM and GA in terms of efficiency. The second-best approach is NM. An increase in sample size resulted in lower MAE values. On the other hand, MAE values increased along with an increase in the form parameter value.

The scale parameter *η*’s MAE values are displayed in Figure 10. For sample sizes of 20, 100, and 500, GANMA proved to be the most effective approach. When the shape parameter is set to a higher value, the MAE values drop. Likewise, as the sample size is raised, the MAE values drop.

The shape parameter *β*’s absolute bias value is displayed in Figure 11. The most efficient results were obtained using GANMA. NM outperformed GA on some occasions. As with MAE values, larger sample sizes resulted in lower absolute bias levels. Increasing the parameter value resulted in higher absolute bias levels.

The absolute bias for the scale parameter *η* is shown in Figure 12. Most of the time, GANMA outperformed other methods in terms of efficiency. The second-best approach is NM. Increasing the shape parameter and sample size leads to lower absolute bias levels.

## 9. Estimation of Weibull-Parameter in Wind speed analysis

The decrease in fossil fuel supplies and their lack of reliability in meeting future energy demands have made renewable energy a hot topic for academics. Wind is one of the main sources of renewable energy, and wind speed modeling has been studied in great detail. In wind power applications, the most popular Weibull distribution is two parameters. It has been discovered that this PDF is correct for the majority of wind regimes observed in nature, is easy to use, and is adaptable. In several research, it has been noted that the wind speed data cannot be adequately represented for specific applications, including those with bimodal distributions, short time horizons, low and high wind speeds, and with a high frequency of nulls. ^45^^–^^47^ The given equation may be used to determine the probability density function.

$$f\left(v\right)=\frac{\beta }{\eta }{\left(\frac{v}{\eta }\right)}^{\beta -1}e^{-{\left(v/\eta \right)}^{\beta }}$$ (19)

where *v* is wind speed.

**Power density**

Power density in wind speed analysis refers to the amount of power that can be obtained from the wind per unit area. This statistic is critical when evaluating the feasibility and potential viability of wind energy projects since it quantifies the energy available from the wind at a given place. The power density ( *P _D_*) may be easily calculated using the following equation once *β* and *η* have been established.

$$P_{D}=\frac{\rho_{a}\;\eta^{3}}{2}\;\frac{3}{\beta }\;\Gamma \left(\frac{3}{\beta }\right)$$ (20)

where, $\rho_{a}$ is the air density and symbol Γ denotes the gamma function. The standard value of air density $\rho_{a}$ is taken as $\rho_{a}=1.225\;\text{kg}/m^{3}$ .

### 9.1 Result and Discussion

In this challenge, two real-world data sets have been used to examine wind-speed analysis. The very first set of data came from the seas surrounding the Maluku Islands and Sulawesi. The data under analysis were gathered by the satellite Quikscat, which measured the ocean wind 10 meters above sea level using a scatterometer. The measurement’s horizontal and vertical spatial resolution is 0.25°earth grid. The information from the January measurement point at latitude 116° and longitude 85.5° is included in the accessible data. ^48^

Tarama Island and Iriomote Island, which are close to northern Taiwan, had their wind speeds recorded in the second data set. At Iriomotejima Meteorological Station, the maximum daily wind speed and direction were recorded in March 2012. ^49^

The Kolmogorov-Smirnov (K-S) test is a nonparametric statistical test used to compare two distributions. The K-S test calculates the maximum absolute difference between the empirical cumulative distribution functions (ECDFs) of the distributions being compared, providing a test statistic (D). A p-value derived from this statistic indicates the significance of the difference, helping in goodness-of-fit testing, comparing sample distributions, and model validation without assuming any specific distribution for the data.

The statistical confirmation that the monthly data sets come from the Weibull distribution can be obtained by doing the K-S test separately for each data set. The most significant difference between the theoretical distribution, *S _N_* ( *x*), and the observed distribution, *F*_0_( *x*), is the K-S test statistic. ^50^

$$D=\max|F_{o}\left(x\right)-S_{N}\left(x\right)|$$ (21)

Monthly distributions from the Weibull distribution are selected for further investigation following the K-S test (p−value≥0.05 ), which indicates the probability of observing a discrepancy as large as the one computed if the two distributions were the same.

**Figure 13.**
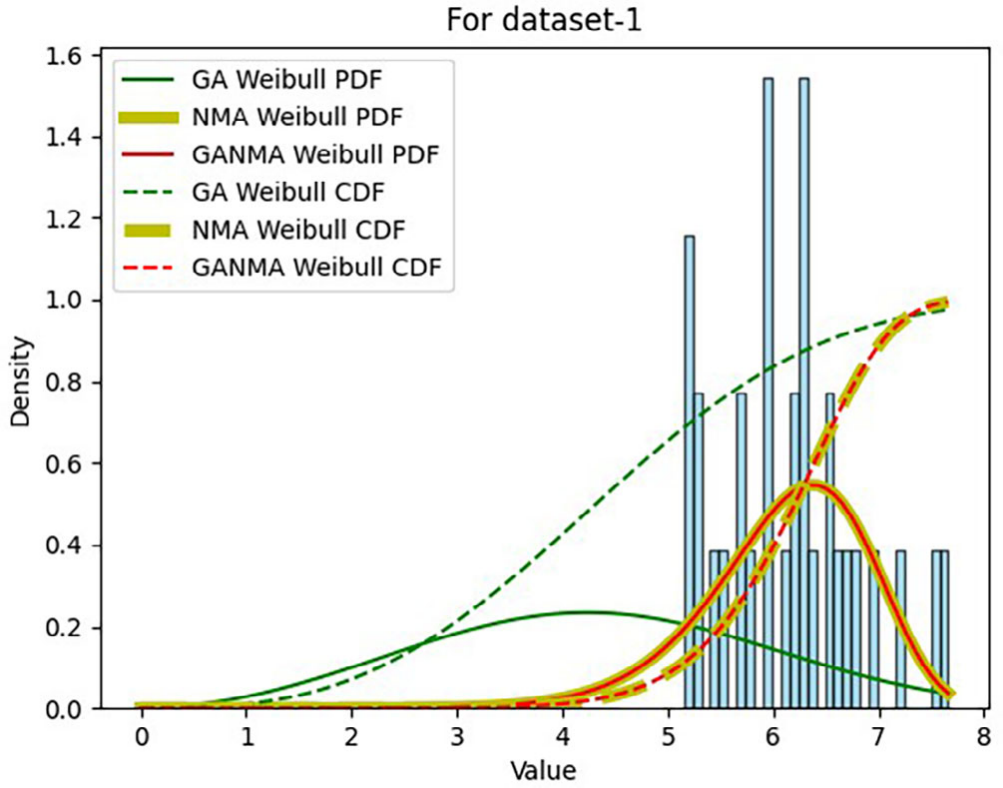
Histogram, MLE PDF and CDF using GA, NM, and GANMA for data set 1.

**Figure 14.**
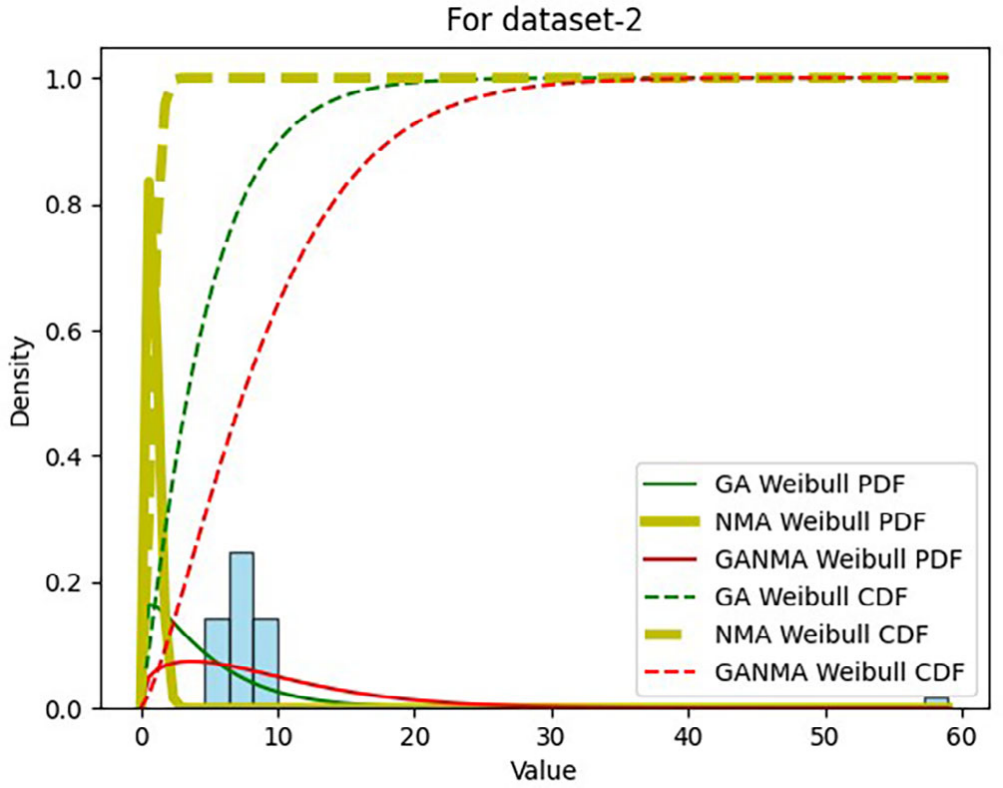
Histogram, MLE PDF and CDF using GA, NM, and GANMA for data set 2.

Results across shape and scale parameters were obtained by plotting the convergence graph between the PDF and CDF of MLE of parameters using NM, GA, and GANMA, as shown in Figures 13 and 14. The solid green line and dotted green line represent the PDF and CDF of the standard genetic algorithm, the yellow solid line, and dotted yellow line represent the Weibull PDF and CDF using NM, and the solid red line and dotted red line represent the suggested method for both the PDF and CDF, respectively. Figure 13 illustrates that the PDF and CDF for both GANMA and NM convergence are on the same line.

Tables 6 and 7 present the shape and scale parameters, k-s value, p-value, and power density for the first and second data sets, respectively, for all three estimation techniques. The greatest p-value and the lowest k-s statistic for both data sets are produced by the suggested approach (GANMA) out of the three estimation techniques. The Weibull distribution and the actual wind speed data seem similar, as indicated by the p-value exceeding the selected significance threshold (e.g., 0.05). In other words, the data is well-fitted by the Weibull distribution. The parameters estimated using GANMA are considered the best fit for describing the wind speed data, based on the K-S test findings. The observed wind speed data and the predicted Weibull distribution with these parameters were well recognized, as evidenced by the low K-S statistic and high p-value.

**Table 6.** Parameter estimations for data set-1.

Method	$\hat{\beta }$	$\hat{\eta }$	k-s value	p-value	P _D_ (watt/m ^2^)
NM	9.52382	6.44868	0.13685	0.56069	147.05921
GA	3.48312	4.97007	0.67756	1.274E-14	71.36770
GANMA	9.52340	6.44863	0.13682	0.560978	147.05541

**Table 7.** Parameter estimations for data set-2.

Method	$\hat{\beta }$	$\hat{\eta }$	k-s value	p-value	P _D_ (watt/m ^2^)
NM	2.0	1.0	0.99999	2.63E-285	0.81422
GA	1.07418	4.97152	0.62512	3.105E-12	350.27224
GANMA	1.35925	9.85611	0.35982	0.00042	1431.63678

The maximum power density is demonstrated by the parameters estimated through MLE implementing NM, as shown in Table 6. This suggests that the parameters possess greater absolute performance in terms of power generation. Despite the slightly lower power density value of the parameters estimated by MLE using GANMA compared to NM, they are nevertheless selected as the best fit since they have the greatest p-value and the least k-s statistic. This suggests that for wind speed data set 1, parameters calculated by MLE using GANMA offer the best match.

The parameters that are estimated by MLE using GANMA are found to provide the best fit in Table 7, as shown by their lowest K-S statistic and highest p-value. Additionally, superior performance in terms of power generation is indicated by the higher power density value associated with these parameters.

## 10. Conclusion

To improve the exploitation capabilities of GA, this study presents a unique hybridized approach called the Genetic and Nelder-Mead Algorithm (GANMA), in which NM is included. GANMA has been employed to verify the robustness and efficiency of the suggested technique on fifteen benchmark problems for three separate dimensions. Because of its high level of accuracy and stability, GANMA performs very well in improving unimodal, multimodal, and shifting unimodal/multi-modal functions, as shown by the test function comparison experiment table. According to the testing results, the suggested method is strong and has the potential to solve benchmark issues more quickly than the other two algorithms in the majority of situations.

Furthermore, estimating the Weibull distribution’s scale ( *η*) and shape ( *β*) parameters, this study aims to assess the efficacy of three estimation methods: ML estimators employing GA, NM, and GANMA. The MAE and bias criteria are used to assess the efficiency of the parameter estimating techniques. Based on the conclusions drawn from the Monte Carlo simulation and the examination of real-world wind speed data, the ML estimator using GANMA performs better in Weibull parameter estimation than the ML estimator using NM and GA estimator. We used the K-S test to compare three sets of parameters for two fitting wind speed data sets with a Weibull distribution and selected the set of parameters that minimized the K-S statistic and maximized the associated p-value, indicating the best fit. Moreover, it may be said that the two sets of data were collected in two different geographic locations with different meteorological conditions. In these data sets, which included a variety of meteorological situations, GANMA demonstrated superiority.

## Compliance with ethical standards

### Disclosures & disclaimer

We certify that the submitted manuscript is our original work that is not currently being considered elsewhere. The paper is an unfunded independent piece of labor.

### Ethical approval

This article does not include any research that any of the authors conducted using humans or animals.

## Data Availability

All data supporting the findings of this study, including figures and tables, have been deposited in the given link; https://doi.org/10.5281/zenodo.13309711 ^51^ The files are as follows: [Data Sets] data has been obtained from a third party for two real-life problems, which are available at data sets.docx The extended data files are available in Zenodo at the following DOI: [ https://doi.org/10.5281/zenodo.13309711.v3] ^51^ [Algorithm] The algorithm described in the manuscript available at algorithm.docx The files included: raw data of functions.docx and raw data of functions (generation wise).docx Contains data analysis that supports the study but is not included in the main manuscript. [Tables] This file contains all the tables referenced in the manuscript. tables.docx [Figures] This file contains all the figures referenced in the manuscript, including detailed captions. figures.docx Data are available under the terms of the Creative Commons Attribution 4.0 International license (CC-BY 4.0).

## Software availability

Python is an open-source source and is available at https://python.org

Archived software is available from: https://doi.org/10.5281/zenodo.13309711 ^51^ (source code.zip) where archived source code can be accessed.
