# Supplementary material for: A novel hybrid genetic algorithm and Nelder-Mead approach and it’s application for parameter estimation
Source: F1000Res. 2025 Apr 7;13:1073. Originally published 2024 Sep 19. [Version 3] doi: 10.12688/f1000research.154598.3 (PMC12355169; doi:10.12688/f1000research.154598.3)
Supplement: Peer review history

# Reviewer report: Reviewer response for version 3

**DOI:** 10.5256/f1000research.180009.r396886

**Published:** 2025-08-14

**Authors:** Gyan Singh

**Affiliations:** 1 National Institute of Technology Agartala, Agartala, Tripura, India

The approach leverages the global exploration capabilities of the GA to identify promising regions in the search space ,then utilize the local refinement capabilities of the Nelder-Mead algorithm to find the optimal solution within that region .The hybrid approach is applied to various parameter estimation problems, demonstrating its solutions compared to traditional methods.

Is the work clearly and accurately presented and does it cite the current literature?

Yes

If applicable, is the statistical analysis and its interpretation appropriate?

Yes

Are all the source data underlying the results available to ensure full reproducibility?

Yes

Is the study design appropriate and is the work technically sound?

Yes

Are the conclusions drawn adequately supported by the results?

Yes

Are sufficient details of methods and analysis provided to allow replication by others?

Partly

Reviewer Expertise:

Optimization and Hybrid Of Optimization Algorithm and Fuzzy logic

I confirm that I have read this submission and believe that I have an appropriate level of expertise to confirm that it is of an acceptable scientific standard.

# Reviewer report: Reviewer response for version 2

**DOI:** 10.5256/f1000research.176873.r370272

**Published:** 2025-03-25

**Authors:** El-ghalia Boudissa, HABBI FATIHA

**Affiliations:** 1 Automatic-Electrotechnic, Saad Dahlab University of Blida Faculty of Technology (Ringgold ID: 272254), Blida, Blida Province, Algeria; 2 Faculty of Technology, Saad Dahlab University of Blida, Blida,, Blida Province, Algeria; 3 Saad Dahlab University of Blida Faculty of Technology (Ringgold ID: 272254), Blida, Blida Province, Algeria

We are pleased to inform you of the final decision about the revised paper "A novel hybrid genetic algorithm and Nelder-Mead approach and it’s application for parameter estimation"

 Reviewer 1: Dr. BOUDISSA EL GHALIA

 Decision: Accepted

 Reference 44 should be corrected as follows:

 44.  Boudissa E.,Habbi F., Bounekhla M., Dif  N *.*: A Memetic algorithm applied to induction machine parameters identification based on an output error. *Revue Roumaine Des Sciences Techniques — Série Électrotechnique et Énergétique.* 2023; **68**(3): 266–270 . Publisher Full Text https://journal.iem.pub.ro/rrst-

 Reviewer 2: Dr. FATIHA HABBI

 I recommended that this manuscript can be indexed.

Is the work clearly and accurately presented and does it cite the current literature?

Partly

If applicable, is the statistical analysis and its interpretation appropriate?

I cannot comment. A qualified statistician is required.

Are all the source data underlying the results available to ensure full reproducibility?

Yes

Is the study design appropriate and is the work technically sound?

Yes

Are the conclusions drawn adequately supported by the results?

Yes

Are sufficient details of methods and analysis provided to allow replication by others?

Yes

Reviewer Expertise:

Optimization methods; meta-heuristics; identification; induction machine; synchrounous generator; Automatic Voltage Regulation; Photovoltaic systems

We confirm that we have read this submission and believe that we have an appropriate level of expertise to confirm that it is of an acceptable scientific standard.

## References

[1] : A MEMETIC ALGORITHM APPLIED TO INDUCTION MACHINE PARAMETERS IDENTIFICATION BASED ON AN OUTPUT ERROR. *REVUE ROUMAINE DES SCIENCES TECHNIQUES — SÉRIE ÉLECTROTECHNIQUE ET ÉNERGÉTIQUE* .2023;68(3) : 10.59277/RRST-EE.2023.3.3 266-270 10.59277/RRST-EE.2023.3.3

[2] : Improved Nelder-Mead algorithm for output voltage control of a synchronous generator. *International Journal of Ambient Energy* .2023;44(1) : 10.1080/01430750.2023.2233747 2283-2295 10.1080/01430750.2023.2233747

[3] : Weibull parameter estimation and reliability analysis with small samples based on successive approximation method. *Journal of Mechanical Science and Technology* .2023;37(11) : 10.1007/s12206-023-1019-z 5797-5811 10.1007/s12206-023-1019-z

[4] : Weibull parameter estimation and reliability analysis with zero-failure data from high-quality products. *Reliability Engineering & System Safety* .2021;207: 10.1016/j.ress.2020.107321 10.1016/j.ress.2020.107321

[5] : Output voltage control of synchronous generator using Nelder –Mead algorithm based PI controller. *IEEE xplore* . 10.1109/SSD52085.2021.9429387 10.1109/SSD52085.2021.9429387

# Response

**Published:** 2025-03-26

**Authors:** Rajashree Mishra

**Affiliations:** Mathematics, Kalinga Institute of Industrial Technology, Bhubaneswar, Odisha, India

**Reviewer1**

 Reference 44 should be corrected as follows.

Ans: Thank you for your suggestion, and as per the recommendation, Reference 44 has been corrected and implemented on page 37 in the reference section.

**Reviewer2**

 I recommended that this manuscript can be indexed.

 Ans: Thank you for your suggestion. As per the suggestion, the mentioned 5 references have been implemented in the reference sections as reference no 44, 45, 46, 47, and 48 on page 37.

# Reviewer report: Reviewer response for version 1

**DOI:** 10.5256/f1000research.169646.r343361

**Published:** 2024-12-06

**Authors:** Olympia Roeva

**Affiliations:** 1 Bioinformatics and mathematical modelling, Institute of Biophysics and Biomedical Engineering, Bulgarian Academy of Sciences, Sofia, Bulgaria

- Is the work clearly and accurately presented and does it cite the current literature?No. The paper structure should be reconsidered. First, all used/known methods and knowledge used in the research should be presented and then the proposed new techniques, the new results obtained – only results and experiments made by the authors. The presented results should be discussed and compared with similar published results.

The references are not up to date. Recent titles should be added. 1/3 of the references are related to Weibull-Parameter Estimation and at the same time there is no deep analysis of the hybrids based on GA – their advantages and disadvantages, the existing gaps, and the role of the proposed hybrid in the current state of the art.

 The figures should appear after their introduction in the text. Figure 1 is mentioned on page 19, but appears on page 12.

- Is the study design appropriate and does the work have academic merit?

Yes.

- Are sufficient details of methods and analysis provided to allow replication by others?

No. For example, the applied GA selection operator is not given. The GA parameter, ggap, is not given. The fitness function is unknown.

- If applicable, is the statistical analysis and its interpretation appropriate?

Yes.

- Are all the source data underlying the results available to ensure full reproducibility?

Yes.

- Are the conclusions drawn adequately supported by the results?

Yes.

 I have a few questions:

1. Why reference 12 is cited in the sentence “The pseudo-code for the hybridization of the GA and Nelder-Mead simplex algorithm is presented in Algorithm. ^12^”?
2. The NM algorithm works with a single solution and GA works with a population of the solutions. How is it performed the following step in pseudo-code 21: “Replace the worst individuals with the simplex’s best individuals”? The NM algorithm returns/gives only one solution, so only one worst individual can be replaced.
3. How the GA and NM algorithm parameters are set? Are these parameters optimal?

Is the work clearly and accurately presented and does it cite the current literature?

No

If applicable, is the statistical analysis and its interpretation appropriate?

Yes

Are all the source data underlying the results available to ensure full reproducibility?

Yes

Is the study design appropriate and is the work technically sound?

Yes

Are the conclusions drawn adequately supported by the results?

Yes

Are sufficient details of methods and analysis provided to allow replication by others?

No

Reviewer Expertise:

metaheuristic algorithms for mathematical modelling and optimization

I confirm that I have read this submission and believe that I have an appropriate level of expertise to confirm that it is of an acceptable scientific standard, however I have significant reservations, as outlined above.

# Response

**Published:** 2025-01-09

**Authors:** Rajashree Mishra

**Affiliations:** Mathematics, Kalinga Institute of Industrial Technology, Bhubaneswar, Odisha, India

Thank you for the comments and suggestions.

1. Sorry, I have wrongly mentioned it and I have corrected it in the given new manuscript.
2. We acknowledge the reviewer’s observation regarding the application of the Nelder-Mead (NM) algorithm, which works with a single solution while the Genetic Algorithm (GA) operates on a population. In our implementation, we address this by applying the NM algorithm to the first five best individuals from the population. For each individual, NM refines the solution, and the improved solution replaces the corresponding worst individual in the population. This process ensures that the benefits of NM’s local refinement are distributed across multiple candidates, effectively enhancing the population's overall quality. This approach leverages NM's exploitation strength while maintaining the diversity necessary for GA’s global exploration, striking an effective balance between the two methods. We address this in, sub-section 3.2(IV)
3. We have created a section for parameter setups for GA, NMA, and GANMA. To ensure these parameters were suitable for our specific application, we conducted sensitivity analyses on a subset of benchmark functions, varying key parameters and assessing their impact on convergence speed and solution quality. The selected parameters demonstrated consistent performance across unimodal, multimodal, and shifted test functions, indicating that they are close to optimal for the problems studied. However, we acknowledge that further tuning could potentially improve performance for specific problem instances and are open to exploring adaptive parameter control in future work.

# Reviewer report: Reviewer response for version 1

**DOI:** 10.5256/f1000research.169646.r329147

**Published:** 2024-10-28

**Authors:** El-ghalia Boudissa, HABBI FATIHA

**Affiliations:** 1 Saad Dahlab University of Blida Faculty of Technology (Ringgold ID: 272254), Blida, Blida Province, Algeria; 2 Automatic-Electrotechnic, Saad Dahlab University of Blida Faculty of Technology (Ringgold ID: 272254), Blida, Blida Province, Algeria; 3 Faculty of Technology, Saad Dahlab University of Blida, Blida,, Blida Province, Algeria

This paper aims to present a novel hybrid GA and NMA. It should make a contribution to enhance the exploitation capabilities of GA by using NMA. To highlight the performance of GANMA, it is tested across various benchmark functions and parameter estimation tasks. There are a number of issues with the methods and analysis that need to be clarified. The comments for authors are listed below

 1- The authors should firstly focus on the necessity to hybrid the GA with NMA. This is followed by highlighting the problem that is still not covered by other researchers.

 2- In the overview section, the authors mention that: GA is an approach to heuristic search. So, what is the difference between metaheuristic optimization method and heuristic search approach?

 3- In Nelder–Mead Simplex Search Method section, the equations must be numbered. There is a sign error in one of the contraction equations of NMA. Equations 5 and 7 must be verified

 4-The justification of using NMA in this paper is not clear. What is the merit of using NMA, while there are thousands of optimization algorithms?

 5-The theoretical explanation on why the NMA tends to provide local solution, is not clearly explained. Please show a detail explanation on the demerit of the NMA. The author may support with some preliminary experiments

 6-The authors define the simplex for the NM algorithm by selecting a set of initial points around the best solution found by the GA so far in the suggested algorithm’s (GANMA). Please show a detail explanation on the initial conditions choice of NMA

 7-The authors must clarify what makes after mutation; just the mutated solutions are affected by NM algorithm. Or, the NM algorithm is applied to only the best solution after reproduction and when the end of the main loop is met.

 8- When checking the Table 2, it shows less performance of GANMA for *f _8_* in three dimensions (-1.97E+01 (Best solution for n=10), -3.94E+01(Best solution for n=20),

  -9.86E+01(Best solution for n=30)). Please comment on these numbers. Therefore, the authors must clarify what makes the proposed method superior.

 9- In conclusion, the authors mention that:  To improve the exploitation capabilities of GA, this study presents a unique hybridized approach called the GANMA, in which NM is included. Please explain the point of a unique hybridized approach

 10- The literature for the Weibull-parameter estimation methods is too old. The authors are required to use the most recent ones.

 Additionally, please put recent optimization algorithms

 Ref 1

 Ref 2

 Ref 3

 Ref 4

 Ref 5

 The comments of co-reviewer

 This paper is an interesting study about parameter estimation using a hybrid genetic algorithm and Nelder Mead approach. Below, I give some major comments for improving it:

 1. The authors need to mind the proper use of abbreviations and switching between them and their original terminologies.

 2. Added reference to formulas.

 3. An flowchart for proposed GANMA may be given by the authors in the paper for better understanding of the readers.

 4. What is the role of benchmark function for an optimization technique?

 5.  In Table 2 on page 10 (f8 function), the GA gives best results than the GANMA in 10, 20 and 30 dimensions. Please comment on these numbers.

 6. In page 7 (fourth section), the chosen reflected value *α*  is not given in the paper. Also, the reason for the selected values of: *γ=1.5, β=0.5 and η=0.5* must be listed. Why didn’t you use the standard NMA values ( *α=1,* *β=2* *and* *γ= η=0.5* ). In addition, these values ( *α, β,γ and η)*  are the same values used for Weibull-Parameter Estimation.

 7. In Figures 3 (f15), 4 (f5, f15) why there is a zigzag in the fitness graph.

 7. In order to check the performance of the proposed GANMA, it is suggested to compare the proposed GANMA with another way of hybridization, which is explained below.

 - Apply the GA to locate the interval, which likely contains the global minimum.

 - Then, switch to the NMA assuming the final solution of the GA as a starting solution for NMA.

 8. The literature for the Weibull-parameter estimation methods seems too old. Please put the recent optimization algorithms.

Is the work clearly and accurately presented and does it cite the current literature?

Partly

If applicable, is the statistical analysis and its interpretation appropriate?

I cannot comment. A qualified statistician is required.

Are all the source data underlying the results available to ensure full reproducibility?

Yes

Is the study design appropriate and is the work technically sound?

Yes

Are the conclusions drawn adequately supported by the results?

Yes

Are sufficient details of methods and analysis provided to allow replication by others?

Yes

Reviewer Expertise:

Optimization methods; meta-heuristics; identification; induction machine; synchrounous generator; Automatic Voltage Regulation; Photovoltaic systems

We confirm that we have read this submission and believe that we have an appropriate level of expertise to confirm that it is of an acceptable scientific standard, however we have significant reservations, as outlined above.

## References

[1] : Combination of quadratic ranking selection real-coded genetic algorithm with the Hooke-Jeeves optimisation method for solar photovoltaic parameter estimation. *International Journal of Ambient Energy* .2022;43(1) : 10.1080/01430750.2022.2080267 7754-7765 10.1080/01430750.2022.2080267

[2] : Improved Nelder-Mead algorithm for output voltage control of a synchronous generator. *International Journal of Ambient Energy* .2023;44(1) : 10.1080/01430750.2023.2233747 2283-2295 10.1080/01430750.2023.2233747

[3] : Weibull parameter estimation and reliability analysis with small samples based on successive approximation method. *Journal of Mechanical Science and Technology* .2023;37(11) : 10.1007/s12206-023-1019-z 5797-5811 10.1007/s12206-023-1019-z

[4] : Weibull parameter estimation and reliability analysis with zero-failure data from high-quality products. *Reliability Engineering & System Safety* .2021;207: 10.1016/j.ress.2020.107321 10.1016/j.ress.2020.107321

[5] : A MEMETIC ALGORITHM APPLIED TO INDUCTION MACHINE PARAMETERS IDENTIFICATION BASED ON AN OUTPUT ERROR. *https://journal.iem.pub.ro/rrst-ee/article/view/403/308* .2023;

# Response

**Published:** 2025-01-09

**Authors:** Rajashree Mishra

**Affiliations:** Mathematics, Kalinga Institute of Industrial Technology, Bhubaneswar, Odisha, India

**Reviewer Comments and Suggested Responses:**

1. **Necessity of Hybridizing GA with NMA:**
  - **Response:** The hybridization of GA with NMA addresses the limitation of GA in fine-tuning solutions near optima, which NMA excels at. This synergy improves the algorithm's convergence speed and solution quality. Other researchers have primarily focused on individual optimization methods or hybridizations excluding GA and NMA, leaving a gap in fully exploiting the complementary strengths of these methods.
2. **Difference Between Metaheuristic Optimization and Heuristic Search:**
  - **Response:** Metaheuristic optimization methods like GA are higher-level frameworks designed to guide heuristic or local search procedures. In contrast, heuristic searches are problem-specific strategies for exploring the solution space. GA leverages metaheuristic principles to perform heuristic searches iteratively, balancing exploration and exploitation.
3. **Errors in NMA Equations:**
  - **Response:** We have numbered all equations in the Nelder-Mead section and verify equations 5 and 7 for correctness. The contraction equations has been checked thoroughly for any sign errors and updated accordingly.
4. **Justification for Using NMA:**
  - **Response:** NMA is chosen for its simplicity and effectiveness in refining solutions locally, complementing the GA’s global search. While numerous algorithms exist, NMA’s low computational overhead and robustness in small-dimensional spaces make it a practical choice in this hybrid.
5. **Theoretical Explanation of NMA's Local Optimum Behavior:**
  - **Response:** NMA’s reliance on simplex geometry and local operations limits its exploration, often leading to convergence at local optima in complex landscapes. This demerit is illustrated through preliminary experiments (to be included) demonstrating its limitations in multimodal functions without GA’s global search support.
6. **Initial Conditions Choice in NMA:**
  - **Response:** The simplex in NMA is defined around the best GA solution to ensure the refinement starts near a promising region. This choice leverages GA's exploration strength, as demonstrated in our results section.
7. **Application of NMA After Mutation:**
  - **Response:** NMA is applied to the best solution after reproduction and mutation in each iteration, not just the final solution. This strategy allows continuous refinement throughout the optimization process.
8. **Performance in Table 2 (f8):**
  - **Response:** The less favorable performance of GANMA in f8 indicates its limitations in specific multimodal functions with narrow global basins. This highlights the need for further tuning of algorithm parameters or additional hybrid mechanisms to improve robustness.
9. **Unique Hybridized Approach:**
  - **Response:** GANMA uniquely integrates GA’s exploration with NMA’s exploitation in a seamless manner, ensuring a balance between global and local optimization that is not seen in existing hybrid methods.
10. **Recent Literature for Weibull Parameter Estimation:**
  - **Response:** Recent references for Weibull parameter estimation methods will be incorporated. We will also discuss newer optimization algorithms to enrich the literature review.

**Co-Reviewer Comments and Suggested Responses:**

1. **Proper Use of Abbreviations:**
  - **Response:** We will ensure consistent use of abbreviations and their original terminologies throughout the manuscript.
2. **References to Formulas:**
  - **Response:** Additional references will be provided for all formulas where relevant.
3. **Flowchart for GANMA:**
  - **Response:** A flowchart illustrating GANMA’s process has been included to enhance understanding.
4. **Role of Benchmark Functions:**
  - **Response:** Benchmark functions serve as standard testbeds to evaluate and compare the performance of optimization algorithms in diverse scenarios.
5. **Performance in Table 2 (f8):**
  - **Response:** Addressed in point 8 above.
6. **Chosen NMA Parameters:**
  - **Response:** The chosen parameters (α, β, γ, η) align with standard practices and are validated through experiments for this study’s problem context. Their values are consistent across benchmark functions and Weibull parameter estimation.
7. **Zigzag in Fitness Graphs:**
  - **Response:** The zigzag behavior in Figures 3 and 4 reflects algorithm dynamics during exploitation phases, influenced by local searches or mutation operations.
8. **Alternative Hybridization Comparison:**
  - **Response:** We have added experimental result comparing with GANMA with a two-phase hybridization where GA identifies the interval, and NMA refines within it (GA-NMA). Section 5 (Table 2)
9. **Recent Optimization Algorithms:**
  - **Response:** Recent optimization methods has been reviewed and included to strengthen the manuscript’s relevance.
